# Choice of Processing Pipelines for T1‐Weighted Brain MRI Impacts Association and Prediction Analyses

**DOI:** 10.1002/hbm.70372

**Published:** 2025-10-30

**Authors:** Elise Delzant, Olivier Colliot, Baptiste Couvy‐Duchesne

**Affiliations:** ^1^ Sorbonne Université, Institut du Cerveau – Paris Brain Institute, CNRS, Inria, Inserm, AP‐HP, Hôpital de la Pitié‐Salpêtrière Paris France; ^2^ Institute for Molecular Bioscience, The University of Queensland St Lucia Queensland Australia

## Abstract

The growing availability of large neuroimaging datasets, such as the UK Biobank, provides new opportunities to improve robustness and reproducibility in brain imaging research. However, little is known about the extent to which MRI processing pipelines influence results. Using 39,655 T1‐weighted MRI scans from the UK Biobank, we systematically compared five widely used gray‐matter representations derived from three major software packages: FSL (volume‐based), CAT12/SPM (volume‐ and surface‐based), and FreeSurfer (cortical and subcortical surface‐based). We assessed their impact on morphometricity (trait variance explained by brain features), susceptibility to imaging confounders, false positives, association findings, and prediction accuracy across 29 diverse traits, including lifestyle, metabolic, and disease‐related variables. We found that all pipelines were sensitive to imaging confounders such as head motion, brain position, and signal‐to‐noise ratio, and many produced non‐normal voxel or vertex distributions. FSL and FreeSurfer generally yielded higher morphometricity estimates, but each captured partially unique signals, leading to inconsistencies in brain regions identified across methods. Volume‐based approaches tended to outperform surface‐based ones, detecting more significant clusters, achieving higher replication rates, and producing stronger predictive performance. Small clusters (single voxels or vertices) were less reliable, suggesting caution in their interpretation. Among all methods, FSLVBM emerged as the most consistent all‐rounder, maximizing morphometricity, replicability, and predictive accuracy. Our results highlight the strengths and limitations of commonly used processing pipelines, offering benchmarks to guide researchers in method selection. They further suggest that combining multiple pipelines may improve brain‐based prediction by leveraging unique, complementary signals, and that careful treatment of imaging confounders is essential for robust large‐scale neuroimaging analyses.

## Graphical abstract

Using 39,655 UK Biobank brain MRIs, we compared five gray‐matter processing pipelines across 29 traits. FSLVBM emerged as the most robust, maximizing morphometricity, replicability, and predictive accuracy. Our findings highlight significant differences in pipelines, emphasizing the need for careful selection and ensemble approaches to enhance neuroimaging robustness and reproducibility.

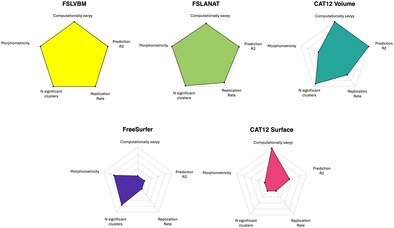

## 1 Introduction

Brain MRI (magnetic resonance image) can provide direct insights into the brain structures, regions, or functions that are associated with behavior but also with disorders of the brain. Additionally, the emergence of large datasets becoming available allows us to perform analysis at an unprecedented scale. These datasets could help tackle the reproducibility crisis in the neuroimaging field, as it has been partly attributed to the small sample sizes (Marek et al. 2022). For example, the UK Biobank has released MRI from over 40,000 volunteers, together with detailed information about chronic disorders, lifestyle, and behavior. Beyond new knowledge about the brain, the UK Biobank can help us progress toward more robust methodology and in particular understand how analytic choices may influence results.

T1w brain MRI provide a detailed mapping of the gray matter structure and can be processed using different imaging software. There are two main representations of the gray matter: the first one is volume‐based (also called Voxel‐Based Morphometry pipeline –VBM; Ashburner and Friston 2000), which quantifies the gray matter density for each voxel (3D pixel). In comparison, surface‐based approaches project a mesh over the gray matter to examine (Antonopoulos et al. 2023) cortical thickness, surface area, or volume at the vertex level. Despite their widespread utilization and long‐term use, there are no guidelines to choose from for the imaging pipelines, leading to a lack of robustness in the results. Robustness to processing methods can be defined as the ability to identify consistent findings across variations in methods (for a unique dataset) (Botvinik‐Nezer and Wager 2023). Robustness complements reproducibility (the ability to identify consistent findings with the same method and data) and replicability (the ability to identify consistent findings across datasets, using the same method). Previous work has highlighted the variability induced by different surface‐based processing pipelines (Bhagwat et al. 2021) on structural MRI: indeed, by examining sex differences and age‐related changes, they showed that processing identified considerably different regions, as well as low similarity between processed gray matter brain maps. Similarly, when comparing VBM pipelines, a previous study showed that all pipelines resulted in slightly different brain measurements for the same individual and that the choice of image processing impacted age prediction and sex classification (Zhou et al. 2022). It is therefore important to determine and quantify how the choice of processing software affects neuroimaging results to ultimately improve robustness to processing methods. Notably, the challenge of robustness is not unique to T1w processing but is also a common concern in other neuroimaging modalities (Botvinik‐Nezer and Wager 2023), such as functional neuroimaging (Carp 2012).

The multiverse approach has been proposed to overcome the robustness issue (Carp 2012): a single analyst or collaborative teams perform analyses with the same data but different pipelines, and the results are compared or merged. However, applying this method to large datasets (e.g., the UK Biobank) is computationally demanding and would entail so much image processing that the multiverse cannot be extensively explored. In addition, combining analyses performed on volumes and surfaces is challenging, as there is no simple way to overlay the association maps. Instead of reconciling all the branches of the multiverse, another option may be to compare the branches to identify the best one(s) or prune the least efficient ones. Recently, Furtjes et al. used morphometricity to compare different atlas‐based representations of gray‐matter structure in the UK Biobank. They showed that more detailed atlases captured more information (larger morphometricity) (Fürtjes et al. 2023). Similarly, morphometricity has been used to compare the amount of information captured by different cortical mesh and varying levels of smoothing in surface‐based processing (Couvy‐Duchesne et al. 2020).

If morphometricity can help benchmark multiverse branches to identify the MRI processing that retains the most information, other criteria are to be considered, depending on the study objectives. For example, Brain‐Wide Association Study (BWAS) aims to assess the relationship between a particular trait and each brain measurement, and its power to detect associations depends on the morphometricity as well as other factors such as sample size (Marek et al. 2022), multiple testing correction methods (Nichols 2012) or trait complexity (i.e., how many brain regions contribute to the morphometricity). Similarly, brain‐based prediction is also influenced by the training sample size and the number of brain features, which warrants more detailed evaluation.

We sought to benchmark some of the main branches of the multiverse of T1w MRI processing to evaluate the robustness of findings across processing. We conducted analyses utilizing five standard and commonly used high‐dimensional representations (voxel‐based or vertices‐based) of the gray matter. These T1w processings correspond to the 3 most common neuroimaging software for structural MRI, with default options and off‐the‐shelf implementations. We compared the gray‐matter representations in terms of morphometricity, but we also examined their sensitivity to imaging and sample confounders, their false positive rate, as well as their ability to detect replicable associations in brain‐wide association studies or to develop brain‐based predictors. We considered 29 different traits of interest to evaluate how results generalize across traits and diseases, and we used 39,655 T1w images from the UK‐Biobank. Our results should help researchers make informed decisions about MRI processing options to study gray matter structures, which might depend on their trait of interest or study objectives. Our results may also guide the exploration of the multiverse by revealing the most promising branches.

## 2 Material and Methods

### 2.1 UK Biobank Data

We considered all participants from the UK Biobank who had undergone a brain MRI exam at the time of data extraction. Of note, the UK Biobank is a prospective cohort that comprises more than 500,000 volunteers across the United Kingdom. The only exclusion criteria are age (< 40 or > 69 years old), so participants can be either healthy or diagnosed with a disease when included in the database. Moreover, it is not a representative sample of the United Kingdom population, with an over‐representation of healthy (fewer diagnoses/conditions reported) and educated individuals (Fry et al. 2017). We included only the first imaging scan when several were available.

T1w images were acquired in 4 different centers: the first one (Cheadle) started scanning in 2015, while the three other centers (Newcastle, Reading) started scanning in 2017 and the Bristol center started scanning in 2020. All centers are equipped with a standard 3T Siemens Skyra, a 32‐channel Siemens head RF coil, and software (VD13) and follow the same protocol. T1w 3D MPRAGE is acquired with a resolution of 1 × 1 × 1 mm, during 5 min, in a sagittal plane, in‐plane, acceleration iPAT = 2, and a prescan‐normalize option.

**FIGURE 1:**
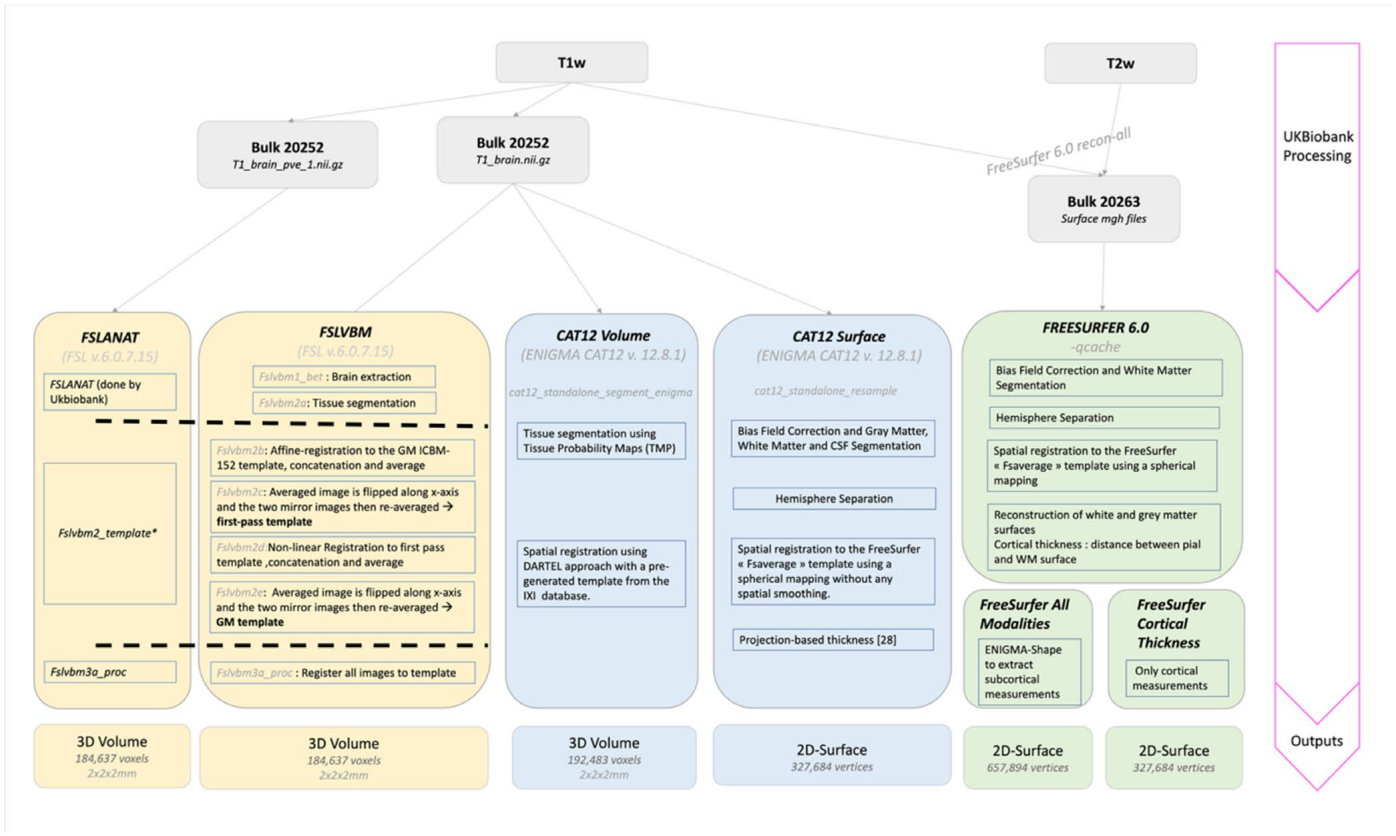
Overview of the six processing pipelines. We considered three voxel‐based morphometry pipelines (FSLANAT, FSLVBM, CAT12 Volume) and two surface‐based morphometry pipelines (CAT12 Surface and FreeSurfer). We downloaded bulk files (individual zip files used to access large and/or complex items such as imaging data) provided by the UK Biobank and performed the additional processing steps to extract the vertex or voxel wise data. FreeSurfer software (in green) was split into two pipelines: One including only cortical thickness measurements and another one including all cortical and subcortical measurements.

We excluded from our analysis participants labeled as “unusable” (Alfaro‐Almagro et al. 2021) by the UK Biobank (due to low‐quality MRI) and the one who opted out of the study. The assessment of T1 data for usability involves an automated quality control (QC) process for all T1 datasets, and has been described in the imaging reference paper (Alfaro‐Almagro et al. 2018). We selected individuals with a usable T1w structural brain image (data field 20252) and available T1 surface files (data field 20263), which served as input of the processing (Figure 1). We excluded participants whose T2‐FLAIR was not used (because deemed unusable) in the FreeSurfer processing conducted by the UK Biobank.

Our final sample comprised 39,826 participants, which we split into a discovery and a replication sample based on their assessment center. The discovery/main sample consisted of participants from the first imaging acquisition center (Cheadle, Greater Manchester): 23,288 adults, aged 63.1 on average (SD = 7.5) with 52% women. The replication/validation sample consisted of 16,538 adults, aged 62.2 on average (SD = 7.7) with 54% women collected across the three other imaging centers (Reading, Newcastle, and Bristol). The discovery/main dataset was used for the main analyses, and we used the replication/validation to replicate associations or evaluate the prediction of models trained in the discovery/main sample.

Informed consent was obtained from all UK Biobank participants. Procedures are controlled by a dedicated Ethics and Guidance Council (http://www.ukbiobank.ac.uk/ethics). IRB approval was also obtained from the North West Multi‐Center Research Ethics Committee. This research has been conducted using the UK Biobank Resource under Application Number 53185.

### 2.2 Brain MRI Processing

We processed all our images with five different pipelines (Figure 1). Two used surface‐based representations of the gray‐matter structure (vertices), while the other three used volume‐based representations (voxels).

### 2.3 FSLVBM and FSLANAT

We processed skull‐extracted T1w images (from data field 20252) with FSL (Jenkinson et al. 2012) in two ways that appeared to give slightly different brain measurements for the same individual in a previous paper (Zhou et al. 2022). The first one (FSLVBM, right yellow pipeline, Figure 1) performs the full FSL pipeline from the skull‐extracted T1w image (*T1_brain.nii.gz*, Figure 1), while the second one (FSLANAT, left yellow pipeline, Figure 1) takes as input the brain segmentation already performed by the UK Biobank (*T1_brain_pve_1.nii.gz*, Figure 1) to reduce processing time. The main difference between the two processings lies in the order of the processing steps. In short, FSLANAT registers the brain into a common space (MNI) before segmenting the gray matter. On the other hand, FSLVBM segments the gray matter in the native space before registering the segmented images to MNI. In both processings, images are non‐linearly registered to a study‐specific template. Since it was not computationally possible to create a template with all the images, we used templates generated from 600 individuals balanced for sex and acquisition site. We found that increasing the template size further had little effect on the processed scans (see Figure S1 for more details). The resulting images of both FSL processings have dimensions 91 × 109 × 91 voxels, each of size 2 mm.

### 2.4 CAT12 Volume‐Based and Surface‐Based

We applied another processing pipeline to the T1w skull‐extracted images (*T1_brain.nii.gz*, from data field 20252) created by the ENIGMA consortium, which relies on the Computational Anatomy toolbox CAT12.8.1 for SPM12 (Gaser et al. 2022). We used the standalone version of the ENIGMA CAT12 toolbox that does not require a MATLAB license. The toolbox can output voxel‐based (volume) and vertex‐based (surface) representations of the gray‐matter structure. The volume‐based method (referred to as CAT12 Volume, left blue pipeline, Figure 1) uses the output from the standard SPM “unified segmentation.” Our final output is the modulated‐warped‐GM‐partial volume segmentation (*mwp1.nii.gz*). Each image has a size of 85 × 103 × 85 voxels, each of size 2 mm.

The surface‐based approach (referred to as CAT12 Surface, right blue pipeline, Figure 1) extracts cortical thickness using a projection‐based method (Dahnke et al. 2013), performed on the outputs of the volume‐based processing described above. We obtained 163,842 vertices that measure local cortical thickness for each hemisphere.

### 2.5 FreeSurfer

The UK Biobank team processed the T1w and T2w (FLAIR) images using FreeSurfer 6.0 (Fischl 2012), and we downloaded the resulting processed images, available in bulk data 20,263 (Figure 1). In short, the processing utilized T1w and T2w (FLAIR) images together, which improves pial surface reconstruction.

From the downloaded bulk data, we ran the recon‐all ‐qcache option (Sufer, n.d.) to obtain the surface files. In addition, we applied the ENIGMA‐shape protocol (Gutman et al. 2012, 2013) to the output of the FreeSurfer processing, which computes vertex‐wise measurements (radial thickness and log‐jacobian, analogous to a surface area) for seven subcortical structures. We obtained 163,842 vertices for each cortical hemisphere and modality (thickness and surface area) and 13,560 vertices for the subcortical volumes for each type of measurement (radial thickness and surface area). Overall, “FreeSurfer All Modalities” (bottom‐left green pipeline, Figure 1) includes all cortical and subcortical measurements (thickness and surface area). We additionally consider in the following analysis “FreeSurfer Cortical Thickness” (bottom‐right green pipeline, Figure 1), which only retains cortical thickness measurements to facilitate comparison to CAT12 Surface outputs.

### 2.6 Voxels and Vertices QC

For all three volume‐based processing (FSLANAT, FSLVBM, CAT12‐Volume), we excluded the voxels with mean lower than 0.1 and variance lower than 0.01, to retain the gray‐matter voxels that are non‐null across most participants. This resulted in 181,544 voxels for the FSLVBM pipeline, 184,637 voxels for the FSLANAT method, and 192,483 voxels for the CAT12 volume‐based processing (Figure S2).

For all three surface‐based processing (CAT12‐Surface, FreeSurfer Cortical Thickness and FreeSurfer All Modalities), we excluded the vertices that contained 0 for all participants. This resulted in 299,881 vertices for CAT12 Surface and FreeSurfer Cortical Thickness, and 654,002 vertices for FreeSurfer All Modalities.

We exported all brain measurement tables into .bod format (a binary format) to optimize the memory requirement and computational time of analyses with OSCA (OmicS‐data‐based Complex trait Analysis) (Zhang et al. 2019) software.

### 2.7 SumR^2^ , Kurtosis and Skewness of Measurements

We sought to quantify the amount of correlation between the brain measurements of each processing, which reflects the inherent smoothness of the data, and the strength of the connectome between correlated brain regions. We regressed out the covariates from the vertices/voxels values, as they might increase the correlation between brain measurements. Thus, we computed (for each processing), for each *i*‐th vertex/voxel the sum of all *R*_*i,j*_^2^ (square of Pearson's correlation between *i*‐th vertex/voxel and *j*‐th one), with *j* varying from 1 to *p* (*p* being the total number of vertices/voxels given processing). The sum of *R* ^2^ (sum*R* ^2^_*i*_) for the *i*‐th voxel/vertex quantifies the amount of correlation with all other brain measurements:

(1) $$\mathrm{Sum}R_{i}^{2}=\sum_{j=1}^{p}R_{i,j\;\text{adjusted}}^{2}$$

where $R_{\text{adjusted}}^{2}=R^{2}-\frac{1-R^{2}}{N\_2}$ is the unbiased estimator of *R* ^2^, since the standard estimator of the Pearson correlation has upward bias of approximately 1/*N* (with 𝑁 being the sample size). To reduce computation, we calculated sum*R* ^2^ using a subset of 1000 UK Biobank individuals, representative in terms of site and sex.

In addition, we calculated the kurtosis and skewness of each vertex/voxel measurement to evaluate departures from normality in the distributions. Of note, kurtosis is a measure of the “tailedness” of the distribution, and skewness is a measure of the asymmetry.

### 2.8 Parcellation of Vertex/Voxels Using Cortical, Subcortical and Cerebellar Atlases

We used complementary atlases to annotate voxels and vertices across the different gray‐matter regions (cortical, cerebellar, and subcortical). For the cortical voxels/vertices, we used the Jülich‐Brain atlas v3.0.3 (Amunts et al. 2020), which is provided for both volume‐based and surface‐based processing. Indeed, this atlas is available in FreeSurfer FsAverage space and in Colin 27 (volume‐based) space. The Jülich‐Brain atlas comprises 157 different regions of interest.

For the subcortical nuclei in volume‐based processing, we used the Harvard‐Oxford atlas (Makris et al. 2006) provided by FSL and comprising 21 regions of interest (e.g., hippocampal subfields) aligned to the MNI‐152 NLIN template. For surface‐based processing, the vertices are associated with one of 7 subcortical nuclei (e.g., hippocampus) as part of processing.

For the cerebellum (only mapped in volume‐based processing), we used the Dierdrichsen atlas (Diedrichsen et al. 2009), also provided by FSL, aligned to the MNI‐152 NLIN template and resulting in 28 anatomical structural regions.

All three Harvard‐Oxford (subcortical atlas), Dierdrichsen (cerebellar atlas), and FSL processings are registered in the MNI‐152 NLIN coordinate system (Grabner et al. 2006). However, CAT12 Volume (SPM) uses a slightly different space (MNI 152 linear) (Mazziotta et al. 1995). Therefore, we projected the Harvard‐Oxford and Dierdrichsen MNI coordinates into the SPM space to obtain a subcortical and a cerebellar atlas aligned to the SPM voxels. Similarly, the Julich MNI space is also different (Colin 27), and we projected the atlas to FSLs and SPM coordinates. To achieve that, we used some co‐registration functions provided by ANTs (Avants et al. 2011) (*antsRegistration, antsApplyTransforms*) and MrTrix3 (Tournier et al. 2019) (*mrtransform*) software.

### 2.9 Morphometricity

We estimated, for each processing, the percentage of trait variance captured by all brain features (vertices or voxel measurements), which has been coined “morphometricity” (Sabuncu et al. 2016). To estimate this morphometricity, we fitted a linear mixed model (Couvy‐Duchesne et al. 2020) (Appendix S1). The linear mixed model is implemented in OSCA (Zhang et al. 2019), (option–reml) a C++ software that contains efficient functions for data management and estimation of the model parameters using Restricted Maximum Likelihood (REML). We repeated the analyses after rank inverse normalization of the brain measurements to investigate whether morphometricity estimates were impacted by non‐normal distributions of brain measurement. We extended the LMM to test whether the morphometricity of different processing explains the same trait variance or whether each captures a different or complementary proportion of trait variance (Appendix S2). As surface‐based processing does not provide cerebellum measurements, we tested whether the complementary proportion of trait variance comes from the cerebellum. We performed sensitivity analyses that focused on FSLVBM (without cerebellum measurements) and FreeSurfer. Lastly, we applied LMM with multiple random effects to decompose the morphometricity into the (conditional) contributions of the cortical, subcortical, and cerebellar (when available) measurements (Appendix S2).

### 2.10 Brain‐Wide Association Study

All analyses were conducted on the same participants, controlling for site effects and neuroimaging confounders.

#### 2.10.1 Family Wise Error Rate

We evaluated, for each processing, the false positive rate (Family Wise Error Rate; FWER) when using Bonferroni correction. Bonferroni's correction is a straightforward method that controls for FWER by setting a corrected significance threshold at alpha/nTests (nTests representing the number of brain measurements here). We expect Bonferroni's correction to be overly conservative (FWER < 5%) as it assumes independence among the tests, which is not the case here as brain measurements are correlated. We expect FWER to differ between processing, influenced by the amount of correlation between brain measurements and the non‐normal distribution of vertex/voxel‐wise measurements. On the other hand, we sought to confirm that non‐normal distributions of brain measurements can influence the false positive rate.

To estimate the FWER, we simulated 1000 normally distributed random traits (i.e., not associated with brain measurements). Then, we tested the association between each brain measurement and these traits, controlling for standard covariates that have been recommended for neuroimaging analyses of the UK Biobank: age, sex, total brain volume, gray‐matter density, head motion during resting stage fMRI, time since first scan, scanner brain position, as well as body‐size covariates (BMI, Waist and Hip circumference). For each processing, we calculated the FWER as the proportion of simulated traits yielding at least one significant association (hence false positive voxel/vertex) after Bonferroni correction. We used qqplots to contrast the distributions of *p* values obtained for each processing to that of a null distribution. To evaluate the effect of non‐normal brain measurements on the FWER, we repeated the analyses after rank inverse normalization of the brain measurements.

We iteratively tested the association (*bi*) between the simulated trait y and the th voxel/vertex‐wise measurement (*bi*) using a Generalized Linear Model–linear option in OSCA (Zhang et al. 2019).

(2) GLM:y=biXi+covariates+e

#### 2.10.2 Optimal Significance Threshold

To perform a fair comparison of the processing at the same level of false positives, we estimated the optimal significance threshold for each processing, which corresponds to FWER = 5%. To estimate this threshold, we extracted the minimal *p* value per simulated trait (1000 *p* values in total) and set our new threshold to the 5th percentile. Indeed, this approach identifies the largest *p* value significance threshold that ensures a rate of false positive (FWER) of 5%.

#### 2.10.3 BWAS on UK Biobank Traits of Interest

We investigated which gray‐matter measurements are associated with our UK Biobank traits of interest across the different processings by performing brain‐wide association studies. We controlled for all covariates (demographics, body size, and neuroimaging covariates) in the analyses. We included the same neuroimaging covariates as for the estimation of morphometricity (see Section 2, Table 1, covariates section).

**TABLE 1:** Phenotypes of interest in the UK Biobank and confounders.

Phenotype	Definition and explanations	Data field ID
Fertility and sexual behaviors: These traits have been widely studied in biology and socials science, and are linked to feeling, behavior and well‐being (Grinde 2021). Some sexual behaviors have been previously linked to gray‐matter structure. For example, previous studies have demonstrated that compulsive sexual behavior disorder is associated with reduced gray matter volume (Schmidt et al. 2017).		
Age first had sex	Mean = 19 years old (SD = 4.6)	2139
Number of children	Number of children fathered by men. Mean = 1.8 (SD = 1.3)	2405
	Number of live births reported by women. Mean = 1.7 (SD = 1.2)	2734
Socio‐economic variables Some previous study exhibited a link between socio‐economic status and cognitive skills (Thanaraju et al. 2024; Draps et al. 2020)		
Age when completed full time education	Mean = 17 years old (SD = 2.4)	845
English indices of multiple deprivation of the area of residence	Measure of socio‐economic environment based on the place of residence (based on seven distinct domains for England: Income, Employment, Health, Education Skills, Living Environment, Barriers to Housing and Crime). Mean = 16 (SD = 12). This phenotype has previously exhibited large morphometricity estimate (Couvy‐Duchesne et al. 2020)	26,410
Score of general cognitive ability	We constructed using the lavaan package in R, based on different measured of cognitive ability: Verbal Numeric Reasoning, Trail Making, Matrix Pattern Completion, Tower Rearranging, Symbol Digit Substitution, Pairs Matching and Reaction Time. The variance explained by this new “g” factor is 35% of the variance contained in individual cognitive measures, which is consistent with previous reports (Fürtjes et al. 2023). Mean = −0.07 (SD = 0.37).	20016,2019,6350,20157, 6373,21004,23324, 20159,399,20023
Substance use and exposure A previous review demonstrated that gray matter alterations are consistently observed across various substance use disorders, highlighting their potential role in addiction severity (Pando‐Naude et al. 2021).		
Maternal smoking around birth	Large morphometricity previously reported (Couvy‐Duchesne et al. 2020). 27% participants answered yes	1787
Smoking	Number of cigarettes smoked based on current and past smoking. Score between 0 and 5. Mean = 2.3 (SD = 1.0)	1239 and 1249
Alcohol frequency	Ongoing frequency of drinking alcohol, excluding former drinkers and cannabis initiation. Score between 0 and 4. Mean = 2.8 (SD = 1.6)	20414
Clinically relevant traits We included these traits as we expect them to have some association with the gray matter. They correspond to some of the most common diseases or clinically relevant traits present in the UK Biobank, which is a sample of volunteers		
Bipolar disorder (type I or II) and major depression status	Self‐reported. Methods on how these fields were derived can be found in a previous paper (Smith et al. 2013). Score between 0 and 4. Mean = 0.9 (SD = 1.6)	20126
Multisite chronic pain	Score from 0 to 8 as the sum of body sites at which chronic pain (for at least 3 months) was reported. Mean = 1.6 (SD = 0.92)	6159,3404,3414,3571,3741,3773,3799
Recent restlessness	Over the last two weeks, how often have they been so restless that it is hard to still. Multiple categories phenotype: 6% answered “several days”, 0.9% answered “more than half the days” and 0.6% “Nearly every day”	20516
Sleeplessness or insomnia	Self‐reported, and present in the past 4 weeks before the MRI. Multiple categories phenotype: 46% reported “Sometimes” and 31% “Usually”	1200
Diabetes	Type I or II or both? Self‐reported lifetime diagnosis. 5% reported being diabetic	2443
Tinnitus	Previous studies have reported structural brain abnormalities associated with tinnitus (Smith et al. 2013). Score between 0 and 5 (mean = 1.9, SD = 1.4)	4803
Stroke	Constructed from the date of the earliest reported stroke, either self‐reported or hospital‐reported. Diagnoses included both prior to and following MRI. 1.6% reported a stroke	42006
High blood pressure	Constructed from age when high blood pressure was diagnosed. Diagnoses included both prior to and following MRI. 28% of participants reported high blood pressure	2966
Parkinson's disease	Either self‐reported or hospital‐reported. *N* = 93 cases. 51% were diagnosed before the imaging visit (2.4 years before on average [min = 25 years before, max = 6.0 years after])	42032
Alzheimer's disease	Hospital‐reported. *N* = 32 cases; 29 were diagnosed after the imaging visit (2.6 years after on average [min = 2.4 years before, max = 5.8 years after])	42020

Known or hypothesized confounding covariates (Alfaro‐Almagro et al. 2021) We incorporated imaging confounders that have previously demonstrated an impact on the results of structural brain analysis (Alfaro‐Almagro et al. 2021)		
UK Biobank assessment center	There might be some differences in how radiographers proceed, or in the population leaving around the center. There might also be variability in equipment despite all center using same machines and protocol	54
Head positioning in the MRI scanner (X, Y and Z)	It has been shown that the head positioning was correlated with several quality‐check metrics (Kochanek et al. 2011)	25756,25757 25758
Inverted signal‐to‐noise ratio	Measure of contrast/quality of the raw image	
Mean rs‐fMRI head motion	Averaged across space and time points. Measured as part of rs‐fMRI acquisition and used as proxy of motion during T1w and T2 FLAIR acquisition. Microscopic head motion has been shown to be stable for an individual and associated with genetic factors (Couvy‐Duchesne et al. 2014)	25741
Discrepancy between T1 brain image and standard‐space brain template	linear alignment to the standard MNI152 brain template (Jenkinson et al. 2002)	25731
Volume of the brain	Gray + white matter volumes, using FMRIB Software Library	25009
Intensity scaling for T1	As a few minor protocol parameter changes have been made at the imaging sites for a small subset of volunteers	25925
Time since first MRI	For each center, using the date of assessment center for the neuroimaging visit	53
Confounders which exhibited large mophometricity in a previous study Standard confounders exhibiting large morphometricity (Couvy‐Duchesne et al. 2020), which may also be traits of interest in other studies (e.g., brain age prediction).		
Age	Self‐reported. Mean = 64 years (min = 44 years, max = 82 years, SD = 7.7)	34
Sex	53% women	31
Body mass index	Measured by a nurse as part of the imaging visit. Mean = 26 (SD = 4.4)	21001
Waist circumference	Manually measured by a nurse as part of the imaging visit. Mean = 88 (SD = 12.6)	49
Hip circumference	Manually measured by a nurse as part of the imaging visit. Mean = 100 (SD = 8.7)	50

We used several criteria to compare the results obtained using the different processing.

We first compared the association effect sizes by reporting the mean absolute z‐score ($\frac{|\beta |}{\text{Standard Error}}$) across all voxels/vertices for each trait and processing. We used the absolute *z*‐score to focus on the magnitude of associations regardless of sign and the scale of the vertex/voxel‐wise measurement.

Then, for each trait and each processing, we reported the number of significant vertices/voxels (using the optimal significance threshold that ensures comparable FWER = 5% for all processing). We also reported the number of significant clusters as well as their sizes. To identify clusters, we performed an iterative 3D cluster search using the *vcgKDTree* function implemented in the R‐library Rvcg. At each step, the algorithm considered the 10 nearest neighbors from the set of significant vertices/voxels and included them in the cluster if they were also significant. The algorithm stops when all significant vertices have been attributed to a cluster.

Lastly, we evaluated the number of clusters within each region of interest (ROI) and the number of ROIs containing at least one cluster for each processing. We evaluated robustness as the number of common brain regions (i.e., ROI that contain a significant cluster) identified by several processing.

### 2.11 Replicability of Findings

We evaluated the replicability of the results by performing similar analyses in the replication sample. Thus, we compared morphometricity estimates from the discovery and replication samples. In addition, we conducted a Brain‐Wide Association Study to report the proportion of replicating voxels and clusters. We used a significance threshold of 0.05/NSignifV, with NSignifV being the number of significant associations in the discovery sample (across all traits and processing), which corresponds to the total number of vertex/voxel‐wise associations we took to the replication sample.

### 2.12 Prediction

#### 2.12.1 Prediction From Significant Vertices/Voxels

We evaluated how much the significant vertices/voxels can together account for the traits of interest by evaluating their predictive power in the replication sample. Indeed, the significant associations can tag redundant signals, which do not necessarily translate into increased prediction (Couvy‐Duchesne et al. 2022). Typically, vertices/voxels from the same cluster often capture the same information, but this can also be the case between distant vertices/voxels that are correlated. We selected the most significant vertex/voxel in each cluster and constructed a linear predictor using association weights *b*_*i*_, estimated in the BWAS (Equation 2). We used OSCA (−score option) to calculate a score for each individual of the replication sample. We evaluated the prediction accuracy (*R* ^2^) of these scores in the replication sample using a linear model that controlled for all site effects and neuroimaging covariates and tested its significance with a likelihood‐ratio test (nested models, between a full model with predictor and covariates versus a reduced model with only covariates).

We finally compared the results obtained with those using all significant vertices/voxels. For that, we compute the prediction accuracy using the same model but including all significant voxels instead of only the top ones. This comparison allows us to conclude about the redundancy of the signal captured by all brain measurements.

#### 2.12.2 Prediction From the Whole Gray Matter

Machine learning approaches aim to build performant brain‐based predictors that are not limited to statistically significant regions. We built Best Linear Unbiased (BLU) Predictors to evaluate which processing is best suited to machine learning and prediction analyses. BLU Predictors have been widely used in animal and human genetics and have previously been applied to vertex‐wise data of the UK Biobank, where they showed similar to superior performances compared to LASSO predictors (Couvy‐Duchesne et al. 2020). Unlike many machine learning models, BLU Predictors do not require cross‐validation as they leverage maximum likelihood estimation for parameter estimation. BLU Predictors are efficiently implemented in OSCA and easily scale up to large samples. We trained BLU Predictors in the UK Biobank discovery sample and evaluated them in the replication sample, controlling for all covariates. As previously, we reported prediction accuracy as an *R* ^2^ (hence comparable to the morphometricity estimates). We further reported the fraction of morphometricity that BLU Predictors can predict.

## 3 Results

### 3.1 Vertices and Voxels Distribution and Correlations

We investigated the distribution of vertices and voxel‐wise measurements from each brain MRI processing. We found that some processing exhibited positive kurtosis (> 3, Table 2). In particular, FreeSurfer‐based processing exhibited larger median kurtosis (4.1 for FreeSurfer Thickness and 4.9 for FreeSurfer All Modalities), suggesting that many vertices/voxels have heavier tails than those expected in a normal distribution, leading to increased occurrences of extreme values. Skewness levels suggest that the vertex/voxels distributions were largely symmetrical, except for FreeSurfer processing where skewness indicated the presence of right tails in vertex‐wise distributions. CAT12 Surface stood out as the only processing with negative (albeit moderate) median skewness (Table 2), implying that, unlike in other processing, its vertices measurements have longer left tails.

**TABLE 2:** Data description for each processing.

Processing	No. of measurements	s	Median skewness	Median sum*R* ^2^/nb brain measurements (×10^−4^)
FSLVBM	181,544	3.0	0.36	2.3
FSLANAT	184,637	3.1	0.35	2.6
CAT12 volume	192,483	3.3	0.24	7.3
CAT12 surface	299,881	3.5	−0.076	49
FreeSurfer thickness	299,881	4.1	0.79	4.6
FreeSurfer all modalities	654,002	4.9	1.1	1.6

*Note:* First column summarizes the number of voxels/vertices brain measurements from the 6 brain MRI processing pipelines. Median kurtosis (resp. skewness) quantifies the non‐normality of the gray‐matter measurements. Both were computed with the “moments” package in R. The median sum*R* ^2^ quantifies the amount of correlation in the structural connectome. We divided it by the number of brain measurements to obtain a measure comparable across processing that has a different number of voxels/vertices.

We calculated the sum*R* ^2^ to quantify the overall amount of correlation in each structural connectome. CAT12 processing methods, and particularly CAT12 Surface, exhibited large median sum*R* ^2^ (Table 2), suggesting that their vertices/voxels have a higher degree of correlation. We investigated whether this large median sum*R* ^2^ came from the non‐normal distribution by re‐estimating it after adjustment for covariates and rank‐inverse normalization: we found that, it was robust to non‐normal distributions (before and after adjustment *r* = 0.999), suggesting that this amount of correlation is inherently linked to the processing method itself.

Next, we investigated if highly correlated regions (with high sum*R* ^2^) were the same across processing. It was the case for the three volume‐based processing methods, which exhibited similar mean sum*R* ^2^ per ROI, with a correlation of *r* = 0.97 between FSLVBM and FSLANAT and *R* ^2^ = 0.70 between FSLANAT or FSLVBM and CAT12 Volume. Both cortical thickness processing methods exhibited a correlation of 0.57. However, the ROI with high sum*R* ^2^ differed between volume and surface‐based processing. For example, the correlation of mean sum*R* ^2^ across ROI between FreeSurfer and FSLVBM was 0.47. This suggests that the pattern of correlation between brain measurements is highly dependent on the representation used (volume or surface). Detailed mean sum*R* ^2^ per ROI can be found in Table S1.

Overall, some processing exhibited non‐normal distributions, and we observed varying levels of correlation among voxels/vertices that can both impact further associations analysis.

### 3.2 Morphometricity of Putative Confounders

**FIGURE 2:**
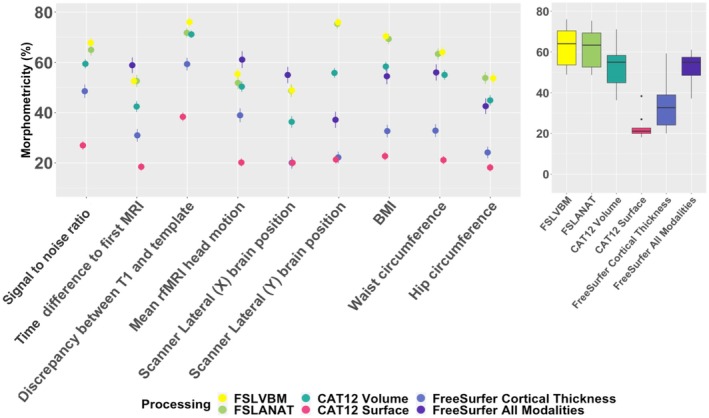
Morphometricity of possible confounders, imaging and body size. The left panel depicts the morphometricity of each possible confounder (after controlling for age and sex). The different colors correspond to the different processings, and the bars represent the 95% confidence intervals. The boxplot (right panel) summarizes the distribution of morphometricity estimates across all possible confounders.

We estimated the morphometricity of several traits known (e.g., head motion) or hypothesized (e.g., time since first scan) to have an effect on brain images, which would quantify how much the possible confounders may contaminate each image processing (Alfaro‐Almagro et al. 2021). We found that all the considered confounders (either imaging or body size) exhibited a large morphometricity (*R* ^2^ in 20%–80%) (Figure 2), which implies they are associated with one or several brain regions. Interestingly, cortical thickness measurements (from FS or CAT12) appeared the least associated with possible confounders (median morphometricity across traits 32% ‐FreeSurfer‐ and 21% ‐CAT12, Figure 2). In comparison, FSL based processing (FSLANAT or FSLVBM) showed the largest associations with the possible confounders (medians morphometricity 63%–64%) (Figure 2).

Next, we used LMM with multiple random effects to jointly estimate the variance accounted for by cortical, subcortical, and cerebellar measurements (when available). For FreeSurfer all modalities, we further separated cortical thickness and surface area. Of note, FreeSurfer Thickness and CAT12 Surface only included measurements of cortical thickness and were not included in these additional analyses. We found that across all processings, the different brain parts (cortical, subcortical, and cerebellar) were all associated with confounders (Figure S4), which suggests they can cause widespread false positives.

### 3.3 Morphometricity of Traits of Interest

**FIGURE 3:**
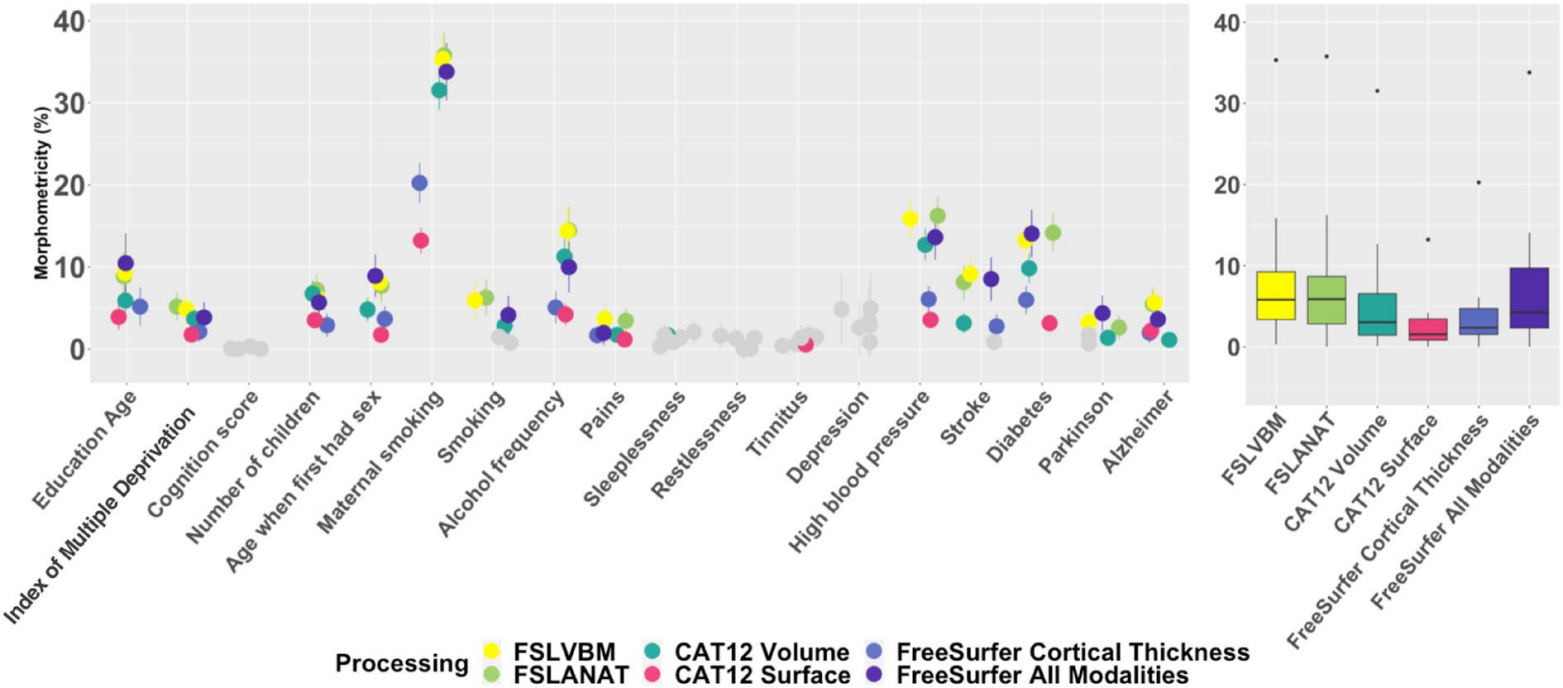
Morphometricity of traits of interest, controlling for all covariates. The left panel depicts the morphometricity of each trait of interest when controlling for all covariates (see Section 2, Table 1, confounding covariates and confounders sections). The different colors correspond to the different processing, and the bars represent the 95% confidence intervals. We represented in gray the morphometricity estimates that were not significantly different from 0 (after multiple testing correction *p* < 0.05 (18 * 6)). The boxplot (right panel) summarizes the distribution of morphometricity estimates across all traits of interest for each processing.

When controlling for all confounders (age, sex, and key neuroimaging covariates), both FSL processing accounted for the largest proportion of variance (median = 5.8%) followed by FreeSurfer All modalities (median = 4.2%) and CAT12 Volume (median = 3.0%). CAT12 Surface yielded the smallest proportion of variance explained (median = 1.5%). Moreover, we detected significant morphometricity (after Bonferroni correction, i.e., *p* < 0.05/(18 * 6)) for most traits of interest (except for restlessness, depression score, and general cognition score, depicted in gray on Figure 3). We observed that morphometricity varied depending on the trait. Maternal smoking around birth exhibited the largest morphometricity (13%; 36%) followed by diabetes (5%; 15%), high blood pressure (5%; 14%), and alcohol frequency (4%; 14%).

When modeling brain parts and measurement types in specific random effects, we found (Figure S5) that, overall, all broad regions contribute to the detected morphometricity. For FSLVBM, the variance in traits of interest was mostly explained by the cortical and subcortical measurements, even though cerebellar measurement also captured some significant information, for maternal smoking, for instance. For all traits with a significant morphometricity, we confirmed that cortical and subcortical measurements contributed to the association. We also found that the cerebellum was significantly associated with maternal smoking and diabetes. Results remained consistent after we rank‐normalized voxels/vertices for each processing, which suggests morphometricity estimates are robust to the non‐normal distributions present in the data (Figure S6).

### 3.4 Consistent Morphometricity Estimates in the Replication Sample

We observed mostly comparable estimates of morphometricity in the replication sample (Figure S7), suggesting that morphometricity estimates are replicable. Of note, morphometricity was smaller for the “time difference to first MRI” for all processing. This is likely due to the fact that the scanners are more recent in the replication sample (average scanner age of 3 years in the discovery vs. 0.9 years in the replication dataset), leading to less variability in the time since the first MRI. In addition, we observed larger estimates of morphometricity from FreeSurfer processing in the replication analysis (Figure S7), particularly for traits such as Mean motion during rfMRI, SNR, Stroke, Parkinson, or Maternal smoking. This could be partly explained by the smaller numbers in the replication sample (i.e., larger standard errors), although we cannot rule out differences in scanner (e.g., software) or acquisition that could impact the output from FreeSurfer.

### 3.5 Morphometricity Shared Between Processings vs. Unique to Each Processing

**FIGURE 4:**
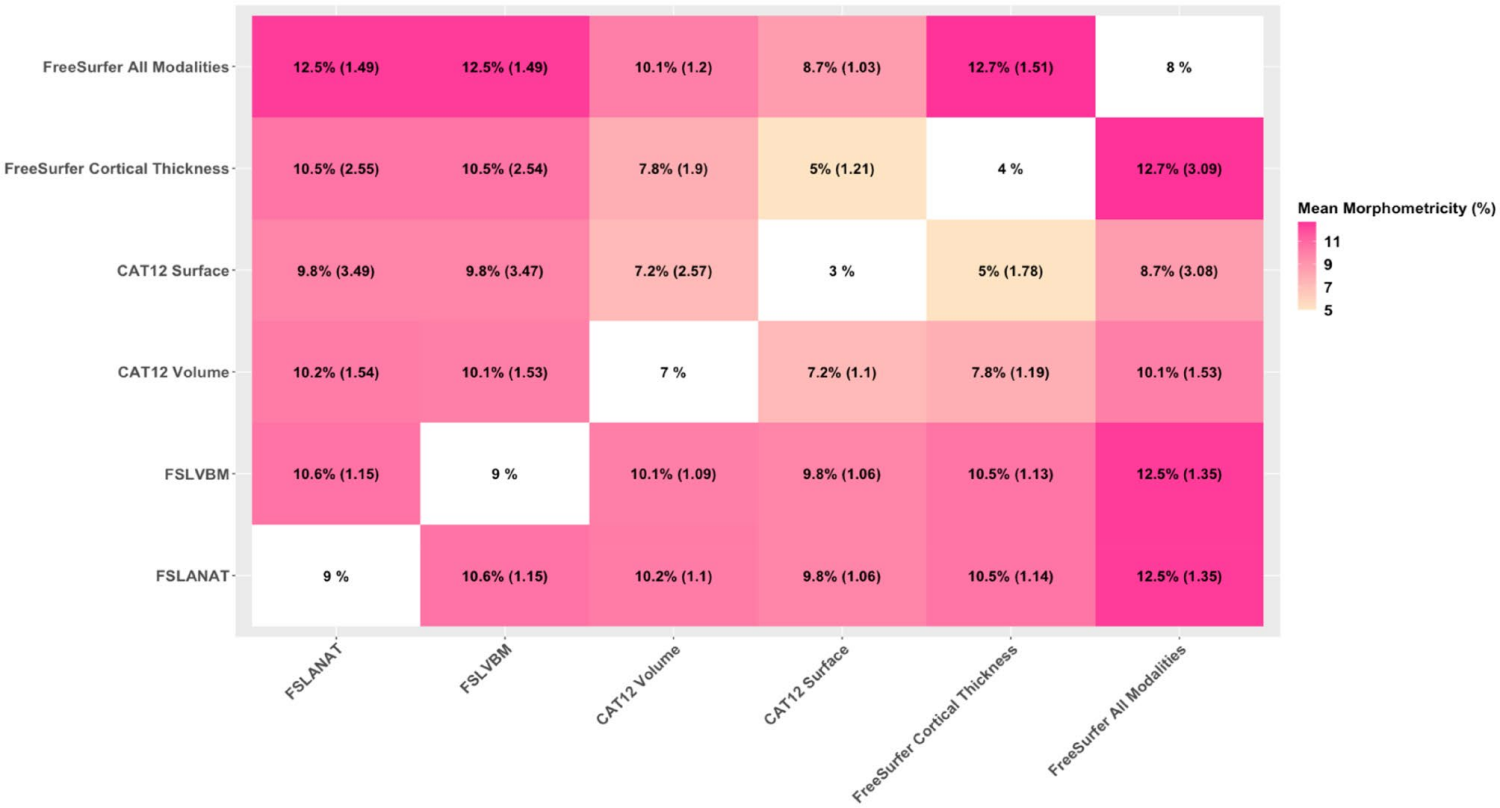
Heatmap of morphometricity increase when fitting two gray‐matter representations in the model. The heatmap shows the average percentage of variance explained (morphometricity) across all traits of interest when combining two processing methods. The rate of increase is shown in parentheses, and diagonal values represent the mean morphometricity of each processing method alone. Rows indicate the reference processing and columns the added processing. For example, the second row of the first column corresponds to the result of FSLANAT added to FreeSurfer Cortical Thickness, resulting in an average morphometricity of 10.5%, 2.55 times higher than FreeSurfer Cortical Thickness alone (average morphometricity 4%, second row, 5th column). Symmetrically (6th row, 5th column), adding FreeSurfer Cortical Thickness to FSLANAT only slightly increases morphometricity (×1.14) as FSLANAT explains 9% of variance by itself (6th row, first column).

We investigated whether the processings captured a different and/or complementary proportion of the trait variance. We only focused on traits exhibiting significant morphometricity (i.e., we excluded Cognition, Depression, and Restlessness). We found that fitting gray‐matter measurements from two processings resulted in an overall increase in the proportion of explained variance (Figure 4), which suggests that each gray‐matter representation captures a unique proportion of trait variance that is missed by other processing.

For example, when combining the two top processing in terms of morphometricity (FSLVBM and FreeSurfer All Modalities), the trait variance accounted for grew to 12.5%, on average, across all traits (vs. 10.1% for FSLVBM and CAT12 Volume or 8.7% for FS All Modalities and CAT12 Surface). This represents an increase in variance explained, with a ratio of 1.49 and 1.35 (compared to Freesurfer and FSLVBM, respectively). This suggests that both processes capture a unique fraction of the trait variance (35%–49% of the signal) in addition to the variance they both capture (51% to 65% of the morphometricity).

As expected, CAT12 surface captured less morphometricity than its competitors (average 2%, Figure 4), so the gain of adding an additional set of brain measurements was maximal (range 2.59–4.43). We also noted that FSLANAT and FSLVBM detected mainly the same morphometricity, with only about 10% of unique signal (Figure 4).

We confirmed that the improvement was significant for each trait (Figure S8). For example, for “Maternal smoking” FreeSurfer All modalities explained 33.8% of the variance, and FSLVBM 35.3%, but together they accounted for 46%. As FreeSurfer does not measure the cerebellum region, we wondered whether FSLVBM's unique signal is due to the cerebellum. Therefore, we performed a similar analysis including only FSLVBM (without cerebellar measurements) and FreeSurfer All Modalities. Overall, it did not result in any significant difference compared to the volume‐based processing unique signal. Therefore, the cerebellum does not fully explain these discrepancies.

### 3.6 False Positive Rate of Vertex/Voxel Wise Association (BWAS)

We previously showed that processing exhibited non‐normal distributions of the gray matter, implying that data have higher probabilities of extreme values (high or low) than would be expected in a normal distribution, which may impact association testing.

**FIGURE 5:**
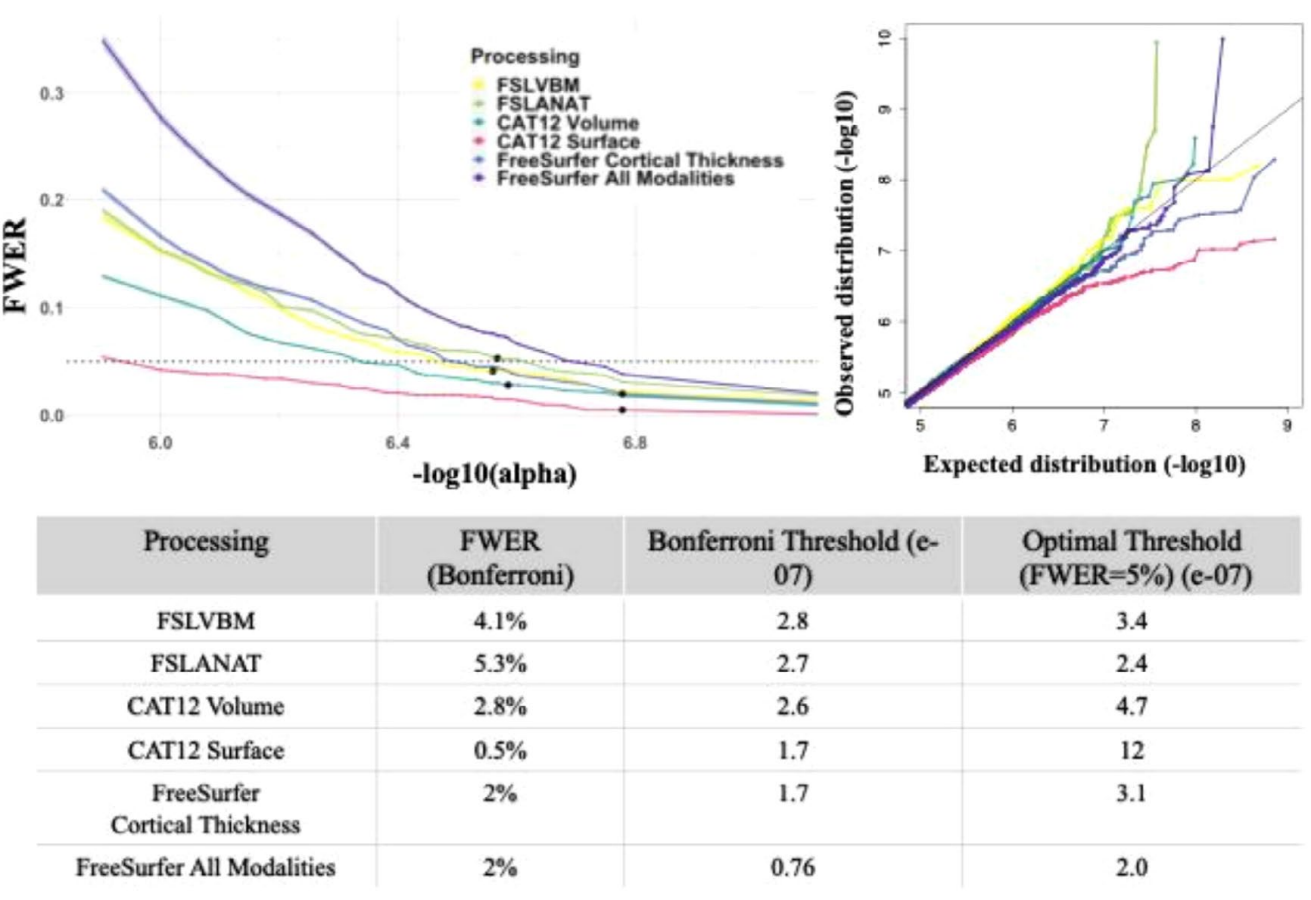
Family Wise Error Rate of BWAS, Q‐Qplot of minimal *p* values and optimal significant thresholds. The top left panel depicts the Family‐Wise Error Rate (FWER) at different significance thresholds. The *x*‐axis shows the significance thresholds in the log scale, to improve readability. The horizontal dashed line represents an FWER of 0.05. The black dots indicate the Bonferroni's significance threshold of each gray‐matter processing. The shaded bands correspond to the binomial proportion confidence intervals at 95%, which are narrow < 0.02 due to the high number (1000) of simulated traits. The top right panel depicts the QQ‐Plot of the minimal *p* values for each vertex/voxel across the 1000 simulated traits. Minimal *p* values are represented in log10 scale. The bottom table reports the FWER with Bonferroni's correction (i.e., the black dots on the top left plot) as well as the optimal significant threshold when ensuring FWER = 5%. For comparison, we also reported the Bonferroni's significance threshold (middle column).

We evaluated how stringent Bonferroni's correction was for each processing by estimating the Family wise error rate (FWER) < 5%, under the null hypothesis. We expected to find FWER lower than 5% for all processing, especially those that exhibited a large amount of correlation between voxels/vertices (median sum*R* ^2^, Table 2), as it induces non‐independence of the test statistics. Our findings (Figure 5, top‐left panel) indicated that Bonferroni's correction effectively ensured a FWER < 5% except for FSLANAT (FWER = 5.3%, Figure 5), suggesting that using this processing may result in a false positive rate above 5%. For all other processing, Bonferroni's correction was overly conservative (FWER largely below 5%, Figure 5, bottom table): FreeSurfer All Modalities exhibited a FWER of 2%, FSLVBM of 4%, and CAT12 Volume of 3%. Bonferroni's correction was particularly stringent for CAT12 Surface, as indicated by an FWER of 0.5%, which is consistent with the fact that it is the processing with the larger amount of correlation between vertices (Table 2).

#### 3.6.1 Optimal Significance Threshold

To compare processing to a set FWER level, we derived optimal significance thresholds (intersection of the colored lines and dashed lines/bottom table, Figure 5) that correspond to FWER = 5%, therefore less stringent than Bonferroni's correction for most processing. CAT12 Surface had a new threshold of 1.2e‐06 compared to 3.1e‐07 for FS Thickness and 2.0e‐07 for FS All modalities. For volume‐based processing, the optimal threshold for FSLVBM was equal to 3.4e‐07, compared to 2.4e‐07 for FSLANAT and 4.7e‐07 for CAT12 Volume.

On the other hand, to evaluate the impact of distribution on the false positive rate, we looked at the distribution of the minimal *p* value per voxel/vertex across the 1000 random traits to focus on the top associations that are most likely to reach significance for each processing. The QQ‐plot of FSLANAT (green, right panel, Figure 5) showed an inflation for the top percentiles of the distribution, meaning that a couple of voxels exhibited a more significant *p* value distribution than expected by chance (Figure S10, left column) and are more likely to exhibit a significant association. After applying a rank‐inverse normal transform to the brain measurements (after covariate adjustments), the Q‐Q plot of FSLANAT (Figure S10, right column, green) aligned more closely with the expected distribution, confirming the inflation was due to non‐normal distributions.

Additionally, we observed a slight QQ‐plot deflation for FS Thickness, FS All Modalities, and a more important one for CAT12 Surface. The deflation of *p* values of FreeSurfer Cortical Thickness was reduced after applying rank‐inverse normal transform to the brain measurements. On the other hand, the deflation of CAT12 Surface was attenuated but remained, which suggests CAT12 Surface may suffer from a low type II error. We therefore can expect CAT12 Surface to be conservative and detect fewer significant associations.

### 3.7 BWAS of Traits of Interest

We tested the association between our voxels/vertices and the UK Biobank traits of interest, controlling for all covariates. We did not single out results for the FreeSurfer Cortical Thickness processing, as all its vertices are included in FreeSurfer All Modalities.

#### 3.7.1 Effect Sizes of Vertex/Voxel‐Wise Associations

The statistical power of BWAS depends in part on the effect sizes between traits and vertex/voxel‐wise measurements. Thus, we investigated if different processing methods yielded differences in effect sizes, measured by the median absolute *z* score across all voxels/vertices.

**FIGURE 6:**
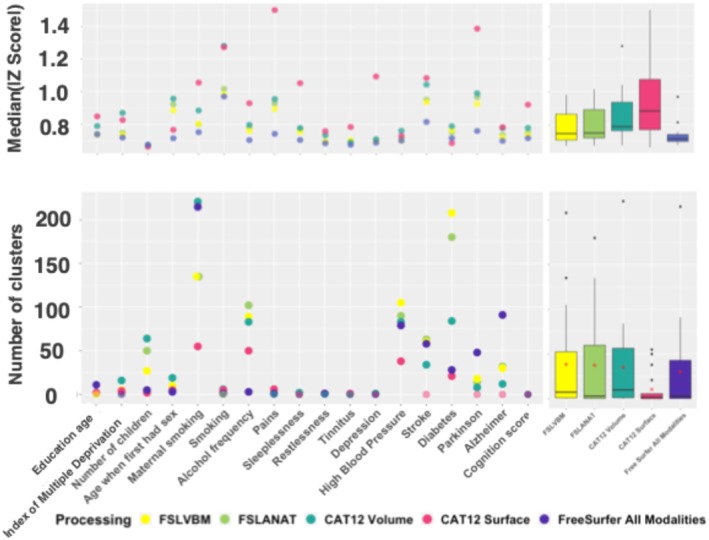
Median absolute *z*‐score across the brain and Number of clusters after correcting with optimal significance threshold across all traits and processings. The top‐left panel depicts the median absolute z‐score for all traits of interest. The top‐right boxplot displays the overall distribution of these scores. The bottom‐left panel depicts the number of significant clusters for all traits of interest. The bottom‐right boxplot displays the overall distribution of the number of clusters across all traits of interest for each processing. Red stars represent mean values.

CAT12 Surface exhibited the largest effect sizes with a median *z* score of 0.95 on average and a maximum of 1.5 (Figure 6, top panel), followed by CAT12 Volume (median = 0.85, max = 1.3), compared to FSL processing (FSLANAT median = 0.79, max = 0.98, similar results for FSLVBM) and FreeSurfer (median = 0.73, max = 0.97).

Traits with larger morphometricity (maternal smoking, diabetes, High blood pressure) also displayed larger average z‐scores, implying larger associations in the brain, thus greater statistical power to identify significant vertices/voxels than for the other traits.

#### 3.7.2 Number of Significant Clusters After Multiple Testing Correction

We used the optimal significance threshold (corresponding to FWER = 5%) in the following analysis to allow for a fair comparison of the processing methods.

We found that (Figure 6, bottom panel) CAT12 Volume and FSLVBM yielded the highest number of clusters across all traits (median = 10 for CAT12 Volume and 7.5 for FSLVBM). In comparison, we identified a median of 3 clusters for both FS All Modalities and FSLANAT and 2 for CAT12 Surface. At a trait level, the number of significant clusters varied widely between processing. For example, we identified > 200 clusters associated with “Maternal smoking” using FreeSurfer (*N* = 221) and CAT12 Volume (*N* = 215), 135 clusters using FSL and 55 using CAT12 Surface (Figure 6, bottom left panel). There was a correlation (*r* = 0.89) between the number of associated clusters and the morphometricity, indicating that morphometricity is a good predictor of discoverability, although other factors also contribute (e.g., effect sizes and number of tests).

To explore these clusters in more detail, we examined their size and distribution. As expected, processing with large correlation among their voxels/vertices had larger clusters and a smaller proportion of clusters made of a single voxel/vertex: 9% of the CAT12 Surface clusters contained a single vertex (and median size of clusters = 11), versus 45% of the CAT12 Volume clusters (median size of clusters = 2) and 60% for all three other processings (with a median size of clusters equal to 1 for all three of them).

#### 3.7.3 ROIs Robustly Associated Across Different Processings

We also assessed the robustness of associations across different processing, meaning their ability to detect clusters in the same ROIs.

We found that, for all three volume‐based processing methods, the significant clusters implicated more ROIs (median = 14 for FSLANAT and 10 for FSLVBM and CAT12 Volume, compared to 7 for FreeSurfer and 4 for CAT12 Surface) (Figure S13). Especially, for maternal smoking (67 ROIs for CAT12 Volume, 55 for FSLVBM and 45 for FSLANAT) and diabetes (46 and 47 for FSL processing, and 37 for CAT12 Volume), which is consistent with the large number of significant clusters associated with this trait.

We focused on all traits that identified significant clusters across all processing methods (Figure S14). We observed the strongest agreement between both FSL processing (although never perfect) and slightly lower agreement between FSL and CAT12 volume processing. The overlap of significant ROI was even lower between FSLVBM and Freesurfer. CAT12 Surface had poor agreement with all other processing. This robustness pattern was similar across all traits.

For example, for “Maternal smoking” (Figure S14), 32 ROIs were robustly implicated by different volume‐based processing (for a total of 45–67 ROIs detected, hence rates of 47%–71%). In comparison, only 12 of the ROIs were detected by FSLVBM and FreeSurfer (out of 27 detected by FreeSurfer), and only 5 were common to FSLVBM and CAT12 Surface (out of 26 detected by CAT12 Surface).

### 3.8 Brain Based Prediction

**FIGURE 7:**
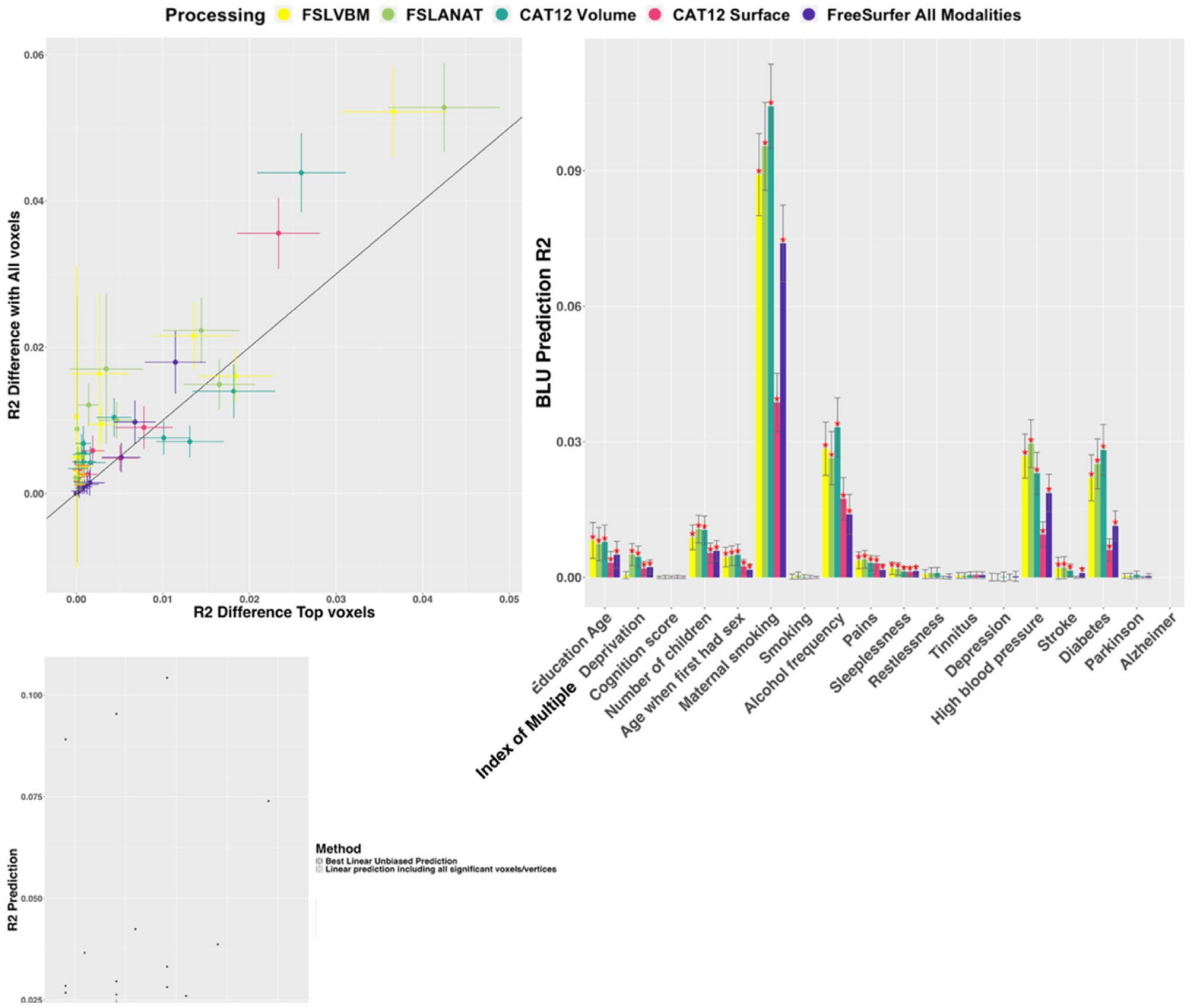
Linear prediction and best linear unbiased prediction. The top left panel compares the prediction accuracy including only the top‐voxels/vertices per cluster (*x*‐axis) versus all significant voxels/vertices (*y*‐axis). The vertical and horizontal bars show the 95% confidence intervals in the two samples. The top right panel depicts the prediction accuracy R2 using the Best Linear Unbiased Prediction method. Red stars indicate significant log‐likelihood ratio test after Bonferroni correction (*p* < 0.05/(6 * 29)) and vertical bars represent the confidence intervals. The bottom panel recapitulates all three volume‐based processing and top phenotypes predicted, the morphometricity estimates (in gray), and the BLU prediction as well as the fraction of morphometricity predicted in brackets.

We compared prediction accuracy achieved in the UK Biobank replication sample from significant voxels/vertices (after optimal significance threshold correction, Figure 7, top left panel). We contrasted the prediction achieved from the top vertex/voxel per cluster to that coming from all significant vertices/voxels. We observed (Figure 7, top left panel) that, for several traits and processings, the prediction was greater when including all significant voxels/vertices, which suggests that some clusters contain additional signal that is not fully captured by the most significant voxel/vertex. We expect more significant clusters to translate into greater prediction accuracy, unless several clusters tag the same information. Of note, “Alzheimer's disease” could not be predicted as there were no participants with records of Alzheimer's disease in the replication sample.

The top voxels from volume‐based processing yielded greater predictions than the vertices from surface‐based processing. In particular, for traits such as “Maternal smoking,” “Alcohol frequency,” “High blood pressure” (Figure S15, left panel), where prediction *R* ^2^ ranged between [0.018–0.029] for volume‐based methods versus *R* ^2^ in [0.0056–0.014] for both surface‐based ones (non‐overlapping confidence intervals). Of note, using Bonferroni correction (instead of the optimal one) had little incidence on the prediction achieved from significant vertices/voxels (Figure S15, right panel).

Next, we evaluated the prediction accuracy of Best Linear Unbiased Predictors, which capture signals across the whole brain and are not limited to vertices/voxels reaching significance. As expected, BLUP yielded improved prediction compared to only using significant vertices/voxels (Figure 7, bottom panel). As previously noted, the three volume‐based processing methods exhibited better predictions than surface‐based processing (*R* ^2^ = 0.01 on average vs. *R* ^2^ = 0.007 for both surface‐based processing). The prediction was the largest (and significant across all processing) for “Education age,” “Number of children,” “Maternal smoking,” “Alcohol frequency,” “Sleeplessness,” “High blood pressure,” and “Diabetes” (Figure 7, top‐right panel). For these traits, we reported the fraction of morphometricity predicted using each processing method (Figure S16). Overall, FreeSurfer resulted in a lower fraction of morphometricity predicted (median = 10%) compared to 17%–19% for all four other methods. Interestingly, we found that the proportion of predicted morphometricity varied from one trait to the next, which suggests that some traits are harder to predict. For example, 27% of the morphometricity of “Maternal smoking” could be predicted (median across processing), compared to 18% for both “High blood pressure” and “Diabetes,” and even lower for the other traits (Figure S16).

Finally, as we checked the prediction achieved by clusters of size 1 (Figure S17). For some processing and traits (number of children, age when first had sex, maternal smoking, alcohol frequency, high blood pressure, stroke, and diabetes), the prediction reached significance, suggesting that clusters of size one can sometimes tag true associations. However, the predictions remained low (e.g., *R* ^2^ = 0.008 for Maternal smoking [FSLVBM processing], in comparison with *R* ^2^ = 0.09 from all significant voxels). Of note, the clusters of size one identified by FreeSurfer did not significantly predict any traits (*R* ^2^ < 0.0006).

#### 3.8.1 Replication Rate of Significant Vertices/Voxels and Clusters

We investigated whether significant voxels detected in our main analysis replicated an independent UK Biobank dataset (replication sample). To control for multiple testing in the replication sample, we used Bonferroni corrected significance thresholds of 39,909 for the vertex‐wise level and cluster‐wise inference, which accounts for the total number of discoveries, across traits and processings.

Volume‐based processing all showed a good replication rate (35%–38% of voxels replicated, and 17%–19% clusters). CAT12 Surface displayed a lower replication rate at the vertex level (17%), but comparable at the cluster level (22%). This was due to many vertices from large clusters not replicating, although the core of the cluster reached significance. Moreover, FreeSurfer exhibited a higher replication rate at the vertex level (62%) but a low rate at the cluster level (10%). In fact, the vertex‐level replication rate was driven by “High blood pressure” trait: indeed 60% of FreeSurfer significant vertices were associated with “High blood pressure” (vs. 26%–27% for FSLANAT and FSLVBM processing, 12% for CAT12 Volume and 4% for CAT12 Surface). When excluding this trait, the vertex‐wise replication rate was reduced to 14% for FreeSurfer, while the cluster‐wise replication was reduced to 7%. Of note, the replication rate of FSLANAT and FSLVBM processing was also driven by associations with “High blood pressure” (16%–17% of replication rate on all other traits). Similarly, CAT12 Volume replication rate was driven by associations with “Diabetes” (representing 34% of the significant voxels), without which the replication rate reduced to 22%.

The low cluster‐level replication rate of FreeSurfer was mostly driven by the clusters of size one. Strikingly, FreeSurfer and FSL‐based processing had similar proportions of clusters of size one (60%–63%), but the replication rate of these clusters was 8.5% and 7.2% for both FSLANAT and FSLVBM, while it was only 2.1% for FreeSurfer. Of note, the clusters of size one identified with CAT12 Volume or CAT12 Surface also showed a 7% replication rate. When excluding clusters of size one, the cluster‐wise replication rate was 33%–35% for FSLANAT and FSLVBM, 29% for CAT12 Volume, and 23% for CAT12 Surface and FreeSurfer.

#### 3.8.2 Location and Robustness of Replicated Clusters in the Brain

**FIGURE 8:**
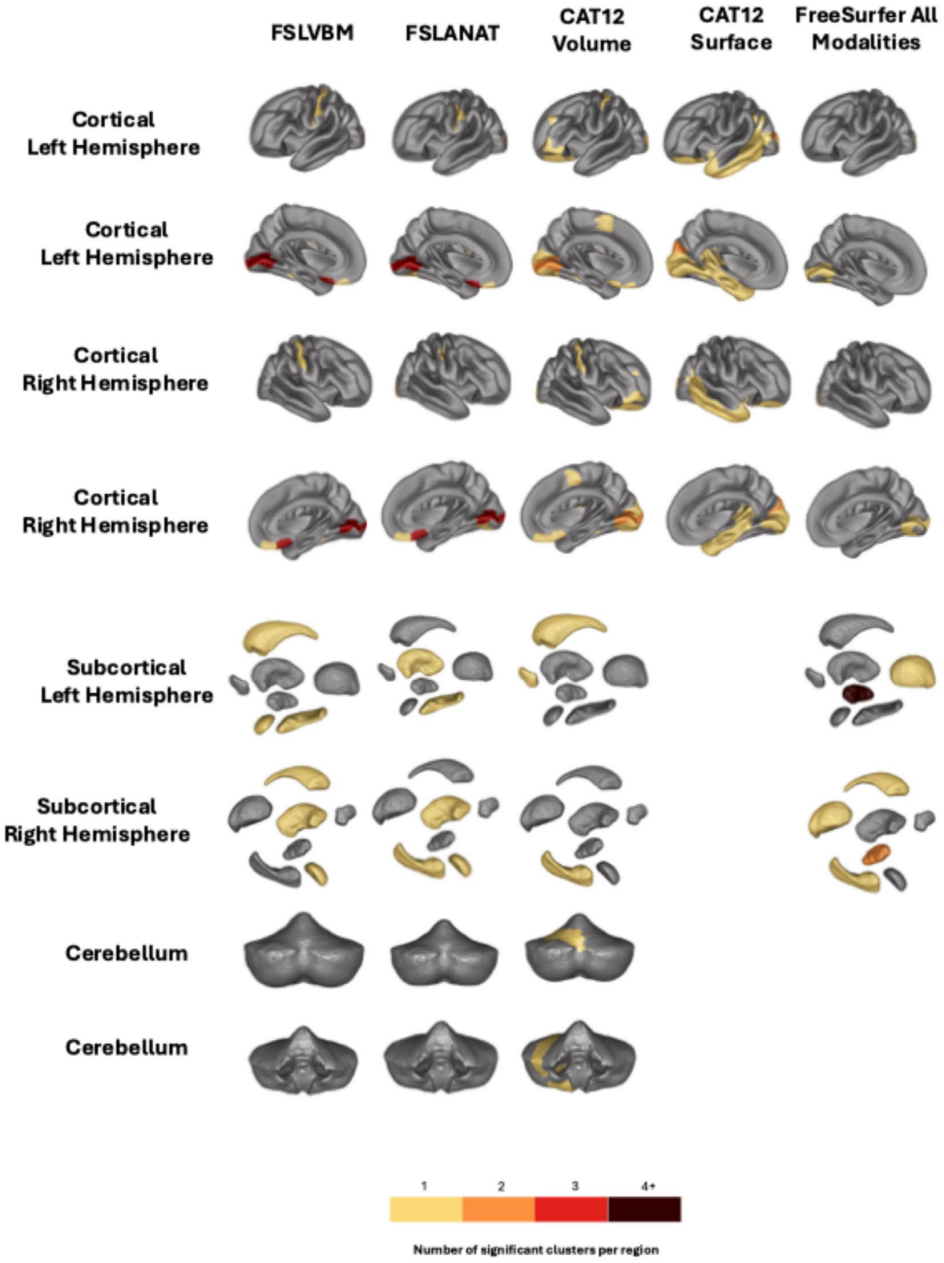
Location replicating cluster per region of interest for maternal smoking.

We checked whether the clusters were in the same ROIs across all processing (Figure 8), focusing on the ones that replicate, that is, where the strength of evidence is stronger. For “maternal smoking around birth,” 42 ROI replicated across all processing. All processing implicated the cortical region “Area_h0c1_ V1, _17, _Calcarine Sulcus.” In addition, the three volume‐based processing identified clusters in the ROIs Area_Fo1_ (OrbitoFrontal Cortex), Area_Fo2_ (OrbitoFrontal Cortex), CGL_ (Metathalamus), STN_ (Subthalamus), and Frontal‐to‐Temporal‐II_ (GapMap). Overall, 45% of ROIs were robustly identified by at least 2 processing.

For Diabetes, Left Thalamus as well as Right Crus_I, right_VI regions were robustly associated across all processing. Out of 37 ROIs identified in total, 49% ROIs were identified by at least two processing methods. “High blood pressure” did not exhibit ROIs identified by all processing (Figure S19), but 41% of the regions (out of 34 ROIs) were implicated by at least two processing methods. Finally, “Alcohol frequency” (Figure S20) had the lowest rate of clusters identified by at least two processing methods (4/29).

We were not able to define replicating clusters for Alzheimer's disease (there were no patients with Alzheimer's disease recorded in the replication sample). However, in the discovery cohort, all processing exhibited an association in the right hippocampus and the right amygdala.

## 4 Discussion

Using data from 39,655 UK Biobank participants, we compared five major processing pipelines for T1w brain MRI (Figure 1). We evaluated how the choice of processing influenced the total association (morphometricity) between gray matter and 29 traits of interest, the ability to detect associations in BWAS, and the performance of predictive models. Across all analyses, we accounted for standard covariates, site, and neuroimaging confounders that could impact results.

Diagnosis was analyzed as a trait of interest rather than a covariate, though its impact could be relevant in other studies. Since all participants were UK volunteers, we assumed minimal population bias, but this is worth noting in the discussion.

### 4.1 All Processing Contained Some Non‐Normal Brain Measurements

A first observation was that all processing contained some non‐normal brain measurements (Table 2), although it was more pronounced in FreeSurfer (higher kurtosis and positive skewness on average). These non‐normal distributions may influence some analyses that are sensitive to outliers or tails of distributions.

### 4.2 All Processing and Brain Parts Are Sensitive to Imaging and Body Size Confounders

Our results confirm that all considered image processings are sensitive to putative imaging confounders (Figure 2) (e.g., SNR, head position, head motion, and age of the magnet) and to body size measurements (e.g., BMI), as indicated by large morphometricity estimates (20%–80%). As for CAT12 Surface, its lower association with confounders (Figure 2) does not mean that it is less sensitive in light of the lowest morphometricity it detects overall. Moreover, we showed that putative confounders are associated across the brain and the different types of measurements (Figure S4) (cortical, subcortical, cerebellum, cortical thickness, and surface area). This suggests that imaging and body size measurements can create false positive associations in any brain part or gray matter measurement. Therefore, we recommend systematically controlling for these confounders in gray matter analyses, for example, by including them as covariates (in association studies or when evaluating predictors) and/or when selecting matched cases and controls.

### 4.3 FSLVBM, FSLANAT and FreeSurfer Maximize the Morphometricity, but Each Processing Captures a Unique Signal

Across 19 traits of interest (and controlling for all putative confounders), we found that the choice of processing impacted morphometricity estimates. For example, CAT12 Surface exhibited the lowest morphometricity across traits (Figure 3).

Some of the differences between CAT12 and FreeSurfer may be due to CAT12 using only T1w, while FreeSurfer relies on both T1w and T2 Flair. However, CAT12 Volume also showed a lower morphometricity compared to FSL volume‐based processing, which relied on the same T1w image, which points toward a software effect on the differences. Additionally, manual QC might yield different results by removing individuals in processing more susceptible to registration of segmentation issues. Yet, manual QC becomes impractical for large datasets, which calls for robust processing, or automated QC such as the one performed by the UK Biobank.

On the other hand, FSLVBM and FreeSurfer (all measurements) maximized the morphometricity (with estimates ~3× larger than those of CAT12 surface; range of morphometricity = [0%–13%]; Figures 3 and 4) in both the discovery and replication datasets. However, when combined, two processings resulted in a marked increase in morphometricity estimates, suggesting that each method captures a unique signal (Figure 4) that is absent in the other processing. For example, for Maternal smoking around birth, 75% of the signal FSLVBM and FreeSurfer All Modalities captured was unique, while 25% was shared. We confirmed that the cerebellum alone (not measured in FreeSurfer) could not explain the unique signal captured by FSLVBM. Our results shed new light on some of the lack of robustness to processing methods in neuroimaging results, as it indicates that some associations are only detectable with a specific processing. This has implications for several analyses. For example, multiverse analyses that seek to combine results across processings should expect processing‐specific results, and they may miss relevant associations by focusing on consensus results. In addition, our results suggest that ensemble learning from multiple processings may maximize prediction accuracy by leveraging each processing‐specific signal. More generally, some of the differences we observed may be due to processing pipelines (e.g., CAT12 being more prone to registration or segmentation artifacts). Future work aiming to improve processing pipelines may also focus on automatically detecting segmentation or registration issues in order to remove noise that can result from less‐robust processing.

### 4.4 Correlated and Non‐Normal Brain Measurements Impact the False Positive Rate in BWAS

**FIGURE 9:**
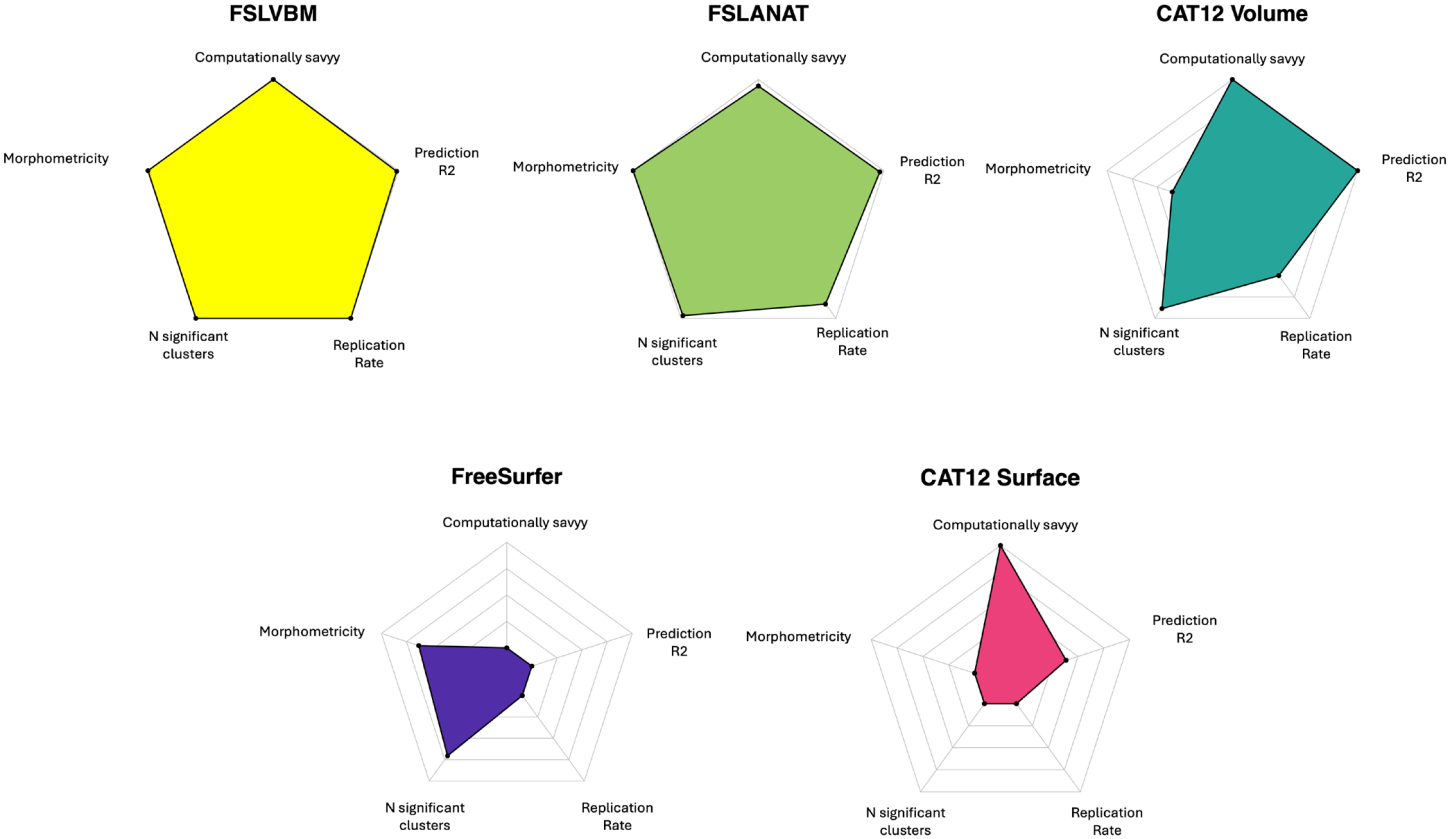
Comparison of all 5 processing methods. This spider plot depicts the performance of all processing methods according to 5 metrics. Each color represents a processing method. The average computational cost ranges from 2 h (FSLVBM, CAT12) to 10 h (FreeSurfer). The prediction accuracy (*R* ^2^) is computed across 12 traits shown in Figure 7 top‐right panel, for which prediction was significantly greater than chance. Average accuracy ranges from 3% (FreeSurfer) to 6% (FSLVBM). Morphometricity estimates were computed across all traits of interest (Figure 3) and range from 1.5% (CAT12 Surface) to 6% (both FSL processings). The number (*N*) of significant clusters across all traits ranges from 190 (CAT12 Surface) to 692 (FSLVBM), and the rate of replicating clusters ranges from 23% (CAT12 Surface and FreeSurfer) to 35% (FSLVBM).

Using simulations, we observed (Figure 5) that processings were well calibrated to ensure FWER < 5% after Bonferroni correction, except for FSLANAT, which demonstrated a small inflation of false positive (FWER = 5.3%) due to non‐normal distributions in gray‐matter measurements. Our results confirmed that Bonferroni is overly stringent for the other processings (FWER in [0.5%; 4.1%]), as it assumes that all tests are independent, even over correlated brain measurements. In particular, BWAS using CAT12 Surface was the most stringent (FWER = 0.5% when applying Bonferroni's correction), which is consistent with the very high level of correlation between brain measurements (Table 2). However, the low FWER was also attributable to non‐normal distributions in voxel‐wise measurements from CAT12 Surface that caused a deflation of test statistics (Figures 5 and 9).

Our findings highlight the necessity of sensitivity analyses in BWAS: either by transforming distributions of brain measurements (e.g., rank inverse normal transformation) and/or visually inspecting associations at a vertex/voxel level to ensure the association is not caused by outliers or tails. We also found that normalizing the distribution of brain measurements (e.g., using RINT) can have a beneficial effect on the false positive rate after Bonferroni correction (it removes inflation of false positives in FSLANAT, and made BWAS analyses on CAT12 Volume less stringent, Figure S3). Overall, our results highlight the need for multiple testing correction more efficient than Bonferroni's, as low FWER (< 5%) leads to reduced statistical power. One may use the optimal significance threshold we derived from simulations, which calibrate the tests to FWER = 5%. Other options include permutations (Nichols and Holmes 2001) (albeit computationally expensive in large samples) and Random Field Theory (Nichols 2012) (although many variations exist, with no unified implementation for volume and surface‐based processing).

### 4.5 The Choice of Processing Influences the Size, Number and Replication Rate of Brain Regions Detected in BWAS

We compared results using optimal significance thresholds for each processing, which ensured a comparable FWER of 5%. We found that the number of significant vertices/voxels varied widely between processing (between 3945 [FSLANAT] and 13,674 [CAT 12 Volume]; Table 3). However, this difference was partly due to differences in cluster sizes, which tend to be larger in the presence of greater correlation across brain measurements (Table 2). For example, 7077 voxels were detected with CAT12 Surface for only 190 clusters, which is consistent with the large amount of correlation between CAT12 Surface measurements (Table 2). The other processing each led to the detection of 544 (FreeSurfer) to 692 (FSLVBM) clusters across all traits (Figure 6, Table 3). Of note, the large number of significant vertex‐wise measurements in FreeSurfer was driven by a handful of extremely large clusters (one of 2547 vertices and two of 900 vertices associated with “High blood pressure”).

**TABLE 3:** Replicating voxels and clusters using the optimal significance threshold across all traits, for all processing.

	Number of significant voxels/vertices	Number of replicating voxels/vertices	Fraction
FSLANAT	3945	1436	0.36
FSLVBM	4277	1489	0.35
CAT12 volume	13,674	5118	0.38
CAT12 surface	7077	1167	0.17
FreeSurfer all modalities	10,936	6818	0.62

	Number of significant clusters	Number of replicating cluster	Fraction
FSLANAT	676	117	0.17
FSLVBM	692	124	0.18
CAT12 volume	635	120	0.19
CAT12 surface	190	41	0.22
FreeSurfer all modalities	544	60	0.11

*Note:* To control for multiple testing in the replication sample, we used Bonferroni corrected significance thresholds of 39,909 for the vertex‐wise level and 2737 for the cluster‐wise inference.

The vertex/voxel‐wise effect sizes also varied between processings (Figure 6), which can contribute to differences in discoverability. Effect sizes were lower for FreeSurfer measurements, which may explain the smaller number of detected clusters compared to FSLVBM. On the other hand, they were maximal for CAT12 Surface, which can somewhat compensate for its lower morphometricity and the deflation of test statistics we have previously observed.

We sought to replicate associations in an independent UK Biobank sample, acquired at different sites. We found a good rate of replication with 17%–22% of the clusters replicating (Table 3), although this was lower for FreeSurfer (only 10%). When removing clusters containing a single vertex/voxel, 33%–35% of clusters replicated using FSLANAT and FSLVBM, 29% using CAT12 Volume and 23% using CAT12 Surface and Freesurfer.

Over all traits considered, using FSLVBM led to the identification of more significant clusters, which displayed the largest replication rate. However, it is important to point out that other processing may maximize the number of identified regions depending on the trait. For example, FreeSurfer led to the identification of more brain regions associated with Parkinson's and Alzheimer's disease, and CAT12 Volume maximized the number of discoveries with Maternal smoking around birth. This variability among processings could be further explored through predictive modeling. We would expect that a greater number of associated regions may result in better prediction accuracy.

### 4.6 Volume Based Processings Yield Better Accuracy in Brain‐Based Prediction

First, we observed that detecting a greater number of significant clusters in BWAS did not necessarily translate in greater prediction accuracy (in an independent UK Biobank sample). For example, maternal smoking was best predicted from gray‐matter regions detected using FSLVBM and FSLANAT (Figure S15), despite FreeSurfer and CAT12 surface yielding > 50 extra clusters (Figure 6). We could not conclude about the prediction of Alzheimer's and Parkinson's disease (where FreeSurfer detected the most clusters) due to the small number of cases. Across the different traits, associations from volume‐based processing gave the best prediction accuracy (Figure S15), which is consistent with their higher replication rate. Of note, clusters of size one contributed little to the prediction from significant regions (Figure S17).

We also found that the top (most significant) voxels/vertices do not always capture the full association from their clusters (Figure 7), either because the selection from *p* value is not optimal, or because several signals can cohabit in a cluster. This is an important observation when trying to build parcimonious and interpretable predictors that rely on a minimal set of brain regions.

Next, we used BLU predictors that improved prediction accuracy by leveraging signals beyond significant brain regions (Figure 7). We showed that several traits could be predicted from gray‐matter structure (e.g., Education age, index of multiple deprivations, number of children, age when first had sex, maternal smoking around birth, alcohol frequency, pains, sleeplessness, high blood pressure, stroke, and diabetes; Figure 7). As previously, volume‐based representations of the gray matter maximized prediction accuracy for a handful of traits (e.g., maternal smoking, alcohol frequency, high blood pressure, and diabetes).

Overall, traits with the largest morphometricity tended to be better predicted. Yet, the proportion of morphometricity that BLUP scores could recover varied between traits, suggesting that some traits are harder to predict than others. For example, BLUP scores could account (on average) for 28% of the morphometricity of maternal smoking but only 20% of that of alcohol use frequency and 10% of the morphometricity of sleeplessness (Figure S16). This suggests that the number of associated brain regions that contribute to the morphometricity varies from one trait to the next, being larger for sleeplessness than for maternal smoking. For instance, some previous work displayed the dependence between the prediction *R* ^2^ and p/N (p being the number of features and N the sample size), for different values of morphometricity and showed that the lower the morphometricity, the larger sample size is needed. In our study, it could be represented with both traits Education Age and Number of children, as they exhibited similar morphometricity estimates (on average 6%) but different percentages of morphometricity predicted (twice larger for “Number of children”), suggesting that Education Age is associated with a more diffuse pattern of gray‐matter regions.

### 4.7 Clusters Comprising a Single Vertex/Voxel Should Be Treated With Caution

We found that across all processing methods, clusters of size one replicated less than larger clusters, with FreeSurfer showing particularly low replication rates (2.1% compared to 7%–9% for other methods). Moreover, building linear predictors using only size one cluster yielded very few significant results (Figure S17) and low prediction accuracy, indicating that while some clusters of this size may represent true signals, the majority may be false positives. Therefore, clusters of size one should be treated with caution, especially in BWAS that rely on FreeSurfer processing. Our results highlight the need for more systematic replication and external validation (e.g., via prediction) to validate neuroimaging findings.

### 4.8 Associated Brain Regions Are Not Always Robust to Processing

We evaluated robustness to processing by comparing whether the clusters identified by the different processing belonged to the same ROIs. We observed that the three volume‐based processing methods detected signals located mostly in the same areas (Figure S14), especially between FSLVBM and FSLANAT, even if the overlap was never perfect. Overall, the overlap of significant ROIs was even lower between FSLVBM and Freesurfer, and CAT12 Surface had poor agreement with all other processing methods (Figure S14). Our results demonstrate that the choice of preprocessing pipeline contributes to variability in the results, which can explain the lack of robustness of some of the published results. However, non‐robust results (i.e., those that are only detected with a specific processing) remain of interest, especially since we showed that each processing captures a unique signal. It underscores the need to carefully interpret both robust and non‐robust results, as different processing methods can capture unique signals. Moreover, understanding where results are least robust could help understand where the unique signal comes from and could be used to develop better processing.

### 4.9 FSLVBM Is a Performant All‐Rounder

Overall, FSLVBM appeared to be the best single processing (green, Figure 9), as it maximized morphometricity estimates, prediction, discoverability, and replication rate. Although FSLANAT (yellow, Figure 9) exhibited mostly similar performance (slightly lower replication rate), we found that it contained voxels with non‐normal distributions, which might create false positive associations. In addition, FSLVBM relies on a well‐established methodology and only requires about ~2 h of processing time per MRI (vs. ~10 for FreeSurfer). Its only downside is that gray‐matter density measured at each voxel is not easily interpretable, in contrast to cortical thickness and surface area that are extracted by CAT12 or FreeSurfer. Thus, we would recommend FSLVBM for future analyses of the UK Biobank, especially when several traits are investigated. For researchers interested in specific diseases/traits, our specific analyses (association, prediction) and other processing may be more suited on a case‐by‐case basis. We have summarized the pros and cons of all considered processing in Table 4.

**TABLE 4:** Summary dataframe of all metrics used to compare processing.

	FSLVBM (volume‐based)	FSLANAT (volume‐based)	CAT12 volume (volume‐based)	CAT12 surface (surface‐based)	FS all modalities (surface‐based)
Computational cost	~2 h from raw nifti Bottleneck: Study specific template	~2 h30 from UK Biobank segmented images Bottleneck: Study specific template	~2 h from raw nifti Use prespecified template Standalone pipeline	~2 h from raw nifti Uses Prespecified template Standalone pipeline	~10 h if done from raw nifti But processing provided by the UK Biobank. ~20 min Enigma‐shape on top of FreeSurfer processing to extract subcortical measurements
Brain measurements	181,544 voxel‐wise measurements Requires defining GM‐mask Same MNI space than Harvard‐Oxford atlas Different MNI space than Julich atlas	184,637 voxel wise measurements Requires defining GM‐mask Same MNI space than Harvard‐Oxford atlas Different MNI space than Julich atlas	192,483 voxel‐ wise measurements Requires defining GM‐mask Different MNI space than Harvard‐Oxford and Julich atlas	299,881vertex wise measurements Only cortical thickness measurement	654,002 vertex‐ wise measurements No cerebellum
Sensitivity to image confounders	64 (49–76) % Contaminated	63 (49–75) % Contaminated	55 (36–71) % Contaminated	21 (18–38) % Contaminated	55 (37–61) % Contaminated
Association with body size	52 (43–59) %	52 (43–59) %	43 (33–46) %	15 (12–16) %	34 (30–41) %
Morphometricity of traits of interest (median [min–max])	5.8 (0.3–35)	5.8 (0.0–36)	3.0 (0.1–32)	1.5 (0.0–13)	4.2 (0.0–33.7)
FWER using Bonferroni	4.1%	5.3% Small fraction of small positive associations due to voxel distributions	2.8%	0.5% Large number of vertices + large sum R2 = overly stringent	2% Large number of vertices, overly stringent
SumR2/Skewness/Kurtosis	SumR2 = 42 Skewness = 0.36 Kurtosis = 3.0	SumR2 = 48 Skewness = 0.35 Kurtosis = 3.1	SumR2 = 240 Skewness = 0.24 Kurtosis = 3.3 Important correlation among voxels	SumR2 = 1471 Skewness = −0.076 Kurtosis = 3.5 Some heavy left tails (negative skewness) Very important correlation among vertices	SumR2 = 105 Skewness = 1.1 Kurtosis = 4.9 Some heavy right tails and leptokurtic (“thin bells”) distributions
Number of significant clusters/voxels Optimal threshold	Voxels = 4277 Clusters = 692	Voxels = 3945 Clusters = 676	Voxels = 13,674 Clusters = 635	Voxels = 7077 Clusters = 190	Voxels = 10,936 Clusters = 544
% replicating rate with (and without) size one clusters	Clusters = 18% (35%)	Clusters = 17% (33%)	Clusters = 19% (29%)	Clusters = 22% (23%)	Clusters = 11% (23%)
Proportion of clusters of size one and their replication rate (*R* = ‐)	60% *R* = 7.2%	63% *R* = 8.5%	45% *R* = 7.4%	9.5% *R* = 7.1%	62% *R* = 2.1%
BLUP prediction *R* ^2^ (%) (median [min–max])	0.21 (0–8.9)	0.40 (0–9.5)	0.32 (0–10.4)	0.12 (0–3.9)	0.20 (0–7.3)

### 4.10 Novel Associations Between Gray‐Matter Structure and Traits of the UK Biobank

Beyond methodological considerations, our results have also highlighted several novel links between gray‐matter structure and UK Biobank traits. For example, we are the first to report the morphometricity of several traits (e.g., number of children, age when first had sex), and to demonstrate that this overall association is partly robust to different gray‐matter representations. Examining these traits is interesting to bridge social science and neuroscience: indeed, they may reflect underlying mechanisms involved in social behavior and help to understand how brain biology influences reproductive choices. We confirmed the implication of the cerebellum in several traits (maternal smoking, Figure 8, diabetes Figure S18), which suggests its function is not limited to movement and motor functions. Specifically, for diabetes, Right_Crus_I and Right_VI showed significant clusters across three volume‐based processes. These results align with some previous work depicting changes in the cerebellar circuit in patients with type II Diabetes (fang et al. 2017).

In addition, we identified hundreds of significant clusters associated with our traits of interest, which shed light on some of the brain regions that contribute to the morphometricity, and some variations in cluster sizes: for instance, for High blood pressure, FreeSurfer exhibited 3 large clusters (Figure S19, dark‐red) located in the Left Putamen (*N* = 2547 vertices), the Right Caudate (*N* = 1006 vertices), and the Left Caudate (*N* = 882 vertices).

Lastly, we built brain‐based predictors that demonstrated significant prediction accuracy (*R* ^2^ in [0.0; 0.1]) for most traits and may be applied to independent samples, where the trait/disease was not collected or is not available.

Interestingly, “Maternal smoking” displayed the largest morphometricity across all processings (*R* ^2^ in [0.04; 0.1]), much larger than the association found with smoking status (*R* ^2^ in [0.00001; 0.0004]). Indeed, previous studies showed that prenatal exposure to maternal smoking can lead to problems in cognitive development (IQ for instance; Shea and Steiner 2008) as well as abnormal brain development (Eiden et al. 2015). Moreover, maternal smoking may reflect not only prenatal toxin exposure but also postnatal environmental factors such as second‐hand smoke, increased stress, or other behavioral correlates of maternal smoking, further strengthening the associations we detected (Cornelius and Day 2000). Some previous research showed some association between smoking exposure during pregnancy and smaller gray matter volume in the inferotemporal and parahippocampal regions, and with smaller surface area in the parahippocampal and postcentral regions (Ekblad et al. 2023). These results were partially consistent with our findings, as all processing showed replicating clusters in the right hippocampus and two processings in the amygdala (Figure 8). We also identified some regions in the metathalamus and calcarine sulcus which are less common but could be involved in addiction (Huang et al. 2018).

## 5 Limitations

Our results suggest that it is important to include imaging and body size covariates when performing brain imaging analysis in the UKBiobank. However, the list of recommended covariates may be refined in the future, as more confounders are evaluated. In addition, some processings seem to capture more information on average (e.g., FSL and FreeSurfer). However, the best processing may depend on the trait of interest, and our results cannot be extrapolated to all traits and disorders.

We performed our analyses on the UK Biobank, and we do not know whether our results would generalize to other studies that have acquired images of different quality, or used different scanners and acquisition protocols, that may influence the amount of correlation or the distribution of measurements. In particular, one should know that UKBiobank is likely the most homogeneous research dataset in terms of image acquisition and it would be important to see whether our results would generalize to other datasets where MRI scanner are more varied or acquisition parameters are not harmonized. Furthermore, the heterogeneity of clinical routine datasets is usually even larger and, again, it remains to be studied which processing would be the most reliable in such context.

Our primary focus was on ensuring the robustness of the findings—the ability to detect consistent results across different processing—in published results, while also addressing reproducibility and replication. To encourage future work, we will make our code and summary statistics openly available, and the processed data will be returned to the UK Biobank for others to reproduce and extend our analyses. Additionally, we evaluated replication and prediction in an independent dataset within the same cohort. That said, testing these findings in other cohorts with different scanners, phenotypes, and datasets would be an interesting future research opportunity, offering the chance to further validate and expand upon our work.

We only include MRI processing pipelines that used default options or off‐the‐shelf implementations, even though many variations exist. For instance, FreeSurfer may be used with T1w only. CAT12 pipelines use a template created from the IXI dataset, which may contribute to the differences with FSL‐based processings that used a study‐specific template. Other variations in processing may come from using newer software versions (e.g., CAT12 12.9, FreeSurfer 7.0) or selecting different resolutions of the output map (voxel size 1 or 1.5 mm for volume‐based processing, different fsaverage surfaces in surface‐based processing). More generally, some of the differences we observed may be due to processing pipelines (e.g., CAT12 being more prone to registration or segmentation artifacts). Future work aiming to improve processing pipelines may also focus on automatically detecting segmentation or registration issues in order to remove noise that can result from less‐robust processing.

Thus, we cannot claim that one software or brain representation is superior to another at this stage, as we have only explored some of the main branches of the multiverse. However, the results we reported here could be used to benchmark and guide the development of processing pipelines. For example, processing that increases the morphometricity against our reference that used the same software is likely to lead to improved discoverability and brain‐based prediction. More generally, processing that captures more morphometricity and less processing‐specific signal would contribute to more robust results in neuroimaging analyses.

The interpretation of the gray‐matter density in voxel‐based representation remains a challenge. Unlike cortical thickness, which has a clear interpretation (Fischl and Dale 2000), gray‐matter density is a more abstract measure, and its biological significance is less well‐understood (Ashburner and Friston 2000). This makes the results harder to interpret from a biological perspective, which may justify using surface‐based representations.

## Supporting information


**Data S1:** hbm70372‐sup‐0001‐Supinfo.docx.

## Data Availability

The data that support the findings of this study are available from the corresponding author upon reasonable request. The code is available at: https://github.com/elisedlzt/Choice‐of‐processing‐pipelines‐for‐T1w‐brain‐MRI‐impacts‐association‐and‐prediction‐analyses. All summary statistics are available at: https://identifiers.org/neurovault.collection:21541.
