# Supplementary material for: Choice of Processing Pipelines for T1‐Weighted Brain MRI Impacts Association and Prediction Analyses
Source: Hum Brain Mapp. 2025 Oct 30;46(16):e70372. doi: 10.1002/hbm.70372 (PMC12572822; doi:10.1002/hbm.70372)
Supplement: Supplementary file 1 — Data S1: hbm70372‐sup‐0001‐Supinfo.docx. [file HBM-46-e70372-s001.docx]

[Supplementary Figure 1 : **Effect of template sample size on image registration accuracy for FSLVBM -left- and FSLANAT -right.** In both FSL processing , images are non-linearly registered to a study-specific template. To obtain the optimal template, we generated templates using samples of varying sizes (x-axis) and registered a test set of 300 participants on these templates. Then, we calculated, for each participant, the correlation (y-axis) between the registered image and that registered to the template made with the largest sample (3000). 2](#_Toc179374642)

[Supplementary Figure 2 : **Grey-matter masks from the different Voxel-based processing**. With N, number of voxels kept for each processing, after exclusion of voxels with mean lower than 0.1 and variance lower than 0.01. 3](#_Toc179374643)

[Supplementary Figure 3 : **Histograms of diagonal and off-diagonal elements of the UK Biobank Brain Relatedness Matrix (BRM) before (left panels) and after (right panels) Quality Control (QC).** Each row/color corresponds to a processing. Of note, diagonal elements consist of the mean square of the participant’s brain measurements, and off-diagonal elements to the covariances between two individuals. Participants with extreme values(diagonal element > 2.5 or off-diagonal element > 6 standard deviations) were excluded of the analysis, as they might have ‘outstanding’ brains. 4](#_Toc179374644)

[Supplementary Figure 4 :  **Brain regions contribution to morphometricity estimates of potential confounders, for each processing.** As CAT12 Surface captures signal from the cortical thickness only, we did not include it in this plot. For all three volume based processing we subdivided the brain into 3 regions : cortical, subcortical and cerebellar. As for Free Surfer, we subdivided it into cortical thickness, cortical area and subcortical region.For instance FSLVBM explained a variance of 0.75 for the covariates `Discrepancy between T1 and template`. The cortical area accounted for 68% of this signal vs. 21% for the subcortical area and 11% for the cerebellum. 34](#_Toc179374645)

[Supplementary Figure 5 : **Brain regions contribution to morphometricity estimates of traits of interest, for each processing, controlling for covariates.** As CAT12 Surface captures signal from the cortical thickness only, we did not include it in this plot. For all three volume-based processing we subdivided the brain into 3 regions: cortical, subcortical and cerebellar. As for Free Surfer, we subdivided it into cortical thickness, cortical area and subcortical region. Regions exhibited not significant contribution remained unlabelled. 35](#_Toc179374646)

[Supplementary Figure 6 : **Concordance of morphometricity estimates between brain measurements and after rank inverse normalization of brain measurements.** Morphometricity from default brain measurements is shown as the x-axis and morphometricity from rank-normalized voxels/vertices is shown as the y-axis. The vertical and horizontal bars show the 95% confidence intervals in the two samples. 36](#_Toc179374647)

[Supplementary Figure 7 : **Concordance of Morphometricity estimates between the replication and discovery sample.** Morphometricity in the discovery sample is shown as the x-axis and morphometricity in the replication dataset is shown as the y-axis. The vertical and horizontal bars show the 95% confidence intervals in the two samples. The left panel shows the morhometricity estimates of possible confounders (controlling for standard covariates). The right panel depicts morphometricities of the traits of interest when controlling for all covariates (standard and confounders). 36](#_Toc179374648)

[Supplementary Figure 8 : **Heatmap of morphometricity** i**ncrease when fitting two grey-matter representation in the model, across all traits of interest.** Each heatmap illustrates the percentage of variance explained (morphometricity) when combining two processing methods. The rate of increase is shown in parentheses, and diagonal values represents the morphometricity of each processing method alone. Rows indicate the reference processing and columns the added processing. For example, on the Alcohol frequency plot, the element at coordinates (1,2) corresponds to the result of of FreeSurfer All Modalities added to FSLVBM, resulting in an estimate 1.21 higher than FSLVBM alone (14.4%). Symmetrically, adding FSLVBM to FreeSurfer All Modalities yields a different rate of increase (1.75) since both processing alone do not explain the same percentage of variance. 37](#_Toc179374649)

[Supplementary Figure 9 : **Role of cerebellar measurements in FSLVBM morphometricity estimates.** We found that all processing do not capture the same signal with some part being unique. As FreeSurfer do not measure the cerebellum, we wondered whether this discrepancy originated from that region. We compared morphometricity estimates when adding FSL (all measurements) and Free Surfer vs. FSL (without cerebellar measurements) and FreeSurfer. The vertical and horizontal bars show the 95% confidence intervals 38](#_Toc179374650)

[Supplementary Figure 10 : **Q-Q plot of the minimal p-values for each voxel/vertex across the 1000 random traits without any adjustments (left panel) and with prior normalization (right panel).** Minimal p-value are presented in the log10 scale. Grey lines show the 95% confidence interval. Therefore, processing crossing this threshold mean that they exhibit p-values larger/smaller than expected by chance. 40](#_Toc179374651)

[Supplementary Figure 11 : **Median Z-score without multiple testing control, after rank-inverse normalisation of brain measurements.** Left panel depicts the median absolute z-score for all traits of interest. The right boxplot displays the overall distribution of these scores. 40](#_Toc179374652)

[Supplementary Figure 12 : **Concordance of number of significant clusters after Bonferroni correction or optimal significance thresholds.** 41](#_Toc179374653)

[Supplementary Figure 13 : **Number of regions of interest exhibiting at least one significant cluster, across all traits.** 41](#_Toc179374654)

[Supplementary Figure 14 : **Heatmap of number of common significant regions of interest, for top 4 traits exhibiting higher number of significant clusters.** Each heatmap depicts the number of regions of interest exhibiting at least one significant cluster for both processing method. Diagonal values represents the total number of significant cluster for each processing. For example, in the Maternal smoking plot, the element (3,1) is 32 indicating that 32 same regions of interest were significant for both CAT12 Volume and FSLVBM processing, whereas FSLVBM captures 55 significant clusters and CAT12 Volume 67. 42](#_Toc179374655)

[Supplementary Figure 15 : **Linear prediction from top significant voxels/vertices, across all traits and processing methods, after optimal thresholds correction, and comparison with Bonferroni correction.** The left panel depicts the R2 prediction from top significant voxels/vertices with optimal thresholds correction : the y-axis represents the difference between the R^2^prediction from the model with top voxels/ vertices minus R^2^ of baseline model. Red stars indicate significant log-likelihood ratio test after Bonferroni correction (p < 0.05/6*29). The right panel depicts the R2 comparison when using Bonferroni correction vs. optimal thresholds 43](#_Toc179374656)

[Supplementary Figure 16 : **Fraction of predicted morphometricity across traits exhibiting significant prediction, for all processing methods.** Left panel represents the fraction of predicted morphometricity (e.g the R2 prediction divided by the morphometricity for this trait and processing) across all 6 traits included in the right panel. Right panel represents the fraction of predicted morphometricity for the 6 traits exhibiting significant prediction (Figure 7), across all six processing methods. 43](#_Toc179374657)

[Supplementary Figure 17 : **Linear prediction from size one clusters, across all traits and processing methods.** 44](#_Toc179374658)

[Supplementary Figure 18 : **Replicating cluster locations by Region of Interest for Diabetes.** 45](#_Toc179374659)

[Supplementary 19: **Replicating cluster locations by Region of Interest for High blood pressure.** 46](#_Toc179374660)

[Supplementary Figure 20 : **Replicating cluster locations by Region of Interest for Alcohol frequency.** 47](#_Toc179374661)

[Supplementary Figure 21: **Replicating cluster locations by Region of Interest for Number of children.** 48](#_Toc179374662)

[Supplementary Figure 22: **Replicating cluster locations by Region of Interest for Stroke.** 49](#_Toc179374663)

*Supplementary Appendix 1:* ***Linear Mixed Model to estimate morphometricity 50***

Supplementar*ary Appendix 2 :* ***Extension of LMM 51***


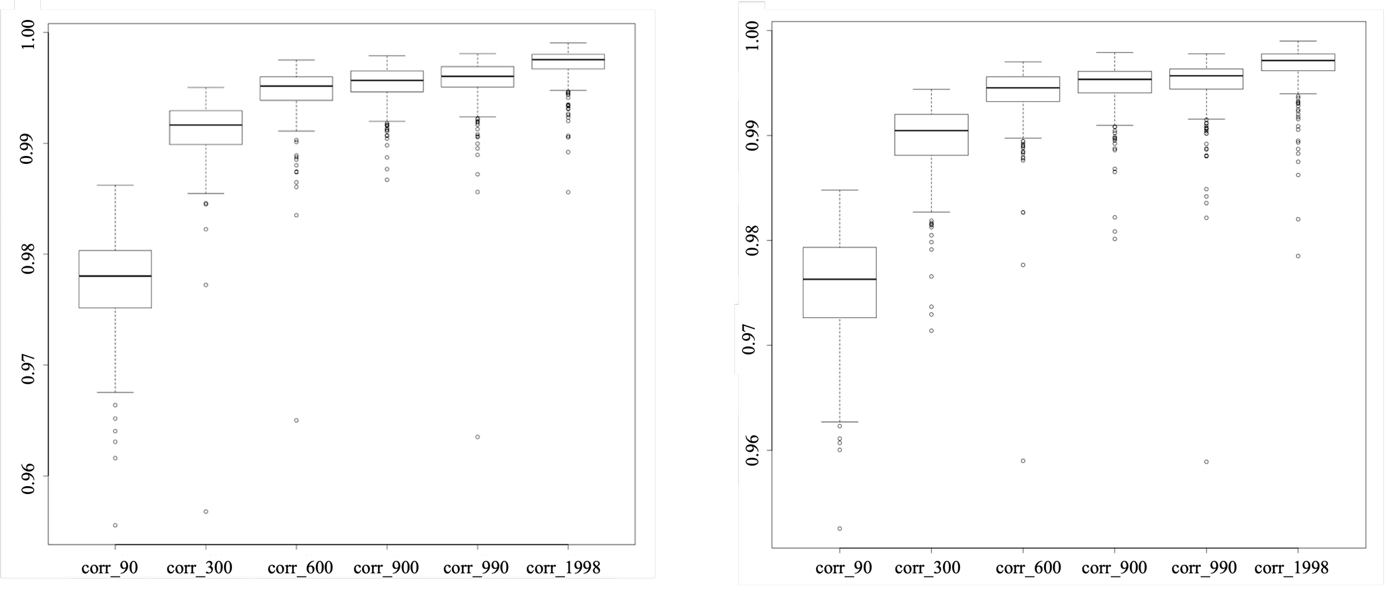


Supplementary Figure 1 : **Effect of template sample size on image registration accuracy for FSLVBM -left- and FSLANAT -right.** In both FSL processing , images are non-linearly registered to a study-specific template. To generate the templates, we randomly selected participant subsets of varying sizes (x-axis), ensuring balanced sex distribution and equal representation from all three main imaging centers (we excluded Bristol as only comprising 52 brain images). For each subset, we applied steps 1–3 of the FSLVBM pipelines to construct a corresponding template. Separately, we defined a test dataset of 300 individuals—balanced by sex and main imaging center—who were not included in any of the template subsets. Each image of the test set was then processed through the full FSLVBM pipeline using each of the generated templates, resulting in six complete sets of registered images (one per template size). Finally, for each set, we computed the correlation (y-axis), using the cor function in R, between all registered images processed and those processed using the largest sample (3,000 participants). Same method was applied for FSLANAT. Overall, for both FSLVBM and FSLANAT, we observed that the correlation increased with template size but plateaued around n=600 (r>0.995). Based on this finding, we selected the n=600 template as a representative balance between accuracy and computational efficiency, and used it to process all images for both the FSLVBM and FSLANAT pipelines.


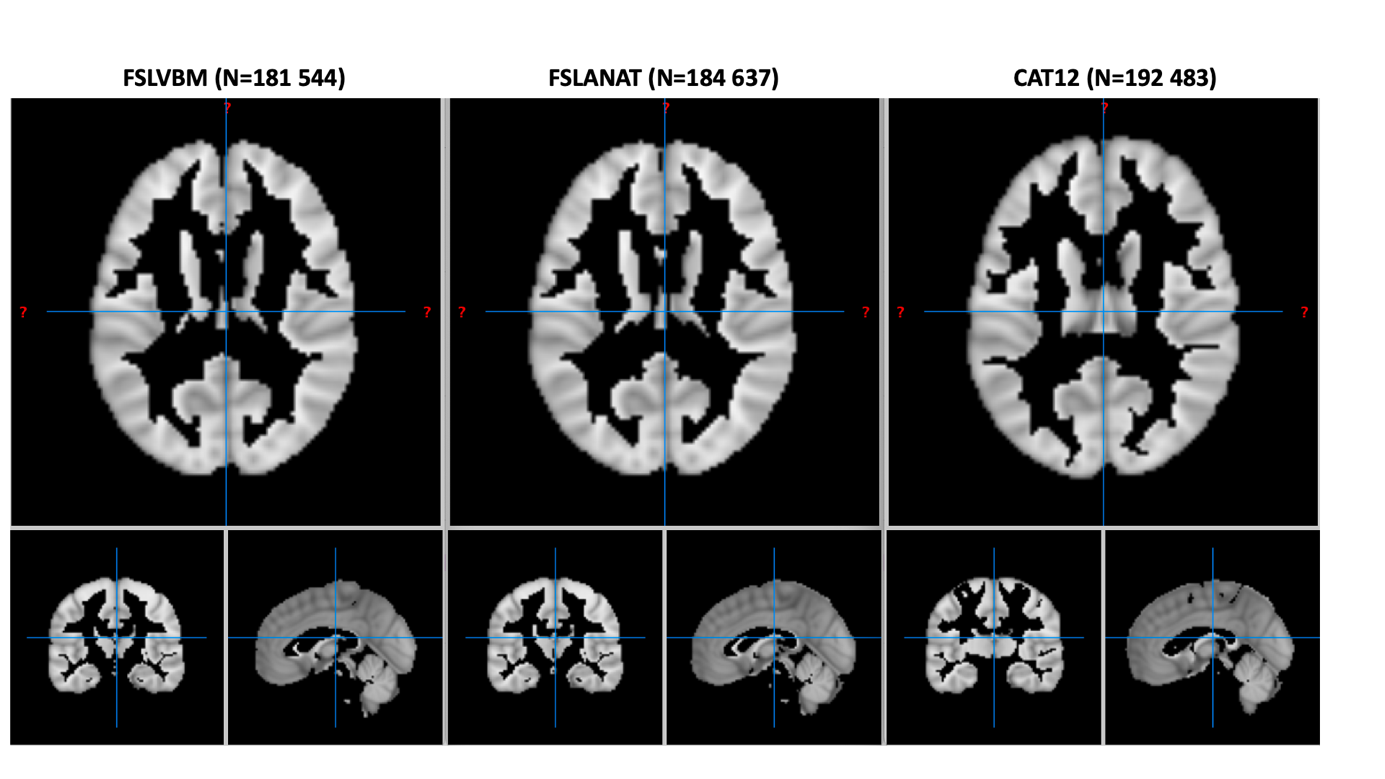


Supplementary Figure 2 : **Grey-matter masks from the different Voxel-based processing**. With N, number of voxels kept for each processing, after exclusion of voxels with mean lower than 0.1 and variance lower than 0.01.

Supplementary Figure 3 : **Histograms of diagonal and off-diagonal elements of the UK Biobank Brain Relatedness Matrix (BRM) before (left panels) and after (right panels) Quality Control (QC).** Each row/color corresponds to a processing. Of note, diagonal elements consist of the mean square of the participant’s brain measurements, and off-diagonal elements to the covariances between two individuals. Participants with extreme values(diagonal element > 2.5 or off-diagonal element > 6 standard deviations) were excluded of the analysis, as they might have ‘outstanding’ brains.

Table 1 : **Mean SumR2 across all Regions Of Interests and all processings.** SumR^2^ quantifies the overall amount of correlation in the brain. The largest the more correlated the voxels/vertices are within the region. To label brain regions, we used Julich Atlas for the cortical area, Harvard Oxford for subcortical and Diedrichsen atlas for cerebellar area.

| **Processing** | **ROI** | **ROI Location** | **Mean SumR2** |
| --- | --- | --- | --- |
| FSLVBM | Left_Crus_II | Cerebellum | 401 |
| FSLVBM | Right_Crus_II | Cerebellum | 394 |
| FSLVBM | Left_VIIb | Cerebellum | 389 |
| FSLVBM | Right_VIIb | Cerebellum | 386 |
| FSLVBM | Right_VIIIa | Cerebellum | 301 |
| FSLVBM | Left_Crus_I | Cerebellum | 291 |
| FSLVBM | Right_Crus_I | Cerebellum | 285 |
| FSLVBM | Left_VIIIa | Cerebellum | 277 |
| FSLVBM | Right_VI | Cerebellum | 267 |
| FSLVBM | Left_Caudate | Subcortical | 258 |
| FSLVBM | Right_Caudate | Subcortical | 253 |
| FSLVBM | Left_VI | Cerebellum | 238 |
| FSLVBM | Fundus_of_Caudate_Nucleus,_ventral_Striatum_(Basal_Ganglia) | Cortical | 214 |
| FSLVBM | Medial_Accumbens,_ventral_Striatum_(Basal_Ganglia) | Cortical | 206 |
| FSLVBM | Left_Accumbens | Subcortical | 203 |
| FSLVBM | MF_(Amygdala) | Cortical | 194 |
| FSLVBM | Right_Accumbens | Subcortical | 192 |
| FSLVBM | Vermis_VIIb | Cerebellum | 186 |
| FSLVBM | Vermis_IX | Cerebellum | 168 |
| FSLVBM | Vermis_VIIIb | Cerebellum | 154 |
| FSLVBM | Left_VIIIb | Cerebellum | 149 |
| FSLVBM | VTM_(Amygdala) | Cortical | 147 |
| FSLVBM | Right_Putamen | Subcortical | 146 |
| FSLVBM | Right_VIIIb | Cerebellum | 143 |
| FSLVBM | Vermis_VIIIa | Cerebellum | 142 |
| FSLVBM | CA3_(Hippocampus) | Cortical | 140 |
| FSLVBM | Left_Putamen | Subcortical | 139 |
| FSLVBM | DG_(Hippocampus) | Cortical | 136 |
| FSLVBM | Right_IX | Cerebellum | 130 |
| FSLVBM | CA2_(Hippocampus) | Cortical | 127 |
| FSLVBM | Fundus_of_Putamen,_Ventral_Striatum_(Basal_Ganglia) | Cortical | 126 |
| FSLVBM | Left_IX | Cerebellum | 125 |
| FSLVBM | Area_Ph3_(PhG) | Cortical | 119 |
| FSLVBM | SF_(Amygdala) | Cortical | 119 |
| FSLVBM | Right_V | Cerebellum | 115 |
| FSLVBM | Lateral_Accumbens,_ventral_Striatum_(Basal_Ganglia) | Cortical | 111 |
| FSLVBM | Vermis_Crus_II | Cerebellum | 111 |
| FSLVBM | LB_(Amygdala) | Cortical | 108 |
| FSLVBM | Area_s32_(sACC) | Cortical | 108 |
| FSLVBM | Left_V | Cerebellum | 107 |
| FSLVBM | Terminal_islands_(Basal_Forebrain) | Cortical | 107 |
| FSLVBM | CM_(Amygdala) | Cortical | 105 |
| FSLVBM | BST_(Bed_Nucleus) | Cortical | 104 |
| FSLVBM | Area_Ph2_(PhG) | Cortical | 102 |
| FSLVBM | HC-Transsubiculum_(Hippocampus) | Cortical | 102 |
| FSLVBM | Area_FG3_(FusG) | Cortical | 101 |
| FSLVBM | Area_s24_(sACC) | Cortical | 100 |
| FSLVBM | Area_p24ab_(pACC) | Cortical | 99 |
| FSLVBM | Area_Ig1_(Insula) | Cortical | 98 |
| FSLVBM | Area_CoS1_(CoS) | Cortical | 98 |
| FSLVBM | Entorhinal_Cortex | Cortical | 98 |
| FSLVBM | CA1_(Hippocampus) | Cortical | 96 |
| FSLVBM | Area_FG4_(FusG) | Cortical | 93 |
| FSLVBM | Subiculum_(Hippocampus) | Cortical | 93 |
| FSLVBM | Right_Amygdala | Subcortical | 93 |
| FSLVBM | Area_OP2_(POperc) | Cortical | 92 |
| FSLVBM | Area_Ph1_(PhG) | Cortical | 92 |
| FSLVBM | Ch_123_(Basal_Forebrain) | Cortical | 90 |
| FSLVBM | Area_FG1_(FusG) | Cortical | 90 |
| FSLVBM | Area_Id4_(Insula) | Cortical | 90 |
| FSLVBM | Area_Ig2_(Insula) | Cortical | 88 |
| FSLVBM | Area_33_(ACC) | Cortical | 88 |
| FSLVBM | Area_Id5_(Insula) | Cortical | 86 |
| FSLVBM | Vermis_VI | Cerebellum | 86 |
| FSLVBM | Area_Id6_(Insula) | Cortical | 85 |
| FSLVBM | Area_Fo2_(OFC) | Cortical | 82 |
| FSLVBM | Area_Ig3_(Insula) | Cortical | 81 |
| FSLVBM | VP,_ventral_Pallidum_(Basal_Ganglia) | Cortical | 79 |
| FSLVBM | HATA_(Hippocampus) | Cortical | 78 |
| FSLVBM | Tuberculum_(Basal_Forebrain) | Cortical | 78 |
| FSLVBM | Frontal-to-Temporal-II_(GapMap) | Cortical | 77 |
| FSLVBM | Area_Id1_(Insula) | Cortical | 76 |
| FSLVBM | Right_I-IV | Cerebellum | 75 |
| FSLVBM | Right_Hippocampus | Subcortical | 75 |
| FSLVBM | Area_Op7_(Frontal_Operculum) | Cortical | 75 |
| FSLVBM | Area_Id3_(Insula) | Cortical | 75 |
| FSLVBM | Area_FG2_(FusG) | Cortical | 75 |
| FSLVBM | Left_Thalamus | Subcortical | 74 |
| FSLVBM | Right_Thalamus | Subcortical | 74 |
| FSLVBM | Frontal-to-Occipital_(GapMap) | Cortical | 73 |
| FSLVBM | Left_Lateral_Ventricle | Subcortical | 72 |
| FSLVBM | IF_(Amygdala) | Cortical | 72 |
| FSLVBM | Right_Lateral_Ventricle | Subcortical | 72 |
| FSLVBM | Area_Id9_(Insula) | Cortical | 71 |
| FSLVBM | Area_Id2_(Insula) | Cortical | 70 |
| FSLVBM | CGL_(Metathalamus) | Cortical | 70 |
| FSLVBM | Left_I-IV | Cerebellum | 70 |
| FSLVBM | Area_OP3_(POperc) | Cortical | 70 |
| FSLVBM | Area_Id7_(Insula) | Cortical | 68 |
| FSLVBM | Area_p24c_(pACC) | Cortical | 67 |
| FSLVBM | Area_TE_1.1_(HESCHL) | Cortical | 67 |
| FSLVBM | Area_Id8_(Insula) | Cortical | 67 |
| FSLVBM | Area_Ia1_(Insula) | Cortical | 65 |
| FSLVBM | Vermis_X | Cerebellum | 65 |
| FSLVBM | Left_Amygdala | Subcortical | 64 |
| FSLVBM | Area_Ia2_(Insula) | Cortical | 63 |
| FSLVBM | Area_hOc1_(V1,_17,_CalcS) | Cortical | 63 |
| FSLVBM | Area_4p_(PreCG) | Cortical | 63 |
| FSLVBM | Area_hOc6_(POS) | Cortical | 62 |
| FSLVBM | Area_hOc4v_(LingG) | Cortical | 62 |
| FSLVBM | Area_Op5_(Frontal_Operculum) | Cortical | 62 |
| FSLVBM | Area_Fo1_(OFC) | Cortical | 60 |
| FSLVBM | Area_25_(sACC) | Cortical | 59 |
| FSLVBM | Area_7M_(SPL) | Cortical | 57 |
| FSLVBM | Area_Op6_(Frontal_Operculum) | Cortical | 56 |
| FSLVBM | Area_5Ci_(SPL) | Cortical | 54 |
| FSLVBM | Area_STS1_(STS) | Cortical | 53 |
| FSLVBM | Temporal-to-Parietal_(GapMap) | Cortical | 53 |
| FSLVBM | Area_Id10_(Insula) | Cortical | 53 |
| FSLVBM | Area_Ia3_(Insula) | Cortical | 53 |
| FSLVBM | Area_5M_(SPL) | Cortical | 53 |
| FSLVBM | Area_Op8_(Frontal_Operculum) | Cortical | 51 |
| FSLVBM | Area_OP1_(POperc) | Cortical | 51 |
| FSLVBM | Area_TI_(STG) | Cortical | 51 |
| FSLVBM | Area_hOc2_(V2,_18) | Cortical | 51 |
| FSLVBM | Left_X | Cerebellum | 50 |
| FSLVBM | Right_X | Cerebellum | 50 |
| FSLVBM | Area_TE_1.2_(HESCHL) | Cortical | 50 |
| FSLVBM | Area_6ma_(preSMA,_mesial_SFG) | Cortical | 49 |
| FSLVBM | Area_4a_(PreCG) | Cortical | 48 |
| FSLVBM | Area_TeI_(STG) | Cortical | 48 |
| FSLVBM | CGM_(Metathalamus) | Cortical | 48 |
| FSLVBM | Area_p32_(pACC) | Cortical | 48 |
| FSLVBM | Area_PFcm_(IPL) | Cortical | 47 |
| FSLVBM | Left_Hippocampus | Subcortical | 47 |
| FSLVBM | Area_OP4_(POperc) | Cortical | 47 |
| FSLVBM | Area_3a_(PostCG) | Cortical | 47 |
| FSLVBM | Area_TE_3_(STG) | Cortical | 45 |
| FSLVBM | Ch_4_(Basal_Forebrain) | Cortical | 44 |
| FSLVBM | Area_TE_1.0_(HESCHL) | Cortical | 44 |
| FSLVBM | STN_(Subthalamus) | Cortical | 44 |
| FSLVBM | Area_STS2_(STS) | Cortical | 44 |
| FSLVBM | Frontal-to-Temporal-I_(GapMap) | Cortical | 44 |
| FSLVBM | Area_hIP5_(IPS) | Cortical | 42 |
| FSLVBM | Area_hIP1_(IPS) | Cortical | 42 |
| FSLVBM | Area_6mp_(SMA,_mesial_SFG) | Cortical | 42 |
| FSLVBM | Area_hIP7_(IPS) | Cortical | 42 |
| FSLVBM | Area_IFS4_(IFS) | Cortical | 40 |
| FSLVBM | Area_6d3_(SFS) | Cortical | 40 |
| FSLVBM | Area_Fo3_(OFC) | Cortical | 40 |
| FSLVBM | Area_TE_2.1_(STG) | Cortical | 40 |
| FSLVBM | Area_hOc3v_(LingG) | Cortical | 40 |
| FSLVBM | Area_Fo5_(OFC) | Cortical | 39 |
| FSLVBM | Area_Fp2_(FPole) | Cortical | 39 |
| FSLVBM | Area_TE_2.2_(STG) | Cortical | 39 |
| FSLVBM | Area_hIP8_(IPS) | Cortical | 39 |
| FSLVBM | Area_3b_(PostCG) | Cortical | 38 |
| FSLVBM | Area_Fo7_(OFC) | Cortical | 38 |
| FSLVBM | Area_6d1_(PreCG) | Cortical | 38 |
| FSLVBM | Area_Fo4_(OFC) | Cortical | 38 |
| FSLVBM | Area_Fo6_(OFC) | Cortical | 38 |
| FSLVBM | Area_hIP4_(IPS) | Cortical | 38 |
| FSLVBM | Area_IFS2_(IFS) | Cortical | 38 |
| FSLVBM | Area_hOc5_(LOC) | Cortical | 37 |
| FSLVBM | Frontal-II_(GapMap) | Cortical | 37 |
| FSLVBM | Area_Fp1_(FPole) | Cortical | 36 |
| FSLVBM | Area_6d2_(PreCG) | Cortical | 35 |
| FSLVBM | Area_IFJ2_(IFS,PreCS) | Cortical | 35 |
| FSLVBM | Area_44_(IFG) | Cortical | 35 |
| FSLVBM | Area_PGp_(IPL) | Cortical | 35 |
| FSLVBM | Area_8v2_(MFG) | Cortical | 35 |
| FSLVBM | Area_IFS1_(IFS) | Cortical | 34 |
| FSLVBM | Area_hOc3d_(Cuneus) | Cortical | 34 |
| FSLVBM | Brain_Stem | Subcortical | 34 |
| FSLVBM | Area_hOc4la_(LOC) | Cortical | 34 |
| FSLVBM | Area_PFt_(IPL) | Cortical | 34 |
| FSLVBM | Frontal-I_(GapMap) | Cortical | 33 |
| FSLVBM | Area_IFJ1_(IFS,PreCS) | Cortical | 33 |
| FSLVBM | Area_SFS2_(SFS) | Cortical | 33 |
| FSLVBM | Area_2_(PostCS) | Cortical | 33 |
| FSLVBM | Area_1_(PostCG) | Cortical | 33 |
| FSLVBM | Area_Op9_(Frontal_Operculum) | Cortical | 32 |
| FSLVBM | Area_8d1_(SFG) | Cortical | 32 |
| FSLVBM | Area_8v1_(MFG) | Cortical | 32 |
| FSLVBM | Area_IFS3_(IFS) | Cortical | 32 |
| FSLVBM | Area_PFop_(IPL) | Cortical | 32 |
| FSLVBM | Area_MFG2_(MFG) | Cortical | 31 |
| FSLVBM | Area_hIP3_(IPS) | Cortical | 31 |
| FSLVBM | Area_SFS1_(SFS) | Cortical | 31 |
| FSLVBM | Area_45_(IFG) | Cortical | 31 |
| FSLVBM | Area_PF_(IPL) | Cortical | 29 |
| FSLVBM | Area_hOc4lp_(LOC) | Cortical | 29 |
| FSLVBM | Area_TPJ_(STG/SMG) | Cortical | 29 |
| FSLVBM | Area_PFm_(IPL) | Cortical | 28 |
| FSLVBM | Area_MFG1_(MFG) | Cortical | 28 |
| FSLVBM | Right_Pallidum | Subcortical | 28 |
| FSLVBM | Area_hOc4d_(Cuneus) | Cortical | 27 |
| FSLVBM | Area_PGa_(IPL) | Cortical | 27 |
| FSLVBM | Area_hPO1_(POS) | Cortical | 27 |
| FSLVBM | Area_hIP6_(IPS) | Cortical | 27 |
| FSLVBM | Area_hIP2_(IPS) | Cortical | 26 |
| FSLVBM | Area_8d2_(SFG) | Cortical | 26 |
| FSLVBM | Ventral_Dentate_Nucleus_(Cerebellum) | Cortical | 25 |
| FSLVBM | Area_5L_(SPL) | Cortical | 24 |
| FSLVBM | Left_Pallidum | Subcortical | 24 |
| FSLVBM | Area_7PC_(SPL) | Cortical | 23 |
| FSLVBM | Area_7P_(SPL) | Cortical | 23 |
| FSLVBM | Interposed_Nucleus_(Cerebellum) | Cortical | 20 |
| FSLVBM | Area_7A_(SPL) | Cortical | 19 |
| FSLVBM | Dorsal_Dentate_Nucleus_(Cerebellum) | Cortical | 18 |
| FSLVBM | Vermis_Crus_I | Cerebellum | 18 |
| **Processing** | **ROI** | **ROI Location** | **Mean SumR2** |
| FSLANAT | Right_VIIb | Cerebellum | 503 |
| FSLANAT | Left_VIIb | Cerebellum | 488 |
| FSLANAT | Left_Crus_II | Cerebellum | 477 |
| FSLANAT | Right_Crus_II | Cerebellum | 471 |
| FSLANAT | Right_VIIIa | Cerebellum | 377 |
| FSLANAT | Left_VIIIa | Cerebellum | 372 |
| FSLANAT | Right_Crus_I | Cerebellum | 305 |
| FSLANAT | Left_Crus_I | Cerebellum | 287 |
| FSLANAT | Right_VI | Cerebellum | 283 |
| FSLANAT | Left_VI | Cerebellum | 256 |
| FSLANAT | Right_Caudate | Subcortical | 250 |
| FSLANAT | Left_Caudate | Subcortical | 249 |
| FSLANAT | Right_Accumbens | Subcortical | 232 |
| FSLANAT | Vermis_VIIIb | Cerebellum | 220 |
| FSLANAT | Left_Accumbens | Subcortical | 219 |
| FSLANAT | Vermis_IX | Cerebellum | 210 |
| FSLANAT | Fundus_of_Caudate_Nucleus,_ventral_Striatum_(Basal_Ganglia) | Cortical | 207 |
| FSLANAT | Vermis_VIIIa | Cerebellum | 199 |
| FSLANAT | Left_VIIIb | Cerebellum | 187 |
| FSLANAT | Medial_Accumbens,_ventral_Striatum_(Basal_Ganglia) | Cortical | 185 |
| FSLANAT | Vermis_Crus_II | Cerebellum | 170 |
| FSLANAT | Left_IX | Cerebellum | 163 |
| FSLANAT | Vermis_VIIb | Cerebellum | 162 |
| FSLANAT | Right_IX | Cerebellum | 161 |
| FSLANAT | MF_(Amygdala) | Cortical | 159 |
| FSLANAT | Area_Ph3_(PhG) | Cortical | 158 |
| FSLANAT | Right_VIIIb | Cerebellum | 155 |
| FSLANAT | Right_Putamen | Subcortical | 154 |
| FSLANAT | Left_Putamen | Subcortical | 146 |
| FSLANAT | CA3_(Hippocampus) | Cortical | 139 |
| FSLANAT | DG_(Hippocampus) | Cortical | 133 |
| FSLANAT | SF_(Amygdala) | Cortical | 132 |
| FSLANAT | CA2_(Hippocampus) | Cortical | 129 |
| FSLANAT | Right_V | Cerebellum | 127 |
| FSLANAT | VTM_(Amygdala) | Cortical | 126 |
| FSLANAT | BST_(Bed_Nucleus) | Cortical | 122 |
| FSLANAT | Left_V | Cerebellum | 122 |
| FSLANAT | Area_Ph2_(PhG) | Cortical | 121 |
| FSLANAT | Fundus_of_Putamen,_Ventral_Striatum_(Basal_Ganglia) | Cortical | 121 |
| FSLANAT | Vermis_VI | Cerebellum | 121 |
| FSLANAT | Area_FG3_(FusG) | Cortical | 119 |
| FSLANAT | Entorhinal_Cortex | Cortical | 117 |
| FSLANAT | Lateral_Accumbens,_ventral_Striatum_(Basal_Ganglia) | Cortical | 117 |
| FSLANAT | Area_Ph1_(PhG) | Cortical | 113 |
| FSLANAT | Terminal_islands_(Basal_Forebrain) | Cortical | 112 |
| FSLANAT | Area_FG1_(FusG) | Cortical | 108 |
| FSLANAT | CM_(Amygdala) | Cortical | 108 |
| FSLANAT | HC-Transsubiculum_(Hippocampus) | Cortical | 108 |
| FSLANAT | LB_(Amygdala) | Cortical | 108 |
| FSLANAT | Area_CoS1_(CoS) | Cortical | 107 |
| FSLANAT | Right_Amygdala | Subcortical | 107 |
| FSLANAT | Area_FG4_(FusG) | Cortical | 106 |
| FSLANAT | Area_p24ab_(pACC) | Cortical | 103 |
| FSLANAT | Area_s24_(sACC) | Cortical | 103 |
| FSLANAT | Area_Ig1_(Insula) | Cortical | 102 |
| FSLANAT | CA1_(Hippocampus) | Cortical | 102 |
| FSLANAT | Subiculum_(Hippocampus) | Cortical | 101 |
| FSLANAT | Ch_123_(Basal_Forebrain) | Cortical | 100 |
| FSLANAT | Area_s32_(sACC) | Cortical | 99 |
| FSLANAT | Tuberculum_(Basal_Forebrain) | Cortical | 99 |
| FSLANAT | Area_OP2_(POperc) | Cortical | 98 |
| FSLANAT | Area_33_(ACC) | Cortical | 94 |
| FSLANAT | Area_Id6_(Insula) | Cortical | 94 |
| FSLANAT | Vermis_X | Cerebellum | 93 |
| FSLANAT | Area_Id4_(Insula) | Cortical | 92 |
| FSLANAT | Area_Id5_(Insula) | Cortical | 91 |
| FSLANAT | Area_Fo2_(OFC) | Cortical | 89 |
| FSLANAT | Area_Id9_(Insula) | Cortical | 89 |
| FSLANAT | Area_hOc4v_(LingG) | Cortical | 88 |
| FSLANAT | Area_Ig2_(Insula) | Cortical | 88 |
| FSLANAT | Area_Op7_(Frontal_Operculum) | Cortical | 87 |
| FSLANAT | Frontal-to-Temporal-II_(GapMap) | Cortical | 85 |
| FSLANAT | Right_Hippocampus | Subcortical | 85 |
| FSLANAT | Area_Id1_(Insula) | Cortical | 84 |
| FSLANAT | Area_Ig3_(Insula) | Cortical | 84 |
| FSLANAT | VP,_ventral_Pallidum_(Basal_Ganglia) | Cortical | 83 |
| FSLANAT | Area_Id3_(Insula) | Cortical | 82 |
| FSLANAT | Area_FG2_(FusG) | Cortical | 81 |
| FSLANAT | Frontal-to-Occipital_(GapMap) | Cortical | 81 |
| FSLANAT | Right_I-IV | Cerebellum | 81 |
| FSLANAT | Left_Amygdala | Subcortical | 80 |
| FSLANAT | Left_Thalamus | Subcortical | 80 |
| FSLANAT | Right_Thalamus | Subcortical | 80 |
| FSLANAT | CGL_(Metathalamus) | Cortical | 79 |
| FSLANAT | Left_I-IV | Cerebellum | 79 |
| FSLANAT | Area_Id2_(Insula) | Cortical | 78 |
| FSLANAT | Area_7M_(SPL) | Cortical | 77 |
| FSLANAT | Left_Lateral_Ventricle | Subcortical | 77 |
| FSLANAT | Area_Id7_(Insula) | Cortical | 75 |
| FSLANAT | IF_(Amygdala) | Cortical | 75 |
| FSLANAT | Area_Ia1_(Insula) | Cortical | 74 |
| FSLANAT | Area_Id8_(Insula) | Cortical | 74 |
| FSLANAT | HATA_(Hippocampus) | Cortical | 74 |
| FSLANAT | Area_hOc1_(V1,_17,_CalcS) | Cortical | 73 |
| FSLANAT | Area_OP3_(POperc) | Cortical | 73 |
| FSLANAT | Area_TE_1.1_(HESCHL) | Cortical | 70 |
| FSLANAT | Right_Lateral_Ventricle | Subcortical | 70 |
| FSLANAT | Area_Fo1_(OFC) | Cortical | 68 |
| FSLANAT | Area_hOc6_(POS) | Cortical | 68 |
| FSLANAT | Area_4p_(PreCG) | Cortical | 67 |
| FSLANAT | Area_Op5_(Frontal_Operculum) | Cortical | 67 |
| FSLANAT | Area_25_(sACC) | Cortical | 65 |
| FSLANAT | Area_hOc2_(V2,_18) | Cortical | 65 |
| FSLANAT | Area_hOc3v_(LingG) | Cortical | 64 |
| FSLANAT | Area_Op6_(Frontal_Operculum) | Cortical | 64 |
| FSLANAT | Area_p24c_(pACC) | Cortical | 64 |
| FSLANAT | Area_Ia2_(Insula) | Cortical | 63 |
| FSLANAT | Area_Ia3_(Insula) | Cortical | 62 |
| FSLANAT | Area_STS1_(STS) | Cortical | 61 |
| FSLANAT | Temporal-to-Parietal_(GapMap) | Cortical | 61 |
| FSLANAT | Area_5Ci_(SPL) | Cortical | 58 |
| FSLANAT | Area_Op8_(Frontal_Operculum) | Cortical | 58 |
| FSLANAT | Interposed_Nucleus_(Cerebellum) | Cortical | 57 |
| FSLANAT | Left_Hippocampus | Subcortical | 57 |
| FSLANAT | Area_5M_(SPL) | Cortical | 56 |
| FSLANAT | Area_OP1_(POperc) | Cortical | 56 |
| FSLANAT | Area_TE_1.2_(HESCHL) | Cortical | 56 |
| FSLANAT | Area_TI_(STG) | Cortical | 56 |
| FSLANAT | Area_TeI_(STG) | Cortical | 55 |
| FSLANAT | Area_Id10_(Insula) | Cortical | 54 |
| FSLANAT | CGM_(Metathalamus) | Cortical | 54 |
| FSLANAT | Right_X | Cerebellum | 54 |
| FSLANAT | Area_PFcm_(IPL) | Cortical | 53 |
| FSLANAT | Ch_4_(Basal_Forebrain) | Cortical | 53 |
| FSLANAT | Area_4a_(PreCG) | Cortical | 52 |
| FSLANAT | Area_TE_3_(STG) | Cortical | 52 |
| FSLANAT | Area_OP4_(POperc) | Cortical | 51 |
| FSLANAT | Left_X | Cerebellum | 51 |
| FSLANAT | STN_(Subthalamus) | Cortical | 51 |
| FSLANAT | Area_3a_(PostCG) | Cortical | 50 |
| FSLANAT | Area_6ma_(preSMA,_mesial_SFG) | Cortical | 50 |
| FSLANAT | Area_TE_1.0_(HESCHL) | Cortical | 49 |
| FSLANAT | Area_p32_(pACC) | Cortical | 48 |
| FSLANAT | Area_PGp_(IPL) | Cortical | 48 |
| FSLANAT | Frontal-to-Temporal-I_(GapMap) | Cortical | 48 |
| FSLANAT | Area_6mp_(SMA,_mesial_SFG) | Cortical | 47 |
| FSLANAT | Area_Fo3_(OFC) | Cortical | 47 |
| FSLANAT | Area_STS2_(STS) | Cortical | 47 |
| FSLANAT | Area_IFS2_(IFS) | Cortical | 46 |
| FSLANAT | Area_hIP7_(IPS) | Cortical | 45 |
| FSLANAT | Area_IFS4_(IFS) | Cortical | 45 |
| FSLANAT | Dorsal_Dentate_Nucleus_(Cerebellum) | Cortical | 45 |
| FSLANAT | Area_6d3_(SFS) | Cortical | 43 |
| FSLANAT | Area_TE_2.1_(STG) | Cortical | 43 |
| FSLANAT | Frontal-II_(GapMap) | Cortical | 43 |
| FSLANAT | Area_3b_(PostCG) | Cortical | 42 |
| FSLANAT | Area_hOc4la_(LOC) | Cortical | 42 |
| FSLANAT | Area_TE_2.2_(STG) | Cortical | 42 |
| FSLANAT | Area_44_(IFG) | Cortical | 41 |
| FSLANAT | Area_6d1_(PreCG) | Cortical | 41 |
| FSLANAT | Area_hIP4_(IPS) | Cortical | 41 |
| FSLANAT | Area_hOc3d_(Cuneus) | Cortical | 41 |
| FSLANAT | Area_1_(PostCG) | Cortical | 40 |
| FSLANAT | Area_Fo7_(OFC) | Cortical | 40 |
| FSLANAT | Area_hIP1_(IPS) | Cortical | 40 |
| FSLANAT | Area_hIP5_(IPS) | Cortical | 40 |
| FSLANAT | Area_6d2_(PreCG) | Cortical | 39 |
| FSLANAT | Area_Fp2_(FPole) | Cortical | 39 |
| FSLANAT | Area_PFt_(IPL) | Cortical | 39 |
| FSLANAT | Brain_Stem | Subcortical | 39 |
| FSLANAT | Area_hIP8_(IPS) | Cortical | 38 |
| FSLANAT | Area_IFJ2_(IFS,PreCS) | Cortical | 38 |
| FSLANAT | Area_IFS1_(IFS) | Cortical | 38 |
| FSLANAT | Area_PF_(IPL) | Cortical | 38 |
| FSLANAT | Area_2_(PostCS) | Cortical | 37 |
| FSLANAT | Area_8v1_(MFG) | Cortical | 37 |
| FSLANAT | Area_PGa_(IPL) | Cortical | 37 |
| FSLANAT | Area_45_(IFG) | Cortical | 36 |
| FSLANAT | Area_8d1_(SFG) | Cortical | 36 |
| FSLANAT | Area_Fo4_(OFC) | Cortical | 36 |
| FSLANAT | Area_IFJ1_(IFS,PreCS) | Cortical | 36 |
| FSLANAT | Area_Op9_(Frontal_Operculum) | Cortical | 36 |
| FSLANAT | Area_PFm_(IPL) | Cortical | 36 |
| FSLANAT | Area_PFop_(IPL) | Cortical | 36 |
| FSLANAT | Frontal-I_(GapMap) | Cortical | 36 |
| FSLANAT | Area_8v2_(MFG) | Cortical | 35 |
| FSLANAT | Area_Fo6_(OFC) | Cortical | 35 |
| FSLANAT | Area_hOc5_(LOC) | Cortical | 35 |
| FSLANAT | Area_TPJ_(STG/SMG) | Cortical | 35 |
| FSLANAT | Area_IFS3_(IFS) | Cortical | 34 |
| FSLANAT | Area_hIP3_(IPS) | Cortical | 33 |
| FSLANAT | Vermis_Crus_I | Cerebellum | 33 |
| FSLANAT | Area_hIP6_(IPS) | Cortical | 31 |
| FSLANAT | Area_hOc4lp_(LOC) | Cortical | 31 |
| FSLANAT | Area_hPO1_(POS) | Cortical | 31 |
| FSLANAT | Area_Fo5_(OFC) | Cortical | 30 |
| FSLANAT | Area_Fp1_(FPole) | Cortical | 30 |
| FSLANAT | Ventral_Dentate_Nucleus_(Cerebellum) | Cortical | 30 |
| FSLANAT | Area_7P_(SPL) | Cortical | 29 |
| FSLANAT | Area_5L_(SPL) | Cortical | 28 |
| FSLANAT | Area_8d2_(SFG) | Cortical | 28 |
| FSLANAT | Area_hIP2_(IPS) | Cortical | 28 |
| FSLANAT | Area_hOc4d_(Cuneus) | Cortical | 28 |
| FSLANAT | Area_MFG1_(MFG) | Cortical | 28 |
| FSLANAT | Area_7PC_(SPL) | Cortical | 27 |
| FSLANAT | Area_MFG2_(MFG) | Cortical | 27 |
| FSLANAT | Area_SFS2_(SFS) | Cortical | 26 |
| FSLANAT | Area_SFS1_(SFS) | Cortical | 25 |
| FSLANAT | Area_7A_(SPL) | Cortical | 23 |
| FSLANAT | Right_Pallidum | Subcortical | 23 |
| FSLANAT | Left_Pallidum | Subcortical | 22 |
| **Processing** | **ROI** | **ROI Location** | **Mean SumR2** |
| CAT12 Volume | Right_VIIb | Cerebellum | 980 |
| CAT12 Volume | MF_(Amygdala) | Cortical | 903 |
| CAT12 Volume | VTM_(Amygdala) | Cortical | 883 |
| CAT12 Volume | Left_VIIb | Cerebellum | 874 |
| CAT12 Volume | Right_VIIIa | Cerebellum | 858 |
| CAT12 Volume | Left_Crus_I | Cerebellum | 834 |
| CAT12 Volume | Right_Putamen | Subcortical | 830 |
| CAT12 Volume | Right_Crus_II | Cerebellum | 824 |
| CAT12 Volume | Vermis_VIIb | Cerebellum | 806 |
| CAT12 Volume | Left_Caudate | Subcortical | 803 |
| CAT12 Volume | Left_Crus_II | Cerebellum | 787 |
| CAT12 Volume | Right_Caudate | Subcortical | 776 |
| CAT12 Volume | Right_Crus_I | Cerebellum | 762 |
| CAT12 Volume | Area_Id5_(Insula) | Cortical | 752 |
| CAT12 Volume | Area_Id3_(Insula) | Cortical | 747 |
| CAT12 Volume | Medial_Accumbens,_ventral_Striatum_(Basal_Ganglia) | Cortical | 739 |
| CAT12 Volume | Right_VI | Cerebellum | 736 |
| CAT12 Volume | Vermis_IX | Cerebellum | 736 |
| CAT12 Volume | Left_VIIIa | Cerebellum | 734 |
| CAT12 Volume | Left_Putamen | Subcortical | 716 |
| CAT12 Volume | Area_Ig2_(Insula) | Cortical | 708 |
| CAT12 Volume | Fundus_of_Caudate_Nucleus,_ventral_Striatum_(Basal_Ganglia) | Cortical | 700 |
| CAT12 Volume | Fundus_of_Putamen,_Ventral_Striatum_(Basal_Ganglia) | Cortical | 684 |
| CAT12 Volume | IF_(Amygdala) | Cortical | 672 |
| CAT12 Volume | Lateral_Accumbens,_ventral_Striatum_(Basal_Ganglia) | Cortical | 664 |
| CAT12 Volume | Right_VIIIb | Cerebellum | 660 |
| CAT12 Volume | Area_Id1_(Insula) | Cortical | 659 |
| CAT12 Volume | Area_Ig1_(Insula) | Cortical | 654 |
| CAT12 Volume | Left_VI | Cerebellum | 653 |
| CAT12 Volume | Area_Id2_(Insula) | Cortical | 650 |
| CAT12 Volume | Right_Thalamus | Subcortical | 644 |
| CAT12 Volume | Area_Id6_(Insula) | Cortical | 637 |
| CAT12 Volume | Area_Id4_(Insula) | Cortical | 632 |
| CAT12 Volume | Left_Thalamus | Subcortical | 625 |
| CAT12 Volume | Area_Ig3_(Insula) | Cortical | 622 |
| CAT12 Volume | Area_OP2_(POperc) | Cortical | 620 |
| CAT12 Volume | LB_(Amygdala) | Cortical | 592 |
| CAT12 Volume | Left_IX | Cerebellum | 590 |
| CAT12 Volume | SF_(Amygdala) | Cortical | 589 |
| CAT12 Volume | Left_VIIIb | Cerebellum | 588 |
| CAT12 Volume | CM_(Amygdala) | Cortical | 577 |
| CAT12 Volume | CA3_(Hippocampus) | Cortical | 576 |
| CAT12 Volume | Vermis_VIIIb | Cerebellum | 568 |
| CAT12 Volume | Vermis_Crus_II | Cerebellum | 567 |
| CAT12 Volume | Area_TE_1.1_(HESCHL) | Cortical | 553 |
| CAT12 Volume | Vermis_VIIIa | Cerebellum | 544 |
| CAT12 Volume | Right_IX | Cerebellum | 543 |
| CAT12 Volume | Area_s24_(sACC) | Cortical | 536 |
| CAT12 Volume | Left_Amygdala | Subcortical | 535 |
| CAT12 Volume | Area_Ia1_(Insula) | Cortical | 533 |
| CAT12 Volume | Area_Ia2_(Insula) | Cortical | 509 |
| CAT12 Volume | Area_s32_(sACC) | Cortical | 508 |
| CAT12 Volume | DG_(Hippocampus) | Cortical | 508 |
| CAT12 Volume | Area_Op7_(Frontal_Operculum) | Cortical | 507 |
| CAT12 Volume | Area_Fo2_(OFC) | Cortical | 505 |
| CAT12 Volume | Right_Lateral_Ventricle | Subcortical | 500 |
| CAT12 Volume | CA2_(Hippocampus) | Cortical | 498 |
| CAT12 Volume | Right_V | Cerebellum | 489 |
| CAT12 Volume | Terminal_islands_(Basal_Forebrain) | Cortical | 481 |
| CAT12 Volume | Area_OP3_(POperc) | Cortical | 475 |
| CAT12 Volume | VP,_ventral_Pallidum_(Basal_Ganglia) | Cortical | 475 |
| CAT12 Volume | Area_Ph2_(PhG) | Cortical | 466 |
| CAT12 Volume | Left_V | Cerebellum | 463 |
| CAT12 Volume | Area_Id7_(Insula) | Cortical | 456 |
| CAT12 Volume | Area_Id9_(Insula) | Cortical | 449 |
| CAT12 Volume | Area_Ph3_(PhG) | Cortical | 447 |
| CAT12 Volume | Area_25_(sACC) | Cortical | 440 |
| CAT12 Volume | Tuberculum_(Basal_Forebrain) | Cortical | 425 |
| CAT12 Volume | Vermis_X | Cerebellum | 418 |
| CAT12 Volume | Area_Op5_(Frontal_Operculum) | Cortical | 415 |
| CAT12 Volume | HATA_(Hippocampus) | Cortical | 414 |
| CAT12 Volume | Area_Id8_(Insula) | Cortical | 412 |
| CAT12 Volume | Right_Pallidum | Subcortical | 412 |
| CAT12 Volume | Area_TE_1.0_(HESCHL) | Cortical | 411 |
| CAT12 Volume | Area_Fo1_(OFC) | Cortical | 409 |
| CAT12 Volume | Area_CoS1_(CoS) | Cortical | 406 |
| CAT12 Volume | Area_FG3_(FusG) | Cortical | 400 |
| CAT12 Volume | Frontal-to-Temporal-II_(GapMap) | Cortical | 398 |
| CAT12 Volume | CA1_(Hippocampus) | Cortical | 392 |
| CAT12 Volume | Area_p24ab_(pACC) | Cortical | 388 |
| CAT12 Volume | Fastigial_Nucleus_(Cerebellum) | Cortical | 382 |
| CAT12 Volume | Area_PFcm_(IPL) | Cortical | 377 |
| CAT12 Volume | Area_TI_(STG) | Cortical | 377 |
| CAT12 Volume | Area_33_(ACC) | Cortical | 370 |
| CAT12 Volume | Area_OP1_(POperc) | Cortical | 369 |
| CAT12 Volume | Entorhinal_Cortex | Cortical | 367 |
| CAT12 Volume | Right_I-IV | Cerebellum | 367 |
| CAT12 Volume | Subiculum_(Hippocampus) | Cortical | 366 |
| CAT12 Volume | Left_I-IV | Cerebellum | 360 |
| CAT12 Volume | Area_p24c_(pACC) | Cortical | 353 |
| CAT12 Volume | Area_TeI_(STG) | Cortical | 352 |
| CAT12 Volume | Left_Lateral_Ventricle | Subcortical | 350 |
| CAT12 Volume | Vermis_VI | Cerebellum | 349 |
| CAT12 Volume | Area_Ia3_(Insula) | Cortical | 345 |
| CAT12 Volume | Area_Op8_(Frontal_Operculum) | Cortical | 340 |
| CAT12 Volume | Ch_123_(Basal_Forebrain) | Cortical | 333 |
| CAT12 Volume | Right_X | Cerebellum | 329 |
| CAT12 Volume | BST_(Bed_Nucleus) | Cortical | 323 |
| CAT12 Volume | Area_Ph1_(PhG) | Cortical | 320 |
| CAT12 Volume | HC-Transsubiculum_(Hippocampus) | Cortical | 319 |
| CAT12 Volume | Left_X | Cerebellum | 312 |
| CAT12 Volume | Area_TE_2.2_(STG) | Cortical | 308 |
| CAT12 Volume | Area_Id10_(Insula) | Cortical | 307 |
| CAT12 Volume | Right_Hippocampus | Subcortical | 307 |
| CAT12 Volume | Area_FG1_(FusG) | Cortical | 302 |
| CAT12 Volume | Frontal-to-Occipital_(GapMap) | Cortical | 300 |
| CAT12 Volume | Area_FG4_(FusG) | Cortical | 299 |
| CAT12 Volume | Ch_4_(Basal_Forebrain) | Cortical | 296 |
| CAT12 Volume | Area_TE_1.2_(HESCHL) | Cortical | 295 |
| CAT12 Volume | Area_7M_(SPL) | Cortical | 289 |
| CAT12 Volume | Area_STS1_(STS) | Cortical | 289 |
| CAT12 Volume | Area_Op6_(Frontal_Operculum) | Cortical | 286 |
| CAT12 Volume | Ventral_Dentate_Nucleus_(Cerebellum) | Cortical | 284 |
| CAT12 Volume | CGM_(Metathalamus) | Cortical | 283 |
| CAT12 Volume | Dorsal_Dentate_Nucleus_(Cerebellum) | Cortical | 279 |
| CAT12 Volume | Area_Fo3_(OFC) | Cortical | 274 |
| CAT12 Volume | Area_p32_(pACC) | Cortical | 270 |
| CAT12 Volume | Area_Fo4_(OFC) | Cortical | 269 |
| CAT12 Volume | Area_OP4_(POperc) | Cortical | 269 |
| CAT12 Volume | Area_TE_3_(STG) | Cortical | 267 |
| CAT12 Volume | Frontal-to-Temporal-I_(GapMap) | Cortical | 259 |
| CAT12 Volume | Temporal-to-Parietal_(GapMap) | Cortical | 258 |
| CAT12 Volume | Area_Fo7_(OFC) | Cortical | 257 |
| CAT12 Volume | Area_TE_2.1_(STG) | Cortical | 257 |
| CAT12 Volume | Area_5M_(SPL) | Cortical | 254 |
| CAT12 Volume | Area_hOc6_(POS) | Cortical | 253 |
| CAT12 Volume | Vermis_Crus_I | Cerebellum | 251 |
| CAT12 Volume | Area_Op9_(Frontal_Operculum) | Cortical | 249 |
| CAT12 Volume | Area_Fo6_(OFC) | Cortical | 247 |
| CAT12 Volume | Area_FG2_(FusG) | Cortical | 244 |
| CAT12 Volume | Area_STS2_(STS) | Cortical | 244 |
| CAT12 Volume | Area_IFS2_(IFS) | Cortical | 242 |
| CAT12 Volume | Area_4a_(PreCG) | Cortical | 238 |
| CAT12 Volume | Area_4p_(PreCG) | Cortical | 230 |
| CAT12 Volume | Area_Fp2_(FPole) | Cortical | 230 |
| CAT12 Volume | Area_Fp1_(FPole) | Cortical | 228 |
| CAT12 Volume | Area_hIP5_(IPS) | Cortical | 227 |
| CAT12 Volume | CGL_(Metathalamus) | Cortical | 227 |
| CAT12 Volume | Interposed_Nucleus_(Cerebellum) | Cortical | 226 |
| CAT12 Volume | Area_3a_(PostCG) | Cortical | 224 |
| CAT12 Volume | Area_5Ci_(SPL) | Cortical | 223 |
| CAT12 Volume | Area_hOc1_(V1,_17,_CalcS) | Cortical | 219 |
| CAT12 Volume | Area_hOc4v_(LingG) | Cortical | 219 |
| CAT12 Volume | Area_6ma_(preSMA,_mesial_SFG) | Cortical | 217 |
| CAT12 Volume | Area_PFop_(IPL) | Cortical | 211 |
| CAT12 Volume | Area_PFt_(IPL) | Cortical | 211 |
| CAT12 Volume | Area_Fo5_(OFC) | Cortical | 210 |
| CAT12 Volume | Area_2_(PostCS) | Cortical | 208 |
| CAT12 Volume | Area_PF_(IPL) | Cortical | 204 |
| CAT12 Volume | STN_(Subthalamus) | Cortical | 204 |
| CAT12 Volume | Area_6mp_(SMA,_mesial_SFG) | Cortical | 201 |
| CAT12 Volume | Area_hIP8_(IPS) | Cortical | 201 |
| CAT12 Volume | Area_3b_(PostCG) | Cortical | 199 |
| CAT12 Volume | Area_8d1_(SFG) | Cortical | 199 |
| CAT12 Volume | Area_IFS4_(IFS) | Cortical | 197 |
| CAT12 Volume | Frontal-I_(GapMap) | Cortical | 197 |
| CAT12 Volume | Area_IFS1_(IFS) | Cortical | 196 |
| CAT12 Volume | Area_8d2_(SFG) | Cortical | 195 |
| CAT12 Volume | Area_SFS1_(SFS) | Cortical | 194 |
| CAT12 Volume | Frontal-II_(GapMap) | Cortical | 194 |
| CAT12 Volume | Area_1_(PostCG) | Cortical | 192 |
| CAT12 Volume | Area_45_(IFG) | Cortical | 192 |
| CAT12 Volume | Area_hOc2_(V2,_18) | Cortical | 191 |
| CAT12 Volume | Area_TPJ_(STG/SMG) | Cortical | 190 |
| CAT12 Volume | Brain_Stem | Subcortical | 189 |
| CAT12 Volume | Area_44_(IFG) | Cortical | 188 |
| CAT12 Volume | Area_6d1_(PreCG) | Cortical | 184 |
| CAT12 Volume | Area_SFS2_(SFS) | Cortical | 184 |
| CAT12 Volume | Area_6d2_(PreCG) | Cortical | 183 |
| CAT12 Volume | Area_6d3_(SFS) | Cortical | 183 |
| CAT12 Volume | Left_Pallidum | Subcortical | 181 |
| CAT12 Volume | Area_MFG1_(MFG) | Cortical | 178 |
| CAT12 Volume | Area_IFJ2_(IFS,PreCS) | Cortical | 176 |
| CAT12 Volume | Area_hOc3v_(LingG) | Cortical | 174 |
| CAT12 Volume | Area_hPO1_(POS) | Cortical | 172 |
| CAT12 Volume | Area_PGp_(IPL) | Cortical | 172 |
| CAT12 Volume | Area_hIP7_(IPS) | Cortical | 171 |
| CAT12 Volume | Area_hOc4la_(LOC) | Cortical | 168 |
| CAT12 Volume | Area_8v1_(MFG) | Cortical | 165 |
| CAT12 Volume | Area_hIP4_(IPS) | Cortical | 164 |
| CAT12 Volume | Area_PGa_(IPL) | Cortical | 164 |
| CAT12 Volume | Area_IFJ1_(IFS,PreCS) | Cortical | 163 |
| CAT12 Volume | Area_7P_(SPL) | Cortical | 162 |
| CAT12 Volume | Area_hOc5_(LOC) | Cortical | 162 |
| CAT12 Volume | Area_PFm_(IPL) | Cortical | 161 |
| CAT12 Volume | Area_hIP2_(IPS) | Cortical | 156 |
| CAT12 Volume | Area_IFS3_(IFS) | Cortical | 156 |
| CAT12 Volume | Right_Amygdala | Subcortical | 156 |
| CAT12 Volume | Area_hIP6_(IPS) | Cortical | 154 |
| CAT12 Volume | Area_hIP3_(IPS) | Cortical | 153 |
| CAT12 Volume | Area_hOc3d_(Cuneus) | Cortical | 153 |
| CAT12 Volume | Left_Hippocampus | Subcortical | 153 |
| CAT12 Volume | Area_5L_(SPL) | Cortical | 151 |
| CAT12 Volume | Area_hIP1_(IPS) | Cortical | 150 |
| CAT12 Volume | Area_8v2_(MFG) | Cortical | 144 |
| CAT12 Volume | Area_MFG2_(MFG) | Cortical | 144 |
| CAT12 Volume | Area_7A_(SPL) | Cortical | 131 |
| CAT12 Volume | Area_hOc4d_(Cuneus) | Cortical | 127 |
| CAT12 Volume | Area_7PC_(SPL) | Cortical | 124 |
| CAT12 Volume | Area_hOc4lp_(LOC) | Cortical | 124 |
| **Processing** | **ROI** | **ROI Location** | **Mean SumR2** |
| CAT12 Surface | Area_Ig1_(Insula) | Cortical | 3207 |
| CAT12 Surface | Dorsal_Dentate_Nucleus_(Cerebellum) | Cortical | 2915 |
| CAT12 Surface | Area_hOc4la_(LOC) | Cortical | 2906 |
| CAT12 Surface | Area_TE_1.1_(HESCHL) | Cortical | 2813 |
| CAT12 Surface | Interposed_Nucleus_(Cerebellum) | Cortical | 2656 |
| CAT12 Surface | Area_Ig3_(Insula) | Cortical | 2638 |
| CAT12 Surface | Area_p24c_(pACC) | Cortical | 2635 |
| CAT12 Surface | Area_3a_(PostCG) | Cortical | 2596 |
| CAT12 Surface | Area_IFS4_(IFS) | Cortical | 2575 |
| CAT12 Surface | Area_Id1_(Insula) | Cortical | 2519 |
| CAT12 Surface | Area_IFS2_(IFS) | Cortical | 2502 |
| CAT12 Surface | Ventral_Dentate_Nucleus_(Cerebellum) | Cortical | 2454 |
| CAT12 Surface | Area_TE_2.1_(STG) | Cortical | 2439 |
| CAT12 Surface | Area_hOc4lp_(LOC) | Cortical | 2411 |
| CAT12 Surface | Area_IFJ1_(IFS,PreCS) | Cortical | 2343 |
| CAT12 Surface | Area_3b_(PostCG) | Cortical | 2309 |
| CAT12 Surface | Area_4p_(PreCG) | Cortical | 2260 |
| CAT12 Surface | Area_hOc3d_(Cuneus) | Cortical | 2178 |
| CAT12 Surface | Area_OP1_(POperc) | Cortical | 2167 |
| CAT12 Surface | Area_IFS1_(IFS) | Cortical | 2142 |
| CAT12 Surface | Fastigial_Nucleus_(Cerebellum) | Cortical | 2137 |
| CAT12 Surface | Area_hOc4d_(Cuneus) | Cortical | 2104 |
| CAT12 Surface | Area_TeI_(STG) | Cortical | 2074 |
| CAT12 Surface | Area_TE_1.2_(HESCHL) | Cortical | 2063 |
| CAT12 Surface | CA3_(Hippocampus) | Cortical | 2036 |
| CAT12 Surface | Ch_123_(Basal_Forebrain) | Cortical | 2035 |
| CAT12 Surface | Area_Op9_(Frontal_Operculum) | Cortical | 2034 |
| CAT12 Surface | VTM_(Amygdala) | Cortical | 2016 |
| CAT12 Surface | Area_1_(PostCG) | Cortical | 2005 |
| CAT12 Surface | Area_TE_1.0_(HESCHL) | Cortical | 2001 |
| CAT12 Surface | Area_Fp1_(FPole) | Cortical | 1994 |
| CAT12 Surface | Area_p24ab_(pACC) | Cortical | 1990 |
| CAT12 Surface | Entorhinal_Cortex | Cortical | 1989 |
| CAT12 Surface | Area_TPJ_(STG/SMG) | Cortical | 1973 |
| CAT12 Surface | Area_hIP2_(IPS) | Cortical | 1964 |
| CAT12 Surface | Area_6d2_(PreCG) | Cortical | 1947 |
| CAT12 Surface | Area_PFcm_(IPL) | Cortical | 1947 |
| CAT12 Surface | Area_TE_2.2_(STG) | Cortical | 1931 |
| CAT12 Surface | Area_7P_(SPL) | Cortical | 1928 |
| CAT12 Surface | Area_2_(PostCS) | Cortical | 1898 |
| CAT12 Surface | Area_IFJ2_(IFS,PreCS) | Cortical | 1892 |
| CAT12 Surface | Area_TE_3_(STG) | Cortical | 1868 |
| CAT12 Surface | Area_Fp2_(FPole) | Cortical | 1863 |
| CAT12 Surface | Area_7A_(SPL) | Cortical | 1859 |
| CAT12 Surface | Area_hIP1_(IPS) | Cortical | 1855 |
| CAT12 Surface | Area_FG1_(FusG) | Cortical | 1838 |
| CAT12 Surface | Area_5L_(SPL) | Cortical | 1837 |
| CAT12 Surface | Area_OP3_(POperc) | Cortical | 1820 |
| CAT12 Surface | Area_4a_(PreCG) | Cortical | 1799 |
| CAT12 Surface | Subiculum_(Hippocampus) | Cortical | 1780 |
| CAT12 Surface | Area_45_(IFG) | Cortical | 1778 |
| CAT12 Surface | Area_44_(IFG) | Cortical | 1773 |
| CAT12 Surface | Area_6d3_(SFS) | Cortical | 1757 |
| CAT12 Surface | Area_6mp_(SMA,_mesial_SFG) | Cortical | 1749 |
| CAT12 Surface | CA2_(Hippocampus) | Cortical | 1733 |
| CAT12 Surface | Area_6ma_(preSMA,_mesial_SFG) | Cortical | 1728 |
| CAT12 Surface | Area_PFt_(IPL) | Cortical | 1687 |
| CAT12 Surface | Area_TI_(STG) | Cortical | 1669 |
| CAT12 Surface | Area_OP2_(POperc) | Cortical | 1659 |
| CAT12 Surface | Area_IFS3_(IFS) | Cortical | 1644 |
| CAT12 Surface | DG_(Hippocampus) | Cortical | 1603 |
| CAT12 Surface | Area_7M_(SPL) | Cortical | 1600 |
| CAT12 Surface | Area_FG2_(FusG) | Cortical | 1597 |
| CAT12 Surface | MF_(Amygdala) | Cortical | 1582 |
| CAT12 Surface | Area_6d1_(PreCG) | Cortical | 1577 |
| CAT12 Surface | HC-Transsubiculum_(Hippocampus) | Cortical | 1558 |
| CAT12 Surface | Area_Ig2_(Insula) | Cortical | 1547 |
| CAT12 Surface | Area_8v1_(MFG) | Cortical | 1473 |
| CAT12 Surface | Area_FG4_(FusG) | Cortical | 1471 |
| CAT12 Surface | Area_hIP7_(IPS) | Cortical | 1378 |
| CAT12 Surface | Area_hIP5_(IPS) | Cortical | 1355 |
| CAT12 Surface | Area_hIP4_(IPS) | Cortical | 1343 |
| CAT12 Surface | Area_OP4_(POperc) | Cortical | 1325 |
| CAT12 Surface | Area_Id5_(Insula) | Cortical | 1295 |
| CAT12 Surface | Area_FG3_(FusG) | Cortical | 1290 |
| CAT12 Surface | Area_hPO1_(POS) | Cortical | 1260 |
| CAT12 Surface | HATA_(Hippocampus) | Cortical | 1258 |
| CAT12 Surface | IF_(Amygdala) | Cortical | 1249 |
| CAT12 Surface | Area_Fo4_(OFC) | Cortical | 1221 |
| CAT12 Surface | Area_PGa_(IPL) | Cortical | 1193 |
| CAT12 Surface | CM_(Amygdala) | Cortical | 1180 |
| CAT12 Surface | Tuberculum_(Basal_Forebrain) | Cortical | 1180 |
| CAT12 Surface | Area_hOc5_(LOC) | Cortical | 1108 |
| CAT12 Surface | Area_5M_(SPL) | Cortical | 1086 |
| CAT12 Surface | Area_PGp_(IPL) | Cortical | 1084 |
| CAT12 Surface | Area_p32_(pACC) | Cortical | 1075 |
| CAT12 Surface | Area_STS2_(STS) | Cortical | 1072 |
| CAT12 Surface | Area_Ia1_(Insula) | Cortical | 1061 |
| CAT12 Surface | Area_PFop_(IPL) | Cortical | 1060 |
| CAT12 Surface | Area_hIP8_(IPS) | Cortical | 1050 |
| CAT12 Surface | Area_hOc1_(V1,_17,_CalcS) | Cortical | 1042 |
| CAT12 Surface | Area_PF_(IPL) | Cortical | 1024 |
| CAT12 Surface | Area_Id7_(Insula) | Cortical | 1001 |
| CAT12 Surface | Area_PFm_(IPL) | Cortical | 998 |
| CAT12 Surface | Area_Id2_(Insula) | Cortical | 981 |
| CAT12 Surface | Area_Op8_(Frontal_Operculum) | Cortical | 981 |
| CAT12 Surface | Area_hOc3v_(LingG) | Cortical | 953 |
| CAT12 Surface | Area_Id4_(Insula) | Cortical | 938 |
| CAT12 Surface | Area_25_(sACC) | Cortical | 916 |
| CAT12 Surface | Area_STS1_(STS) | Cortical | 894 |
| CAT12 Surface | Terminal_islands_(Basal_Forebrain) | Cortical | 886 |
| CAT12 Surface | CA1_(Hippocampus) | Cortical | 878 |
| CAT12 Surface | Area_5Ci_(SPL) | Cortical | 877 |
| CAT12 Surface | Ch_4_(Basal_Forebrain) | Cortical | 872 |
| CAT12 Surface | LB_(Amygdala) | Cortical | 868 |
| CAT12 Surface | Area_8d1_(SFG) | Cortical | 850 |
| CAT12 Surface | Area_Id3_(Insula) | Cortical | 833 |
| CAT12 Surface | Area_Fo7_(OFC) | Cortical | 781 |
| CAT12 Surface | Area_hIP6_(IPS) | Cortical | 772 |
| CAT12 Surface | Area_hOc6_(POS) | Cortical | 758 |
| CAT12 Surface | Area_hOc2_(V2,_18) | Cortical | 718 |
| CAT12 Surface | Area_8d2_(SFG) | Cortical | 711 |
| CAT12 Surface | Area_Fo3_(OFC) | Cortical | 693 |
| CAT12 Surface | Area_Fo6_(OFC) | Cortical | 685 |
| CAT12 Surface | Area_8v2_(MFG) | Cortical | 679 |
| CAT12 Surface | Area_Id6_(Insula) | Cortical | 676 |
| CAT12 Surface | SF_(Amygdala) | Cortical | 615 |
| CAT12 Surface | Area_hOc4v_(LingG) | Cortical | 563 |
| CAT12 Surface | Area_Fo1_(OFC) | Cortical | 468 |
| CAT12 Surface | Area_Fo5_(OFC) | Cortical | 462 |
| CAT12 Surface | Area_s32_(sACC) | Cortical | 458 |
| CAT12 Surface | Area_s24_(sACC) | Cortical | 449 |
| CAT12 Surface | Area_33_(ACC) | Cortical | 414 |
| CAT12 Surface | Area_SFS1_(SFS) | Cortical | 343 |
| CAT12 Surface | Area_Fo2_(OFC) | Cortical | 342 |
| CAT12 Surface | Area_7PC_(SPL) | Cortical | 308 |
| CAT12 Surface | Area_hIP3_(IPS) | Cortical | 280 |
| **Processing** | **ROI** | **ROI Location** | **Mean SumR2** |
| FreeSurfer Thickness | Area_8d1_(SFG) | Cortical | 323 |
| FreeSurfer Thickness | Area_Ig1_(Insula) | Cortical | 313 |
| FreeSurfer Thickness | Area_Fo5_(OFC) | Cortical | 304 |
| FreeSurfer Thickness | Area_hOc4lp_(LOC) | Cortical | 280 |
| FreeSurfer Thickness | Area_Id1_(Insula) | Cortical | 280 |
| FreeSurfer Thickness | Dorsal_Dentate_Nucleus_(Cerebellum) | Cortical | 278 |
| FreeSurfer Thickness | Area_hOc4la_(LOC) | Cortical | 259 |
| FreeSurfer Thickness | Area_TE_1.1_(HESCHL) | Cortical | 248 |
| FreeSurfer Thickness | Ventral_Dentate_Nucleus_(Cerebellum) | Cortical | 248 |
| FreeSurfer Thickness | CA2_(Hippocampus) | Cortical | 240 |
| FreeSurfer Thickness | Area_TI_(STG) | Cortical | 239 |
| FreeSurfer Thickness | Area_TPJ_(STG/SMG) | Cortical | 237 |
| FreeSurfer Thickness | Area_PFt_(IPL) | Cortical | 235 |
| FreeSurfer Thickness | Area_hOc1_(V1,_17,_CalcS) | Cortical | 233 |
| FreeSurfer Thickness | Area_PF_(IPL) | Cortical | 228 |
| FreeSurfer Thickness | Area_Fo7_(OFC) | Cortical | 227 |
| FreeSurfer Thickness | Area_hOc3d_(Cuneus) | Cortical | 226 |
| FreeSurfer Thickness | CA3_(Hippocampus) | Cortical | 225 |
| FreeSurfer Thickness | Area_6ma_(preSMA,_mesial_SFG) | Cortical | 224 |
| FreeSurfer Thickness | Area_TE_2.1_(STG) | Cortical | 223 |
| FreeSurfer Thickness | Area_IFS4_(IFS) | Cortical | 221 |
| FreeSurfer Thickness | Subiculum_(Hippocampus) | Cortical | 220 |
| FreeSurfer Thickness | Area_OP3_(POperc) | Cortical | 219 |
| FreeSurfer Thickness | Area_Op9_(Frontal_Operculum) | Cortical | 219 |
| FreeSurfer Thickness | Area_PFcm_(IPL) | Cortical | 218 |
| FreeSurfer Thickness | Area_3b_(PostCG) | Cortical | 216 |
| FreeSurfer Thickness | Area_44_(IFG) | Cortical | 216 |
| FreeSurfer Thickness | Area_Ig3_(Insula) | Cortical | 214 |
| FreeSurfer Thickness | Area_33_(ACC) | Cortical | 212 |
| FreeSurfer Thickness | Area_6d3_(SFS) | Cortical | 212 |
| FreeSurfer Thickness | Interposed_Nucleus_(Cerebellum) | Cortical | 207 |
| FreeSurfer Thickness | Area_OP1_(POperc) | Cortical | 206 |
| FreeSurfer Thickness | Area_4p_(PreCG) | Cortical | 205 |
| FreeSurfer Thickness | Area_6d2_(PreCG) | Cortical | 205 |
| FreeSurfer Thickness | Area_Id3_(Insula) | Cortical | 205 |
| FreeSurfer Thickness | Area_IFJ2_(IFS,PreCS) | Cortical | 203 |
| FreeSurfer Thickness | Area_7A_(SPL) | Cortical | 199 |
| FreeSurfer Thickness | Area_TeI_(STG) | Cortical | 199 |
| FreeSurfer Thickness | Area_TE_2.2_(STG) | Cortical | 198 |
| FreeSurfer Thickness | Area_hIP1_(IPS) | Cortical | 193 |
| FreeSurfer Thickness | Area_Ig2_(Insula) | Cortical | 193 |
| FreeSurfer Thickness | Area_OP4_(POperc) | Cortical | 193 |
| FreeSurfer Thickness | Area_IFS2_(IFS) | Cortical | 191 |
| FreeSurfer Thickness | Area_3a_(PostCG) | Cortical | 190 |
| FreeSurfer Thickness | Area_1_(PostCG) | Cortical | 189 |
| FreeSurfer Thickness | Area_FG1_(FusG) | Cortical | 187 |
| FreeSurfer Thickness | DG_(Hippocampus) | Cortical | 186 |
| FreeSurfer Thickness | Area_hOc4d_(Cuneus) | Cortical | 184 |
| FreeSurfer Thickness | Area_TE_1.2_(HESCHL) | Cortical | 183 |
| FreeSurfer Thickness | Area_6d1_(PreCG) | Cortical | 182 |
| FreeSurfer Thickness | Area_hIP6_(IPS) | Cortical | 182 |
| FreeSurfer Thickness | Area_hOc2_(V2,_18) | Cortical | 182 |
| FreeSurfer Thickness | Area_Fo6_(OFC) | Cortical | 181 |
| FreeSurfer Thickness | Area_IFS1_(IFS) | Cortical | 181 |
| FreeSurfer Thickness | Area_p24c_(pACC) | Cortical | 181 |
| FreeSurfer Thickness | Area_Id2_(Insula) | Cortical | 180 |
| FreeSurfer Thickness | Area_Id5_(Insula) | Cortical | 177 |
| FreeSurfer Thickness | Area_OP2_(POperc) | Cortical | 174 |
| FreeSurfer Thickness | HC-Transsubiculum_(Hippocampus) | Cortical | 174 |
| FreeSurfer Thickness | Tuberculum_(Basal_Forebrain) | Cortical | 173 |
| FreeSurfer Thickness | Area_Fp2_(FPole) | Cortical | 171 |
| FreeSurfer Thickness | Area_hIP7_(IPS) | Cortical | 168 |
| FreeSurfer Thickness | Ch_123_(Basal_Forebrain) | Cortical | 167 |
| FreeSurfer Thickness | Area_2_(PostCS) | Cortical | 166 |
| FreeSurfer Thickness | Area_45_(IFG) | Cortical | 166 |
| FreeSurfer Thickness | Area_FG2_(FusG) | Cortical | 166 |
| FreeSurfer Thickness | Area_Id6_(Insula) | Cortical | 166 |
| FreeSurfer Thickness | Area_PGa_(IPL) | Cortical | 166 |
| FreeSurfer Thickness | Fastigial_Nucleus_(Cerebellum) | Cortical | 163 |
| FreeSurfer Thickness | Area_4a_(PreCG) | Cortical | 162 |
| FreeSurfer Thickness | Area_PFop_(IPL) | Cortical | 162 |
| FreeSurfer Thickness | Area_Ia1_(Insula) | Cortical | 161 |
| FreeSurfer Thickness | Area_IFS3_(IFS) | Cortical | 160 |
| FreeSurfer Thickness | Area_TE_3_(STG) | Cortical | 159 |
| FreeSurfer Thickness | CM_(Amygdala) | Cortical | 159 |
| FreeSurfer Thickness | Area_p24ab_(pACC) | Cortical | 158 |
| FreeSurfer Thickness | Area_Fp1_(FPole) | Cortical | 156 |
| FreeSurfer Thickness | LB_(Amygdala) | Cortical | 156 |
| FreeSurfer Thickness | Area_hOc6_(POS) | Cortical | 155 |
| FreeSurfer Thickness | Area_Id4_(Insula) | Cortical | 155 |
| FreeSurfer Thickness | Area_Fo4_(OFC) | Cortical | 154 |
| FreeSurfer Thickness | Area_IFJ1_(IFS,PreCS) | Cortical | 153 |
| FreeSurfer Thickness | Area_PGp_(IPL) | Cortical | 153 |
| FreeSurfer Thickness | Area_6mp_(SMA,_mesial_SFG) | Cortical | 152 |
| FreeSurfer Thickness | Area_7M_(SPL) | Cortical | 151 |
| FreeSurfer Thickness | IF_(Amygdala) | Cortical | 149 |
| FreeSurfer Thickness | Area_7P_(SPL) | Cortical | 148 |
| FreeSurfer Thickness | Area_hOc3v_(LingG) | Cortical | 145 |
| FreeSurfer Thickness | Area_hOc5_(LOC) | Cortical | 145 |
| FreeSurfer Thickness | Terminal_islands_(Basal_Forebrain) | Cortical | 144 |
| FreeSurfer Thickness | Area_8d2_(SFG) | Cortical | 143 |
| FreeSurfer Thickness | Area_FG4_(FusG) | Cortical | 141 |
| FreeSurfer Thickness | Area_STS2_(STS) | Cortical | 141 |
| FreeSurfer Thickness | Area_8v1_(MFG) | Cortical | 139 |
| FreeSurfer Thickness | Area_hIP8_(IPS) | Cortical | 137 |
| FreeSurfer Thickness | HATA_(Hippocampus) | Cortical | 132 |
| FreeSurfer Thickness | MF_(Amygdala) | Cortical | 132 |
| FreeSurfer Thickness | VTM_(Amygdala) | Cortical | 132 |
| FreeSurfer Thickness | Area_7PC_(SPL) | Cortical | 128 |
| FreeSurfer Thickness | Area_hIP2_(IPS) | Cortical | 126 |
| FreeSurfer Thickness | Area_5M_(SPL) | Cortical | 120 |
| FreeSurfer Thickness | Area_FG3_(FusG) | Cortical | 120 |
| FreeSurfer Thickness | Area_PFm_(IPL) | Cortical | 120 |
| FreeSurfer Thickness | Area_hOc4v_(LingG) | Cortical | 115 |
| FreeSurfer Thickness | Area_25_(sACC) | Cortical | 111 |
| FreeSurfer Thickness | Area_Id7_(Insula) | Cortical | 111 |
| FreeSurfer Thickness | Area_s24_(sACC) | Cortical | 111 |
| FreeSurfer Thickness | Entorhinal_Cortex | Cortical | 110 |
| FreeSurfer Thickness | CA1_(Hippocampus) | Cortical | 109 |
| FreeSurfer Thickness | Area_5L_(SPL) | Cortical | 108 |
| FreeSurfer Thickness | Area_TE_1.0_(HESCHL) | Cortical | 106 |
| FreeSurfer Thickness | Area_hPO1_(POS) | Cortical | 105 |
| FreeSurfer Thickness | Area_hIP5_(IPS) | Cortical | 104 |
| FreeSurfer Thickness | Area_Fo3_(OFC) | Cortical | 103 |
| FreeSurfer Thickness | Area_p32_(pACC) | Cortical | 101 |
| FreeSurfer Thickness | Area_Fo1_(OFC) | Cortical | 99 |
| FreeSurfer Thickness | Area_Fo2_(OFC) | Cortical | 99 |
| FreeSurfer Thickness | Area_hIP4_(IPS) | Cortical | 98 |
| FreeSurfer Thickness | Area_Op8_(Frontal_Operculum) | Cortical | 94 |
| FreeSurfer Thickness | SF_(Amygdala) | Cortical | 94 |
| FreeSurfer Thickness | Area_8v2_(MFG) | Cortical | 93 |
| FreeSurfer Thickness | Area_s32_(sACC) | Cortical | 87 |
| FreeSurfer Thickness | Area_SFS1_(SFS) | Cortical | 87 |
| FreeSurfer Thickness | Ch_4_(Basal_Forebrain) | Cortical | 82 |
| FreeSurfer Thickness | Area_STS1_(STS) | Cortical | 80 |
| FreeSurfer Thickness | Area_hIP3_(IPS) | Cortical | 72 |
| FreeSurfer Thickness | Area_5Ci_(SPL) | Cortical | 68 |
| **Processing** | **ROI** | **ROI Location** | **Mean SumR2** |
| FreeSurfer All Modalities | Right-Putamen | Subcortical | 1480 |
| FreeSurfer All Modalities | Left-Putamen | Subcortical | 1327 |
| FreeSurfer All Modalities | Left-Caudate | Subcortical | 1275 |
| FreeSurfer All Modalities | Right-Caudate | Subcortical | 1249 |
| FreeSurfer All Modalities | Right-Pallidum | Subcortical | 789 |
| FreeSurfer All Modalities | Left-Pallidum | Subcortical | 766 |
| FreeSurfer All Modalities | Left-Thalamus-Proper | Subcortical | 678 |
| FreeSurfer All Modalities | Right-Thalamus-Proper | Subcortical | 615 |
| FreeSurfer All Modalities | Right-Hippocampus | Subcortical | 611 |
| FreeSurfer All Modalities | Left-Hippocampus | Subcortical | 594 |
| FreeSurfer All Modalities | Left-Amygdala | Subcortical | 505 |
| FreeSurfer All Modalities | Right-Amygdala | Subcortical | 496 |
| FreeSurfer All Modalities | Left-Accumbens-area | Subcortical | 454 |
| FreeSurfer All Modalities | Right-Accumbens-area | Subcortical | 375 |
| FreeSurfer All Modalities | Area_hOc1_(V1,_17,_CalcS) | Cortical | 214 |
| FreeSurfer All Modalities | Area_3b_(PostCG) | Cortical | 197 |
| FreeSurfer All Modalities | Dorsal_Dentate_Nucleus_(Cerebellum) | Cortical | 195 |
| FreeSurfer All Modalities | Area_Fo5_(OFC) | Cortical | 193 |
| FreeSurfer All Modalities | Area_Ig1_(Insula) | Cortical | 191 |
| FreeSurfer All Modalities | Area_hOc3d_(Cuneus) | Cortical | 190 |
| FreeSurfer All Modalities | Area_8d1_(SFG) | Cortical | 189 |
| FreeSurfer All Modalities | Ventral_Dentate_Nucleus_(Cerebellum) | Cortical | 181 |
| FreeSurfer All Modalities | Area_4p_(PreCG) | Cortical | 178 |
| FreeSurfer All Modalities | Area_IFS4_(IFS) | Cortical | 173 |
| FreeSurfer All Modalities | Area_hOc4lp_(LOC) | Cortical | 172 |
| FreeSurfer All Modalities | Area_Id1_(Insula) | Cortical | 171 |
| FreeSurfer All Modalities | Area_OP3_(POperc) | Cortical | 169 |
| FreeSurfer All Modalities | Area_hOc4la_(LOC) | Cortical | 165 |
| FreeSurfer All Modalities | Area_OP4_(POperc) | Cortical | 165 |
| FreeSurfer All Modalities | Area_7A_(SPL) | Cortical | 164 |
| FreeSurfer All Modalities | Area_Op9_(Frontal_Operculum) | Cortical | 164 |
| FreeSurfer All Modalities | Area_TI_(STG) | Cortical | 164 |
| FreeSurfer All Modalities | Area_TPJ_(STG/SMG) | Cortical | 163 |
| FreeSurfer All Modalities | Area_3a_(PostCG) | Cortical | 160 |
| FreeSurfer All Modalities | Area_OP1_(POperc) | Cortical | 160 |
| FreeSurfer All Modalities | Area_OP2_(POperc) | Cortical | 159 |
| FreeSurfer All Modalities | Area_PFcm_(IPL) | Cortical | 158 |
| FreeSurfer All Modalities | Area_TE_1.1_(HESCHL) | Cortical | 156 |
| FreeSurfer All Modalities | Area_33_(ACC) | Cortical | 153 |
| FreeSurfer All Modalities | Area_hIP1_(IPS) | Cortical | 153 |
| FreeSurfer All Modalities | Area_Ig2_(Insula) | Cortical | 153 |
| FreeSurfer All Modalities | Area_Ig3_(Insula) | Cortical | 151 |
| FreeSurfer All Modalities | CA2_(Hippocampus) | Cortical | 151 |
| FreeSurfer All Modalities | CA3_(Hippocampus) | Cortical | 149 |
| FreeSurfer All Modalities | Area_hOc2_(V2,_18) | Cortical | 147 |
| FreeSurfer All Modalities | Area_IFS3_(IFS) | Cortical | 147 |
| FreeSurfer All Modalities | Area_44_(IFG) | Cortical | 146 |
| FreeSurfer All Modalities | Area_IFS1_(IFS) | Cortical | 146 |
| FreeSurfer All Modalities | Area_IFS2_(IFS) | Cortical | 146 |
| FreeSurfer All Modalities | Area_6d2_(PreCG) | Cortical | 145 |
| FreeSurfer All Modalities | Area_FG2_(FusG) | Cortical | 145 |
| FreeSurfer All Modalities | Area_PFop_(IPL) | Cortical | 145 |
| FreeSurfer All Modalities | Area_TE_2.1_(STG) | Cortical | 145 |
| FreeSurfer All Modalities | Area_Fo7_(OFC) | Cortical | 144 |
| FreeSurfer All Modalities | Area_Id6_(Insula) | Cortical | 144 |
| FreeSurfer All Modalities | Area_FG1_(FusG) | Cortical | 143 |
| FreeSurfer All Modalities | Area_IFJ2_(IFS,PreCS) | Cortical | 143 |
| FreeSurfer All Modalities | Area_TeI_(STG) | Cortical | 141 |
| FreeSurfer All Modalities | Area_6ma_(preSMA,_mesial_SFG) | Cortical | 140 |
| FreeSurfer All Modalities | Area_Fp2_(FPole) | Cortical | 140 |
| FreeSurfer All Modalities | Interposed_Nucleus_(Cerebellum) | Cortical | 140 |
| FreeSurfer All Modalities | Area_PFt_(IPL) | Cortical | 138 |
| FreeSurfer All Modalities | Area_6d1_(PreCG) | Cortical | 137 |
| FreeSurfer All Modalities | Area_Id5_(Insula) | Cortical | 135 |
| FreeSurfer All Modalities | Area_45_(IFG) | Cortical | 134 |
| FreeSurfer All Modalities | Area_PF_(IPL) | Cortical | 134 |
| FreeSurfer All Modalities | Area_TE_2.2_(STG) | Cortical | 134 |
| FreeSurfer All Modalities | Area_Fo6_(OFC) | Cortical | 133 |
| FreeSurfer All Modalities | Area_p24c_(pACC) | Cortical | 131 |
| FreeSurfer All Modalities | Subiculum_(Hippocampus) | Cortical | 131 |
| FreeSurfer All Modalities | Area_Fo4_(OFC) | Cortical | 130 |
| FreeSurfer All Modalities | Tuberculum_(Basal_Forebrain) | Cortical | 130 |
| FreeSurfer All Modalities | Area_1_(PostCG) | Cortical | 129 |
| FreeSurfer All Modalities | Area_6d3_(SFS) | Cortical | 129 |
| FreeSurfer All Modalities | Area_hOc5_(LOC) | Cortical | 129 |
| FreeSurfer All Modalities | Ch_123_(Basal_Forebrain) | Cortical | 129 |
| FreeSurfer All Modalities | Fastigial_Nucleus_(Cerebellum) | Cortical | 129 |
| FreeSurfer All Modalities | Area_hIP7_(IPS) | Cortical | 128 |
| FreeSurfer All Modalities | Area_hOc4d_(Cuneus) | Cortical | 127 |
| FreeSurfer All Modalities | Area_IFJ1_(IFS,PreCS) | Cortical | 127 |
| FreeSurfer All Modalities | Area_7P_(SPL) | Cortical | 126 |
| FreeSurfer All Modalities | Area_Id4_(Insula) | Cortical | 125 |
| FreeSurfer All Modalities | Area_4a_(PreCG) | Cortical | 123 |
| FreeSurfer All Modalities | Area_hIP6_(IPS) | Cortical | 123 |
| FreeSurfer All Modalities | Area_Id3_(Insula) | Cortical | 123 |
| FreeSurfer All Modalities | Area_2_(PostCS) | Cortical | 121 |
| FreeSurfer All Modalities | Area_TE_1.2_(HESCHL) | Cortical | 121 |
| FreeSurfer All Modalities | DG_(Hippocampus) | Cortical | 119 |
| FreeSurfer All Modalities | Area_p24ab_(pACC) | Cortical | 117 |
| FreeSurfer All Modalities | HC-Transsubiculum_(Hippocampus) | Cortical | 115 |
| FreeSurfer All Modalities | Area_Fp1_(FPole) | Cortical | 112 |
| FreeSurfer All Modalities | Area_7M_(SPL) | Cortical | 111 |
| FreeSurfer All Modalities | Area_Id2_(Insula) | Cortical | 111 |
| FreeSurfer All Modalities | Area_hOc3v_(LingG) | Cortical | 110 |
| FreeSurfer All Modalities | Area_8v1_(MFG) | Cortical | 108 |
| FreeSurfer All Modalities | Area_hOc6_(POS) | Cortical | 107 |
| FreeSurfer All Modalities | LB_(Amygdala) | Cortical | 106 |
| FreeSurfer All Modalities | Area_TE_3_(STG) | Cortical | 105 |
| FreeSurfer All Modalities | Area_Ia1_(Insula) | Cortical | 104 |
| FreeSurfer All Modalities | CM_(Amygdala) | Cortical | 104 |
| FreeSurfer All Modalities | Area_6mp_(SMA,_mesial_SFG) | Cortical | 102 |
| FreeSurfer All Modalities | IF_(Amygdala) | Cortical | 102 |
| FreeSurfer All Modalities | Terminal_islands_(Basal_Forebrain) | Cortical | 102 |
| FreeSurfer All Modalities | Area_PGa_(IPL) | Cortical | 101 |
| FreeSurfer All Modalities | VTM_(Amygdala) | Cortical | 101 |
| FreeSurfer All Modalities | Area_FG4_(FusG) | Cortical | 98 |
| FreeSurfer All Modalities | Area_PFm_(IPL) | Cortical | 98 |
| FreeSurfer All Modalities | Area_8d2_(SFG) | Cortical | 97 |
| FreeSurfer All Modalities | Area_hIP2_(IPS) | Cortical | 97 |
| FreeSurfer All Modalities | Area_hIP8_(IPS) | Cortical | 97 |
| FreeSurfer All Modalities | Area_PGp_(IPL) | Cortical | 97 |
| FreeSurfer All Modalities | Area_STS2_(STS) | Cortical | 97 |
| FreeSurfer All Modalities | HATA_(Hippocampus) | Cortical | 97 |
| FreeSurfer All Modalities | Area_hIP5_(IPS) | Cortical | 96 |
| FreeSurfer All Modalities | MF_(Amygdala) | Cortical | 95 |
| FreeSurfer All Modalities | Area_FG3_(FusG) | Cortical | 94 |
| FreeSurfer All Modalities | Area_hIP4_(IPS) | Cortical | 90 |
| FreeSurfer All Modalities | CA1_(Hippocampus) | Cortical | 89 |
| FreeSurfer All Modalities | Area_hOc4v_(LingG) | Cortical | 87 |
| FreeSurfer All Modalities | Area_7PC_(SPL) | Cortical | 86 |
| FreeSurfer All Modalities | Area_hPO1_(POS) | Cortical | 86 |
| FreeSurfer All Modalities | Area_25_(sACC) | Cortical | 84 |
| FreeSurfer All Modalities | Area_5M_(SPL) | Cortical | 84 |
| FreeSurfer All Modalities | Area_s24_(sACC) | Cortical | 83 |
| FreeSurfer All Modalities | Area_SFS1_(SFS) | Cortical | 83 |
| FreeSurfer All Modalities | Area_5L_(SPL) | Cortical | 81 |
| FreeSurfer All Modalities | Area_TE_1.0_(HESCHL) | Cortical | 80 |
| FreeSurfer All Modalities | Entorhinal_Cortex | Cortical | 76 |
| FreeSurfer All Modalities | SF_(Amygdala) | Cortical | 76 |
| FreeSurfer All Modalities | Area_p32_(pACC) | Cortical | 75 |
| FreeSurfer All Modalities | Area_8v2_(MFG) | Cortical | 74 |
| FreeSurfer All Modalities | Area_Id7_(Insula) | Cortical | 74 |
| FreeSurfer All Modalities | Area_Fo3_(OFC) | Cortical | 73 |
| FreeSurfer All Modalities | Area_Fo2_(OFC) | Cortical | 68 |
| FreeSurfer All Modalities | Area_Op8_(Frontal_Operculum) | Cortical | 67 |
| FreeSurfer All Modalities | Area_Fo1_(OFC) | Cortical | 66 |
| FreeSurfer All Modalities | Area_s32_(sACC) | Cortical | 62 |
| FreeSurfer All Modalities | Area_STS1_(STS) | Cortical | 61 |
| FreeSurfer All Modalities | Ch_4_(Basal_Forebrain) | Cortical | 61 |
| FreeSurfer All Modalities | Area_hIP3_(IPS) | Cortical | 55 |
| FreeSurfer All Modalities | Area_5Ci_(SPL) | Cortical | 51 |

Supplementary Figure 4 :  **Brain regions contribution to morphometricity estimates of potential confounders, for each processing.** As CAT12 Surface captures signal from the cortical thickness only, we did not include it in this plot. For all three volume based processing we subdivided the brain into 3 regions : cortical, subcortical and cerebellar. As for Free Surfer, we subdivided it into cortical thickness, cortical area and subcortical region.For instance FSLVBM explained a variance of 0.75 for the covariates `Discrepancy between T1 and template`. The cortical area accounted for 68% of this signal vs. 21% for the subcortical area and 11% for the cerebellum.

Supplementary Figure 5 : **Brain regions contribution to morphometricity estimates of traits of interest, for each processing, controlling for covariates.** As CAT12 Surface captures signal from the cortical thickness only, we did not include it in this plot. For all three volume-based processing we subdivided the brain into 3 regions: cortical, subcortical and cerebellar. As for Free Surfer, we subdivided it into cortical thickness, cortical area and subcortical region. Regions exhibited not significant contribution remained unlabelled.


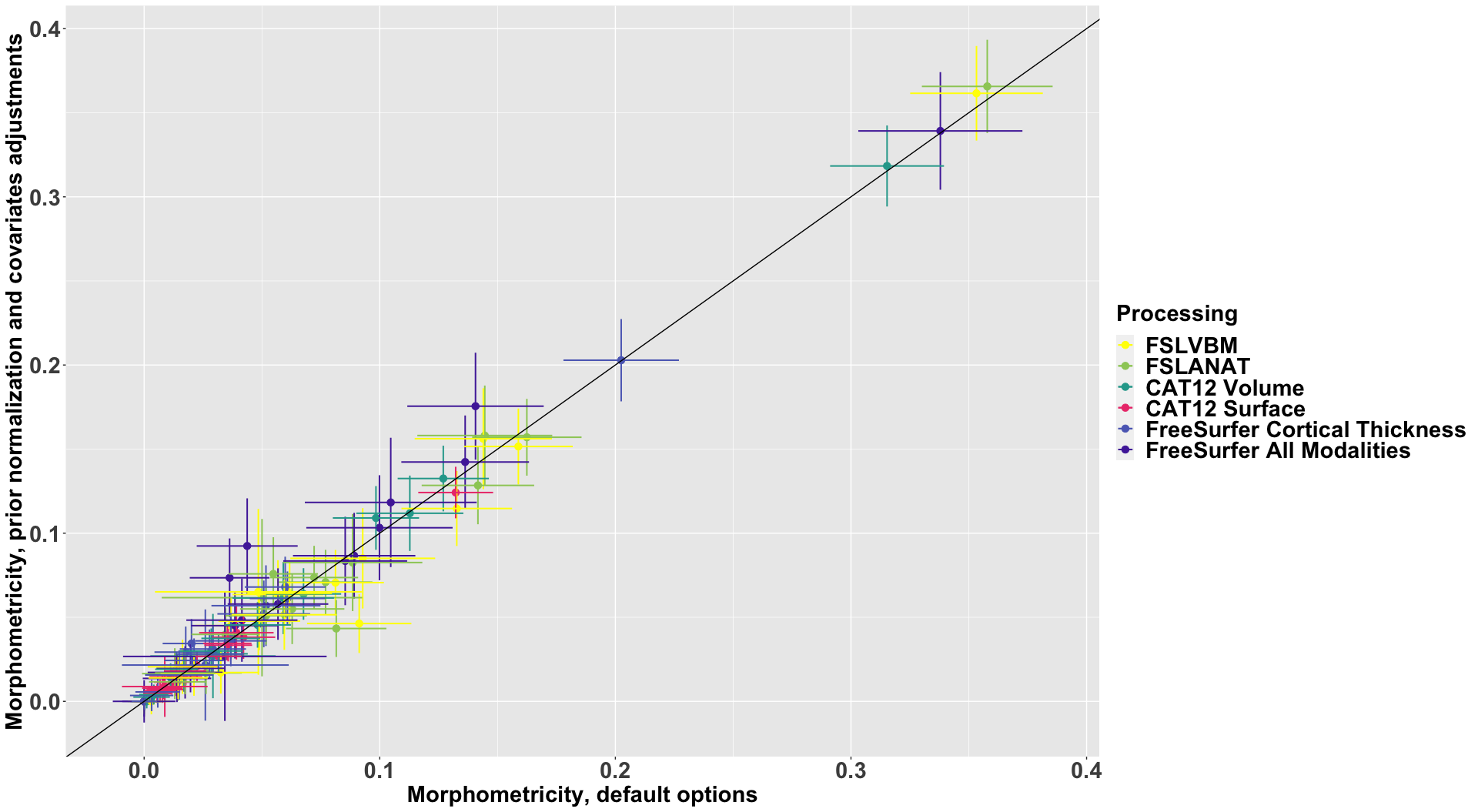


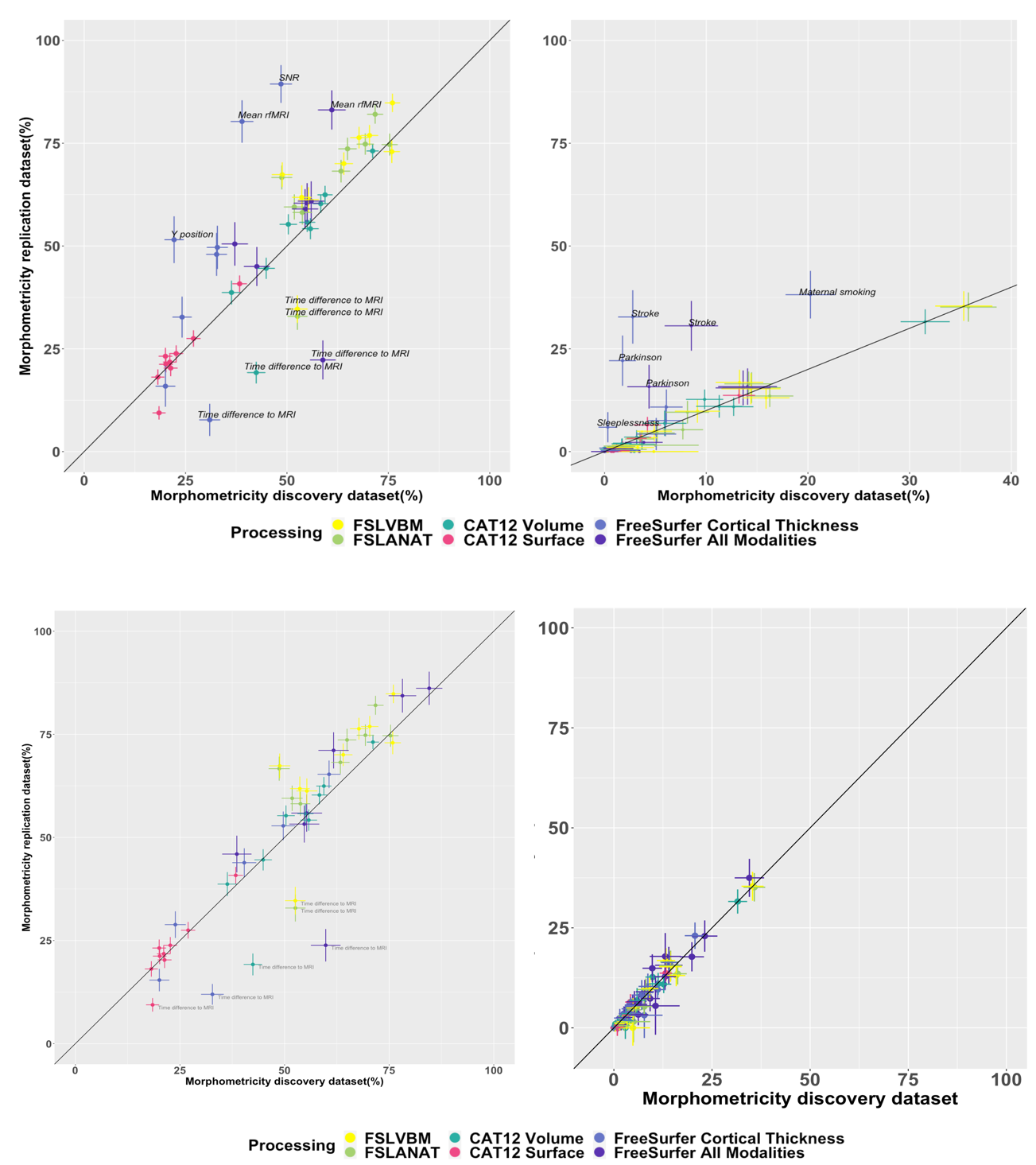
Supplementary Figure 6 : **Concordance of morphometricity estimates between brain measurements and after rank inverse normalization of brain measurements.** Morphometricity from default brain measurements is shown as the x-axis and morphometricity from rank-normalized voxels/vertices is shown as the y-axis. The vertical and horizontal bars show the 95% confidence intervals in the two samples.

Supplementary Figure 7 : **Concordance of Morphometricity estimates between the replication and discovery sample.** Morphometricity in the discovery sample is shown as the x-axis and morphometricity in the replication dataset is shown as the y-axis. The vertical and horizontal bars show the 95% confidence intervals in the two samples. The left panel shows the morphometricity estimates of possible confounders (controlling for standard covariates). The right panel depicts morphometricities of the traits of interest when controlling for all covariates (standard and confounders).


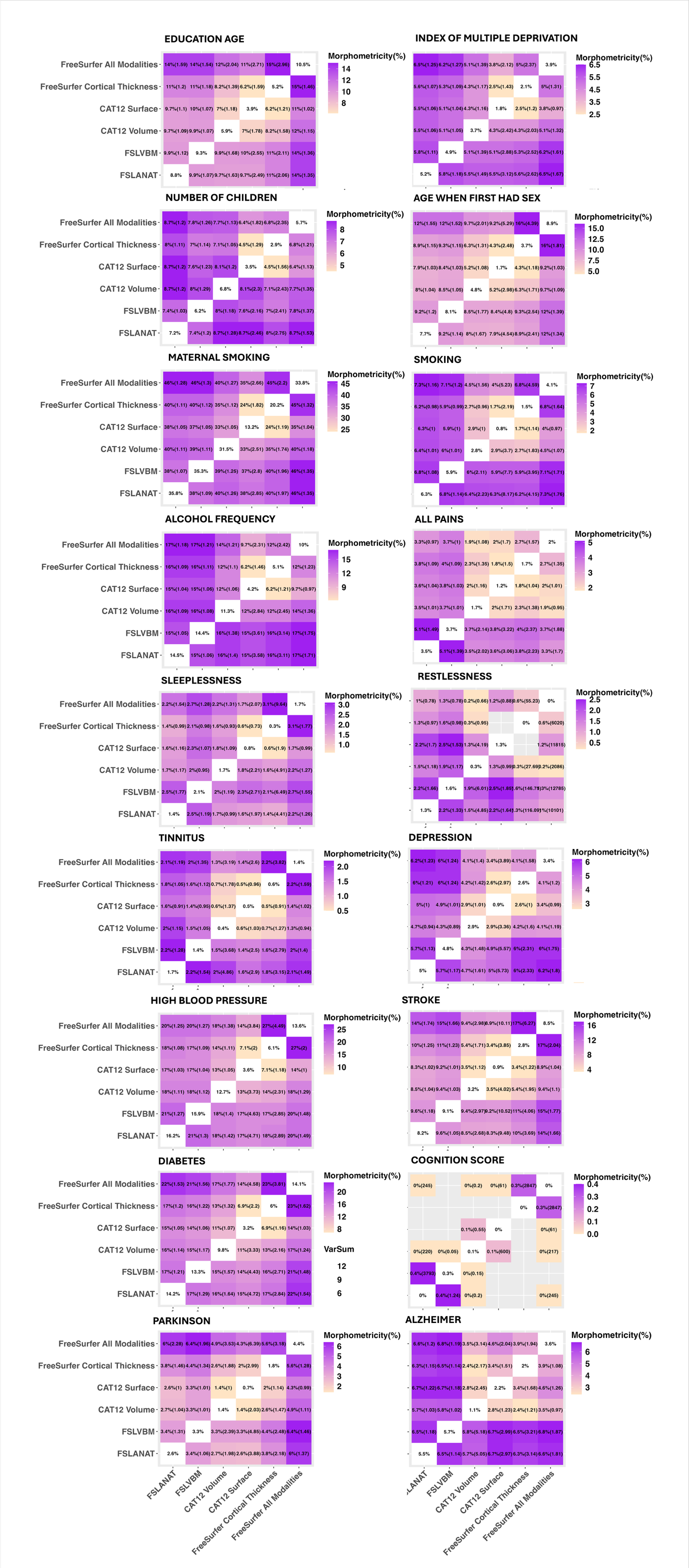


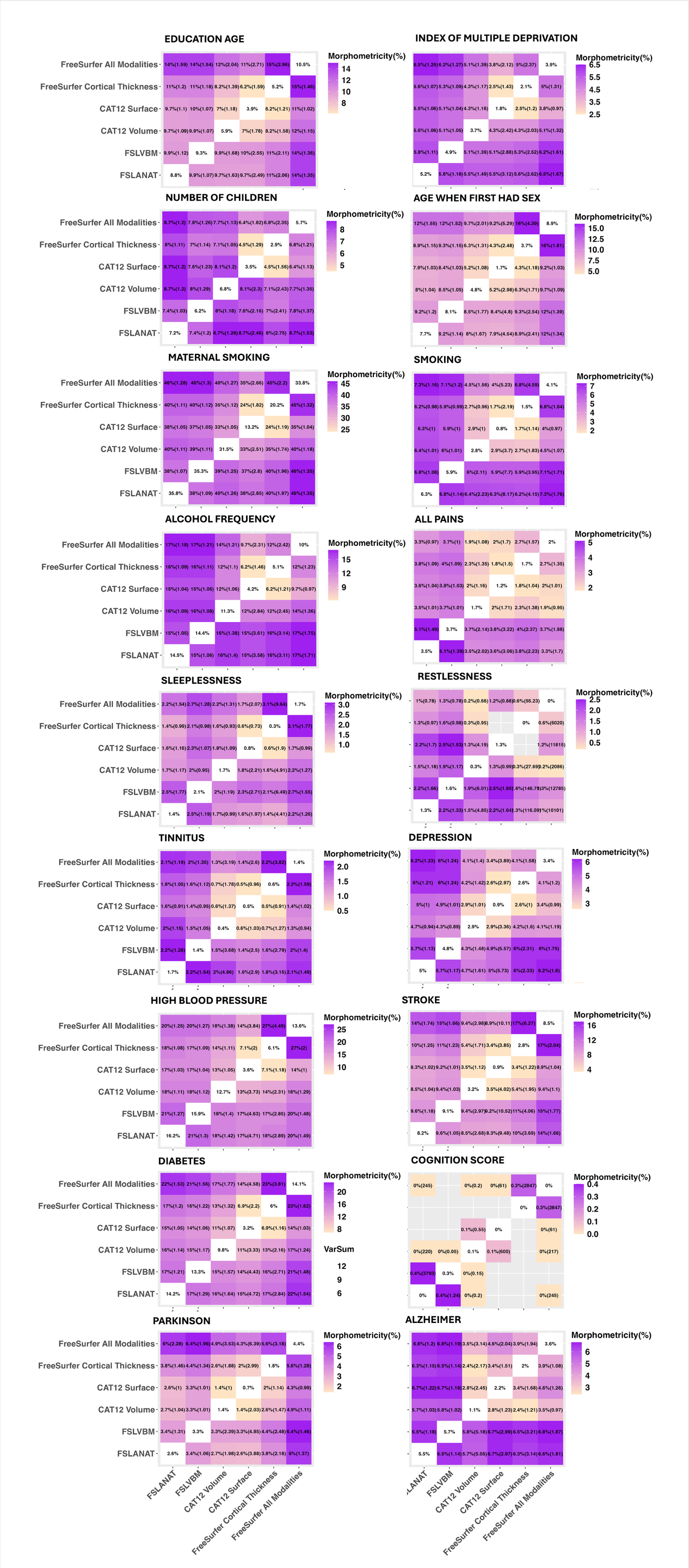


Supplementary Figure 8 : **Heatmap of morphometricity** i**ncrease when fitting two grey-matter representation in the model, across all traits of interest.** Each heatmap illustrates the percentage of variance explained (morphometricity) when combining two processing methods. The rate of increase is shown in parentheses, and diagonal values represents the morphometricity of each processing method alone. Rows indicate the reference processing and columns the added processing. For example, on the Alcohol frequency plot, the element at coordinates (1,2) corresponds to the result of of FreeSurfer All Modalities added to FSLVBM, resulting in an estimate 1.21 higher than FSLVBM alone (14.4%). Symmetrically, adding FSLVBM to FreeSurfer All Modalities yields a different rate of increase (1.75) since both processing alone do not explain the same percentage of variance.


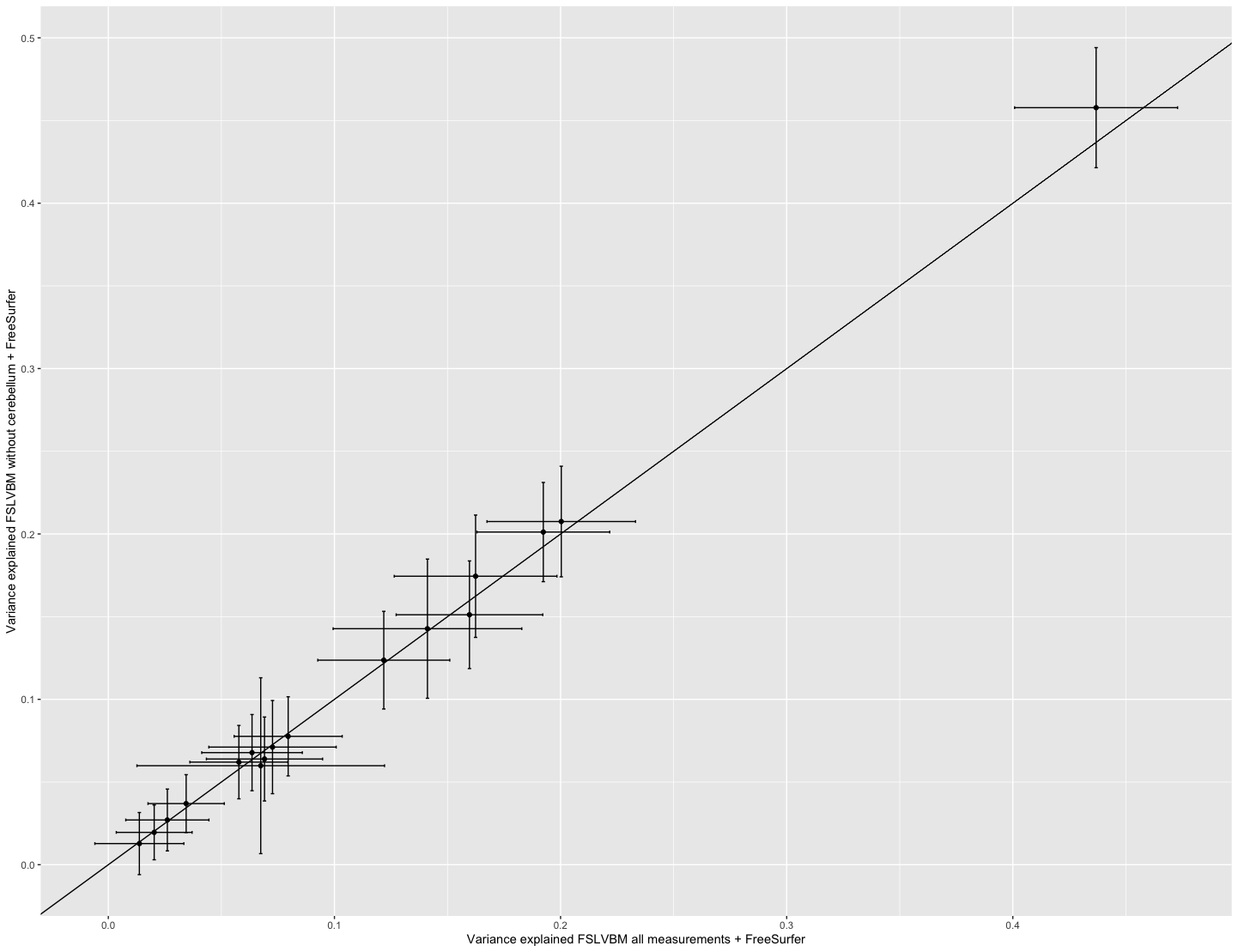


Supplementary Figure 9 : **Role of cerebellar measurements in FSLVBM morphometricity estimates.** We found that all processing do not capture the same signal with some part being unique. As FreeSurfer do not measure the cerebellum, we wondered whether this discrepancy originated from that region. We compared morphometricity estimates when adding FSL (all measurements) and Free Surfer vs. FSL (without cerebellar measurements) and FreeSurfer. The vertical and horizontal bars show the 95% confidence intervals

Supplementary Figure 10 : **Q-Q plot of the minimal p-values for each voxel/vertex across the 1000 random traits without any adjustments (left panel) and with prior normalization (right panel).** Minimal p-value are presented in the log10 scale. Grey lines show the 95% confidence interval. Therefore, processing crossing this threshold mean that they exhibit p-values larger/smaller than expected by chance.

Supplementary Figure 13 : **Number of regions of interest exhibiting at least one significant cluster, across all traits.**

Supplementary Figure 14 : **Heatmap of number of common significant regions of interest, for top 4 traits exhibiting higher number of significant clusters.** Each heatmap depicts the number of regions of interest exhibiting at least one significant cluster for both processing method. Diagonal values represents the total number of significant cluster for each processing. For example, in the Maternal smoking plot, the element (3,1) is 32 indicating that 32 same regions of interest were significant for both CAT12 Volume and FSLVBM processing, whereas FSLVBM captures 55 significant clusters and CAT12 Volume 67.


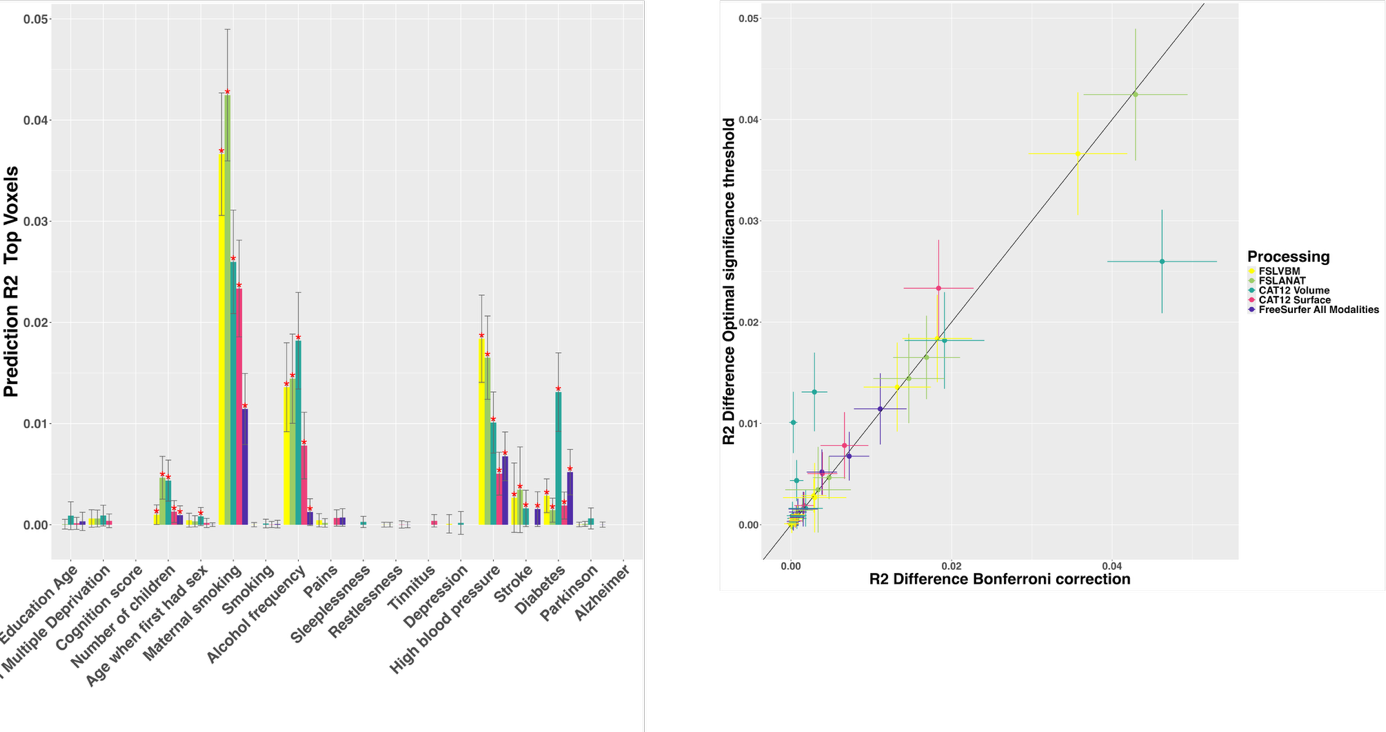


Supplementary Figure 15 : **Linear prediction from top significant voxels/vertices, across all traits and processing methods, after optimal thresholds correction, and comparison with Bonferroni correction.** The left panel depicts the R2 prediction from top significant voxels/vertices with optimal thresholds correction : the y-axis represents the difference between the R^2^prediction from the model with top voxels/ vertices minus R^2^ of baseline model. Red stars indicate significant log-likelihood ratio test after Bonferroni correction (p < 0.05/6*29). The right panel depicts the R2 comparison when using Bonferroni correction vs. optimal thresholds


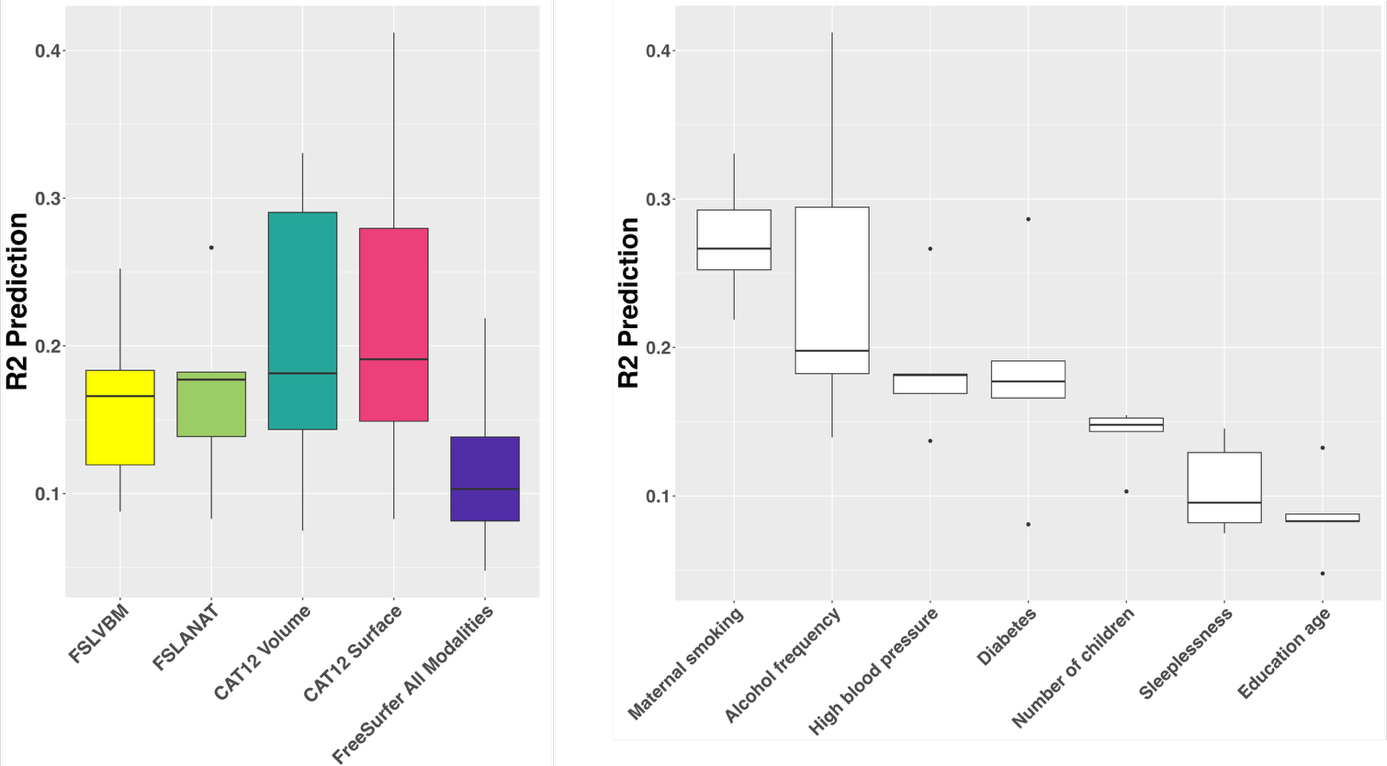


Supplementary Figure 16 : **Fraction of predicted morphometricity across traits exhibiting significant prediction, for all processing methods.** Left panel represents the fraction of predicted morphometricity (e.g the R2 prediction divided by the morphometricity for this trait and processing) across all 6 traits included in the right panel. Right panel represents the fraction of predicted morphometricity for the 6 traits exhibiting significant prediction (Figure 7), across all six processing methods.


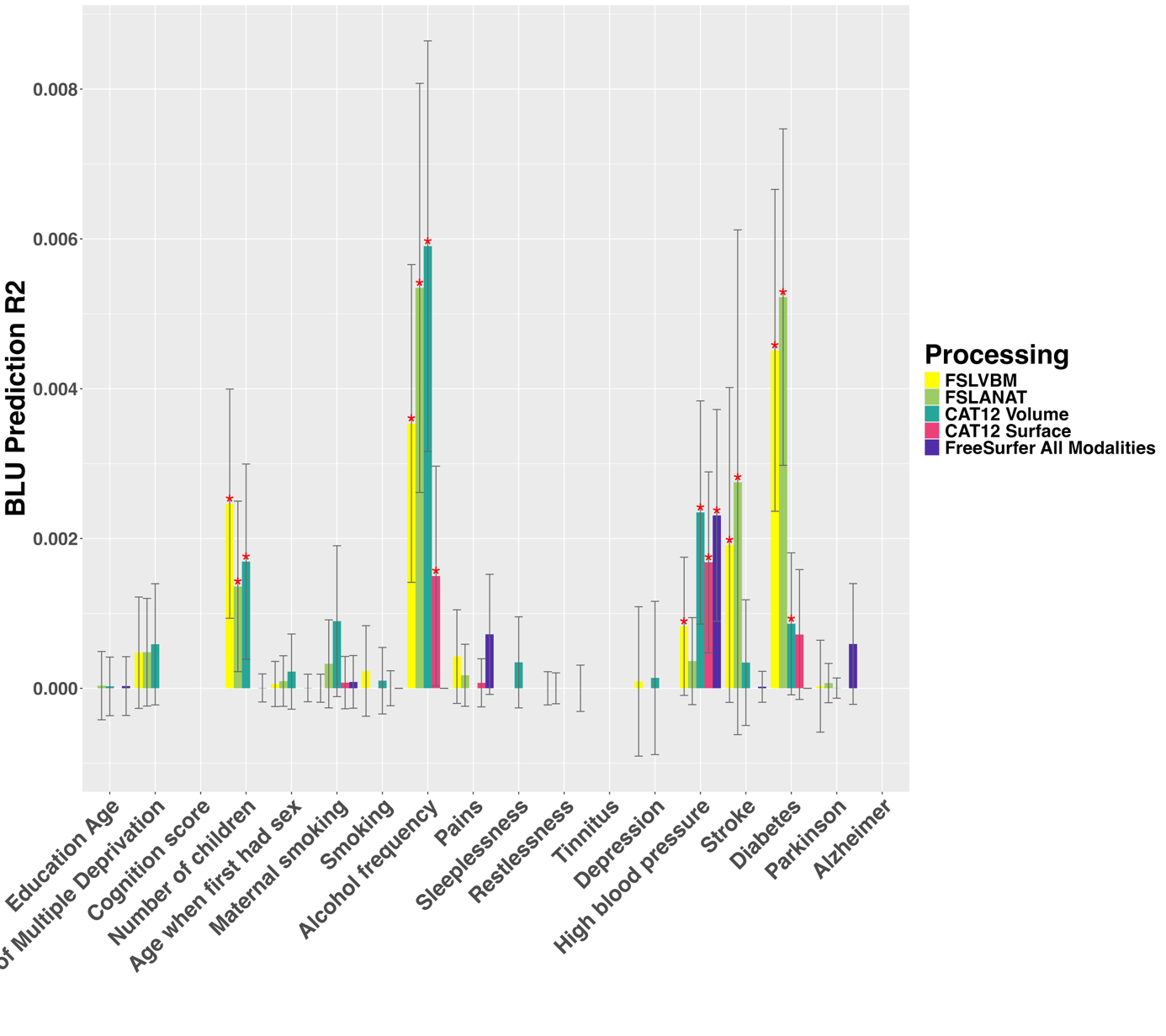


Supplementary Figure 17 : **Linear prediction from size one clusters, across all traits and processing methods.**

Supplementary Figure 18 : **Replicating cluster locations by Region of Interest for Diabetes.**

Supplementary 19: **Replicating cluster locations by Region of Interest for High blood pressure.**

Supplementary Figure 20 : **Replicating cluster locations by Region of Interest for Alcohol frequency.**

Supplementary Figure 21: **Replicating cluster locations by Region of Interest for Number of children.**

Supplementary Figure 22: **Replicating cluster locations by Region of Interest for Stroke.**

Supplementary Appendix 1: Linear Mixed Model to estimate morphometricity

$$\mathbf{Y}\mathbf{=}\boldsymbol{X\beta}\mathbf{+}\mathbf{b}\mathbf{+}\mathbf{e}\mathbf{(}\mathbf{Eq. 1}\mathbf{)}$$

With Y_N,1_ a vector containing N observations of our trait of interest

X_N,c_ a matrix of c covariates

β_c,1_ a vector of fixed effects

b a vector of brain random effects with b ∼ N(0,Bσ^2^b) and σ_b_^2^ the total trait variance captured by all voxel or vertex-wise measurements

e a vector of error terms with e ∼ N(0, Iσ^2^e) and σ_e_^2^ the residual variance accounted for by the term error.

I the identity matrix

B is a matrix of variance-covariance between individuals calculated from all standardized brain measurements which we will refer to as a Brain Relatedness Matrix (BRM)[9]. These BRM are calculated with OSCA[29]. We utilized information contained in BRM to perform QC and resulted in 172 participants excluded due to extreme/outlying BRM-values (**SFigure 3**).

Finally, morphometricity is expressed as an R^2^, which quantifies the proportion of variance explained by the brain measurements:

$$R^{2}=\frac{\sigma_{b}^{2}}{\sigma_{e}^{2}+\sigma_{b}^{2}} (\mathbf{Eq. 2)}$$

Supplementary Appendix 2 : Extension of LMM

We fitted a model (**Eq. 3**) with two random effects $b_{Processing 1}$ and $b_{Processing 2}$, each corresponding to a processing. We compared this model to a reduced (nested) model containing only a single random effect (**Eq. 1**), using a likelihood ratio test, which follows a chi-square distribution.

$$\mathbf{Y}\mathbf{=}\boldsymbol{X\beta}\mathbf{+}\boldsymbol{b}_{\boldsymbol{Processing 1}}\mathbf{+}\boldsymbol{b}_{\boldsymbol{Processing 2}}\mathbf{+}\mathbf{e}\mathbf{(Eq.}\mathbf{3}\mathbf{)}$$

With b_i_ ∼ N(0, Biσ^2^bi), $i\in(Processing 1, Processing 2)$ and all other parameters left unchanged.

As surface-based processing does not provide cerebellum measurements, we tested whether the complementary proportion of trait variance comes from the cerebellum. We performed sensitivity analyses that focused on FSLVBM (without cerebellum measurements) and FreeSurfer.

We also applied LMM with multiple random effects to decompose the morphometricity into the (conditional) contributions of the cortical, subcortical and cerebellar (when available) measurements, the model then becomes :

$$\mathbf{Y}\mathbf{=}\boldsymbol{X\beta}\mathbf{+}\boldsymbol{b}_{\boldsymbol{Cortical}}\mathbf{+}\boldsymbol{b}_{\boldsymbol{Subcortical}}\mathbf{+}\boldsymbol{b}_{\boldsymbol{Cerebellar}}\mathbf{+}\mathbf{e}\mathbf{(Eq.}\mathbf{4}\mathbf{)}$$

This model extends the LMM hypothesis of a single normal distribution of effects to incorporate three distributions, each corresponding to different part of the grey-matter. For each part, we reported the proportion of variance captured, which collectively should sum to the overall morphometricity.
